# Dynamics of global mpox epidemics, 1970–2025: a systematic review and meta-analysis of evolving transmissibility and intervention effects

**DOI:** 10.1136/bmjgh-2025-022252

**Published:** 2026-08-20

**Authors:** Yin-Chien Lin, Tzai-Hung Wen, Wei-Liang Shih, Chia-Wei Liao, Sten H Vermund, Chi-Tai Fang

**Affiliations:** 1 Institute of Epidemiology and Preventive Medicine, National Taiwan University College of Public Health, Taipei City, Taiwan; 2 Department of Geography, National Taiwan University, Taipei City, Taiwan; 3 Infectious Diseases Research and Education Center, Ministry of Health and Welfare and National Taiwan University, Taipei City, Taiwan; 4 Office of the Dean, University of South Florida College of Public Health, Tampa, Florida, USA; 5 Department of Pediatrics, University of South Florida Morsani College of Medicine, Tampa, Florida, USA; 6 Global Virus Network, Tampa, Florida, USA; 7 Department of Internal Medicine, National Taiwan University Hospital and National Taiwan University School of Medicine, Taipei City, Taiwan

**Keywords:** Global Health, Disease Outbreaks, Immunisation, Systematic review, Infections, diseases, disorders, injuries

## Abstract

**Background:**

The 2022 global mpox epidemic declined before modified Vaccinia Ankara–Bavarian Nordic (MVA-BN) vaccines were widely deployed, contributing to the perception that transmission was self-limiting within a small high-risk population. This may have delayed global vaccine allocation and weakened responses to the resurgence that has disproportionately affected Africa since 2024. The reasons for the 2022 decline remain uncertain, and prior studies have reported widely divergent estimates of vaccination impact. We systematically reviewed and meta-analysed the evolving transmissibility of mpox clades and the effects of interventions.

**Methods:**

We searched GenBank, PubMed and Embase through 18 May 2025, for mpox virus sequences, reproduction number (R) estimates and modelling studies evaluating intervention effectiveness. Two reviewers independently assessed eligibility, extracted data and evaluated risk of bias using a validated tool. Random-effects meta-analyses were performed.

**Results:**

Fifty-two studies reporting R estimates and 40 studies evaluating intervention effectiveness met eligibility criteria. For clade I mpox, pooled R increased from 0.71 (95% CI 0.26 to 1.17) during 1970–2017 to 1.23 (1.12–1.33) for subclades Ib/Ia after 2023 (p=0.0303). For clade II mpox, pooled R was 1.11 (0.90–1.32) during 2017–2021 and increased to 2.66 (2.31–3.00) for subclade IIb in 2022–2023 (p<0.0001). During the 2022 subclade IIb outbreak, empirical modelling studies estimated that behaviour change and vaccination together were associated with a substantial reduction in mpox cases (55%, 95% CI 37 to 73; I^²^=81.2%), although effect sizes varied across settings according to the extent of behaviour change and the timing and coverage of vaccine rollout.

**Conclusions:**

Behaviour change and vaccination likely played important roles in the decline of the 2022 mpox epidemic in many studied settings. Given the stepwise increase in human-to-human transmissibility associated with the emergence of new subclades, effective mpox epidemic control requires proactive, rapid and equitable vaccine rollout supported by culturally tailored risk communication.

**PROSPERO registration number:**

CRD420250653072.

**WHAT IS ALREADY KNOWN ON THIS TOPIC**

- Studies of mpox virus phylogeny and reproductive number (R) have largely been outbreak-focused and cross-sectional. Empirical modelling studies during the 2022 global outbreak yielded conflicting estimates of the impact of vaccination campaigns, ranging from negligible effect to 79% of cases averted. Overall, evidence remains fragmented on the evolving threat posed by mpox and the effectiveness of vaccination campaigns.

**WHAT THIS STUDY ADDS**

- This is the first systematic review and meta-analysis to longitudinally track mpox transmissibility and evaluate the effectiveness of vaccination for outbreak control.
- Along distinct evolutionary trajectories, R for clade I mpox increased from 0.71 (95% CI 0.26 to 1.17) during 1970–2017 to 1.23 (1.12–1.33) for subclades Ib/Ia since 2023, whereas R for clade II increased from 1.11 (0.90–1.32) during 2017–2021 to 2.66 (2.31–3.00) for subclade IIb in 2022–2023.
- Behaviour change and vaccination likely played important roles in the decline of the 2022 mpox epidemic in many studied settings; empirical modelling studies estimated substantial case reductions, although effect sizes varied across settings according to vaccine rollout timing, coverage, and the extent of behaviour change.

**HOW THIS STUDY MIGHT AFFECT RESEARCH, PRACTICE OR POLICY**

- Given the stepwise increase in human-to-human transmissibility associated with the emergence of new subclades, effective outbreak control requires rapid, equitable vaccine rollout coupled with risk communication and community engagement. These findings provide timely, policy-relevant evidence for the WHO, the Africa Centres for Disease Control and Prevention (Africa CDC) and national authorities to guide equitable implementation of prevention measures to contain the ongoing mpox resurgence and mitigate its intersection with other epidemics, particularly HIV and tuberculosis.

## Introduction

 Mpox is a viral zoonosis caused by the mpox virus (MPXV), endemic in Central and West Africa since the first human case was identified in the 1970s.^1^ In May 2022, an unprecedented global outbreak emerged, characterised by sustained human-to-human transmission of clade IIb MPXV, primarily affecting men who have sex with men (MSM) through close, often sexual, contact, and rapidly spreading to more than 110 countries.^2^ In response, public health authorities initiated vaccination campaigns using stockpiled modified Vaccinia Ankara–Bavarian Nordic (MVA-BN) vaccines, originally developed for preparedness against smallpox bioterrorism,^3^ and many at-risk individuals modified their behaviours to reduce the risk of acquisition. Before vaccines were widely deployed, however, new cases in Europe and North America began to decline in July–August 2022, contributing to the perception that the downturn resulted from the depletion of susceptible individuals within a very small high-risk population rather than from the impact of interventions.^4^ The WHO ended its mpox Public Health Emergency of International Concern (PHEIC) in May 2023. The reasons for the 2022 decline remain uncertain, as previous studies have reported widely divergent estimates of vaccination impact, ranging from negligible to 79% of cases averted,^5 6^ with the sources of this variation still unclear.

In 2024, a sharp increase in mpox infections in the Democratic Republic of the Congo (DRC) and neighbouring countries, some of which had not previously reported mpox cases, prompted the WHO to declare a new PHEIC on 14 August 2024.^7^ The perception that the 2022 global mpox outbreak was self-limiting within a very small high-risk group may have delayed global vaccine allocation and potentially contributed to the slow global response to this resurgence. The ongoing epidemic in Africa presents additional complexity, with mixed modes of transmission, involvement of both adults and children and the emergence of a novel, sexually associated subclade Ib.^7^ Although the PHEIC was lifted on 5 September 2025, mpox persists as a Public Health Emergency of Continental Security (PHECS) under a high-alert status.

In this study, we systematically reviewed and meta-analysed the evolving transmissibility of mpox clades and the impact of vaccination and behaviour change, using empirical data from outbreaks worldwide. We aimed to generate robust evidence to inform global responses to the ongoing epidemic and to strengthen preparedness for future outbreaks.

## Methods

This systematic review and meta-analysis was conducted in accordance with Preferred Reporting Items for Systematic Reviews and Meta-Analyses (PRISMA) guidelines. The protocol was registered with the International Prospective Register of Systematic Reviews (PROSPERO: CRD420250653072) before the work began.

### Search strategy and selection criteria

We systematically searched GenBank, PubMed and Embase through 18 May 2025, for: (1) MPXV genome sequences; (2) studies estimating the reproduction number (R) of mpox; and (3) modelling studies assessing the effectiveness of interventions during the 2022–2023 outbreak. Detailed search terms and inclusion/exclusion criteria are provided in online supplemental text A. Two reviewers (Y-CL and C-TF) independently screened titles, abstracts, and full texts for eligibility. All discrepancies were resolved through discussion and consensus.

### MPXV sequence sampling strategy

Our phylogenetic analysis aimed to contextualise transmissibility estimates (R) along the evolutionary history of MPXV over the past five decades, rather than to reconstruct the full genomic diversity of all publicly available sequences. Because publicly available sequence databases are heavily dominated by genomes from the 2022 global outbreak, inclusion of all sequences would have substantially over-represented that outbreak. We, therefore, used stratified random sampling to assemble a sequence set balanced across major clades, subclades, collection periods and geographic regions, thereby providing balanced coverage of the evolutionary and epidemiological diversity relevant to our study objective. To assess the robustness of this sampling strategy, we conducted sensitivity analyses based on repeated random resampling using the same stratified procedure.

### Inclusion criteria

#### MPXV genome sequences

All complete MPXV genome sequences in GenBank were eligible. To construct a global phylogenetic tree spanning the past five decades, sequences were randomly selected using a stratified sampling approach based on clade, subclade, collection period and geographic region; the detailed categorisation and sampling process are provided in online supplemental text A.1.

#### Reproduction number (R) estimates

Studies estimating basic (R_0_), effective (R_e_) or time-dependent (R_t_) reproduction numbers of mpox using empirical data were eligible. We included in the meta-analysis estimates reflecting the intrinsic transmissibility of MPXV clades, rather than transmission dynamics influenced by depletion of susceptibles (see online supplemental text A.2 for detailed criteria for inclusion in the review and in the quantitative synthesis).

#### Modelling studies of intervention effectiveness

Eligible studies assessed the impact of interventions (eg, vaccination, risk-reducing behaviour change) on the mpox outbreak. Empirical modelling studies, that is, those calibrated to real-world data, that estimated the proportion of cases averted (hereafter referred to as prevention percentage) by vaccination, with or without concurrent behaviour change, relative to counterfactual scenarios without interventions during the 2022–2023 outbreak, were included in the meta-analysis (see online supplemental text A.3 for detailed criteria for inclusion in the review and in the quantitative synthesis).

### Data extraction and assessment for risk of bias

All included studies were reviewed in full. Two reviewers (Y-CL and C-TF) independently extracted information from the included studies (online supplemental texts A and B for extraction details). All studies were narratively synthesised. Those meeting criteria for quantitative synthesis underwent meta-analysis (online supplemental text B). Risk of bias was assessed using a validated 7-item tool previously applied in a systematic review of transmission models and epidemiological parameters of Marburg virus disease (online supplemental text C).^8^

### Phylogenetic analysis

We aligned MPXV genome sequences using NextClade (V.3.3.1)^9^ against the reference genome MPXV-M5312_HM12_Rivers (GenBank accession NC063383.1). A maximum-likelihood phylogenetic tree was constructed with 100 bootstrap replicates using the TN93+G+I substitution model in MEGA (V.11.0.13).^10^

### Data analysis

We performed inverse variance-weighted meta-analyses using random-effects models with the DerSimonian and Laird method in the meta package in R.^11^ Forest plots were used to present pooled estimates of mpox R and prevention percentages from vaccination campaigns (with or without concurrent behaviour change), and their 95% CIs. R estimates were grouped by MPXV clade and time period: clade I (1970–2017), clade II (2017–2021), clade IIb (2022–2023 global outbreak) and clade Ib/Ia (post-2023) and were meta-analysed separately within each clade (ie, clade I and clade II) under a phylogenetic context. Subgroup differences were evaluated using Cochran’s Q test. We also compared prevention percentages for vaccination campaigns alone versus vaccination campaigns combined with behaviour change.

Heterogeneity across studies was assessed using the I^2^ statistic. We conducted leave-one-out sensitivity analyses to assess the robustness of pooled estimates. Publication bias was evaluated using funnel plots and Egger’s regression test. Certainty of evidence was assessed using the Grading of Recommendations, Assessment, Development and Evaluation (GRADE) framework.^12^

To explore sources of heterogeneity in R estimates, we conducted subgroup analyses by estimation period, clade and type of R estimate (R_0_, R_e_ or R_t_). For heterogeneity in prevention percentages, we performed random-effects meta-regression (restricted maximum likelihood method) using three covariates: (1) extent of behaviour change, (2) first-dose vaccine coverage and (3) time delay (in weeks) between first reported case and vaccination rollout in the country or region (online supplemental text B).

### Patient and public involvement

Patients and/or the public were not involved in the design, conduct, reporting or dissemination plans of this research.

## Results

The search yielded 3323 complete MPXV genome sequences, 3943 potentially relevant records on R and 285 records on mitigation efforts as of 18 May 2025. We categorised MPXV genomes by clade, collection period and geographic region (online supplemental text A.1 and table A), and randomly selected 102 sequences using stratified sampling to construct a global phylogenetic tree spanning 1970–2025. After removing duplicates and ineligible records, 52 studies^13–64^ estimating mpox R and 40 modelling studies^4–6 32 33 43 48 49 51 52 54 56 59 61 65–90^ were included in the systematic review. Of these, 40 R estimates from 34 studies^13–19 21 22 25 26 28 31–34 37 39–41 43 45 47–51 53 54 59–61 63 64^ and 17 prevention effectiveness estimates from seven modelling studies^5 6 54 59 65 66 68^ met criteria for inclusion in the quantitative synthesis (see online supplemental figures A and B for the PRISMA flow diagram and online supplemental tables B–E listing reasons for exclusion). The 102 representative MPXV genome sequences included in the phylogenetic analysis were collected from 30 countries, spanning 4 low-income, 9 middle-income (4 lower-middle and 5 upper-middle) and 17 high-income economies. The extracted R estimates from 52 studies covered 21 countries—3 low-income, 5 upper middle-income and 13 high-income—together with three estimates at continental or global scale. In contrast, the 17 effectiveness estimates from empirical modelling studies were derived exclusively from four high-income countries, while data from low-income settings remain scarce.

### Global mpox phylogenetic tree, 1970–2025

**Figure 1:**
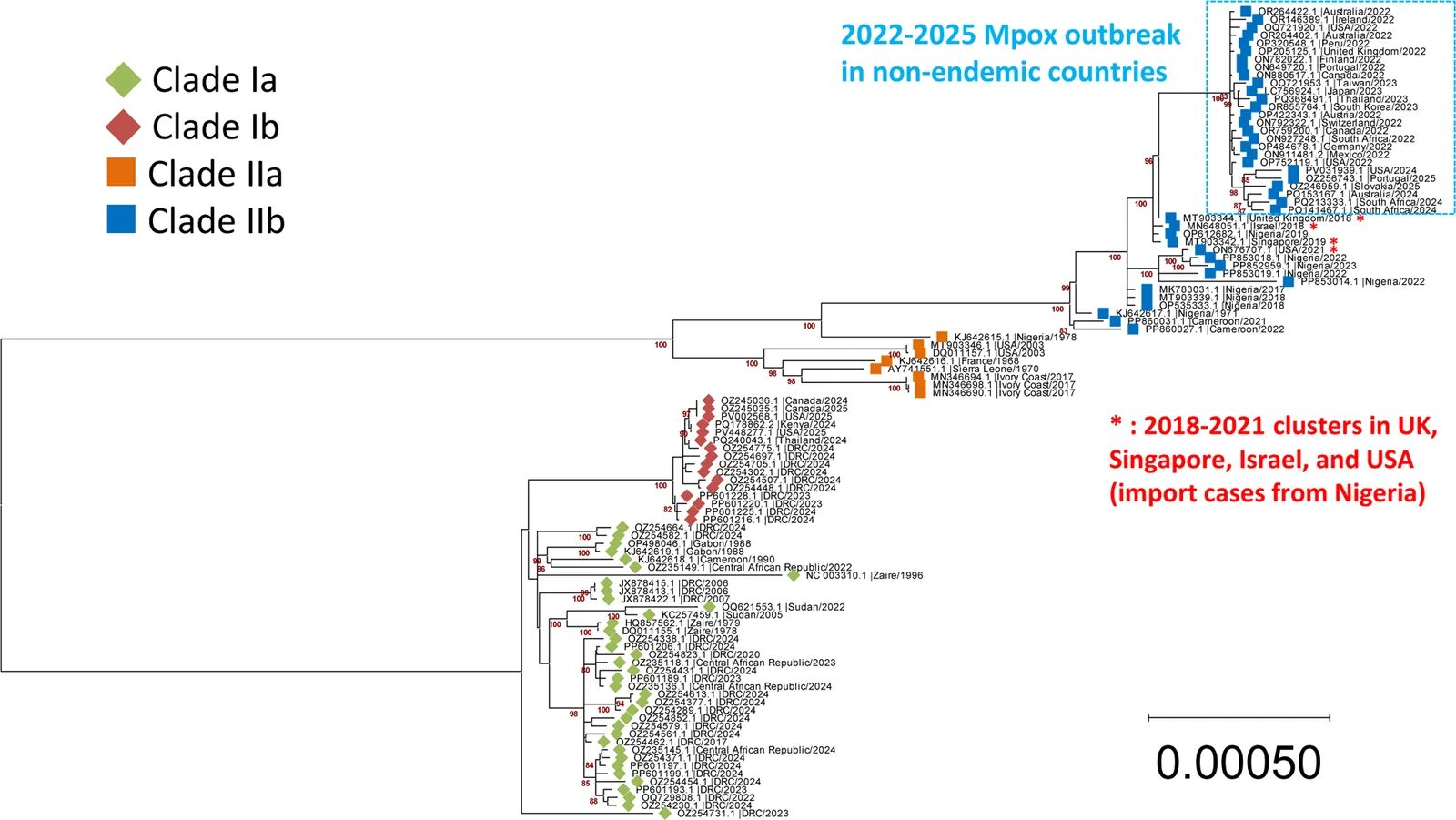
Phylogenetic tree of representative MPXV complete genome sequences. All 102 sequences were labelled according to their subclades. Bootstrap values (>80%) are shown below each branch. Sequences from the 2022–2025 clade IIb global outbreak in non-endemic countries are marked in cyan frame. Sequences from 2018 to 2021 clusters in UK, Singapore, Israel, and USA, associated with import cases from Nigeria, are labelled in red*. MPXV, mpox virus.

MPXV sequences from the 2022–2025 clade IIb outbreak in non-endemic countries (cyan frames) were most closely related to 2018–2019 isolates from the UK (MT903344.1), Singapore (MT903342.1), Israel (MN648051.1) and Nigeria (OP612682.1), the first three of which were linked to travel-related exportations from Nigeria in 2018–2019. These sequences could be further traced back to MK783031.1 from the 2017 Nigeria outbreak (figure 1). Additionally, a Nigerian isolate from 1971 (KJ642617.1) appeared on the basal branch of clade IIb, suggesting a zoonotic sister lineage predating the human-to-human transmission expansion.

Among clade I strains, subclade Ia showed high genetic diversity, with lineages accumulating across Central Africa since the 1970s. In contrast, sequences from the 2023–2025 DRC outbreak (South Kivu, Tshopo and Kinshasa) and travel-related cases in Kenya, Thailand, Canada and the US diverged from previous clade I (Ia) strains in Central Africa and formed a distinct cluster within a novel subclade Ib, first emerging in 2023 (figure 1).

Sensitivity analysis based on random resampling of MPXV sequences showed a similar overall clustering pattern in the phylogenetic tree (online supplemental figure Q).

### Increasing human-to-human transmissibility, 1970–2024

Among the 52 studies included in our review of mpox R, seven studies^13–19^ reported estimates before 2022 from African countries, mainly involving clade I and clade II transmission via zoonotic or limited human-to-human pathways. Forty-four studies^17 20–62^ estimated R during the 2022–2023 outbreaks across the Americas, Europe, Asia, Africa and Oceania, largely among high-risk MSM populations. Two studies^63 64^ analysed R during the 2023–2024 DRC outbreak involving clade Ib (see online supplemental table F for characteristics of all studies included in the review of mpox reproduction number). Narrative plots of all R estimates by clade and period are provided (online supplemental text D and figures C–G).

**Figure 2:**
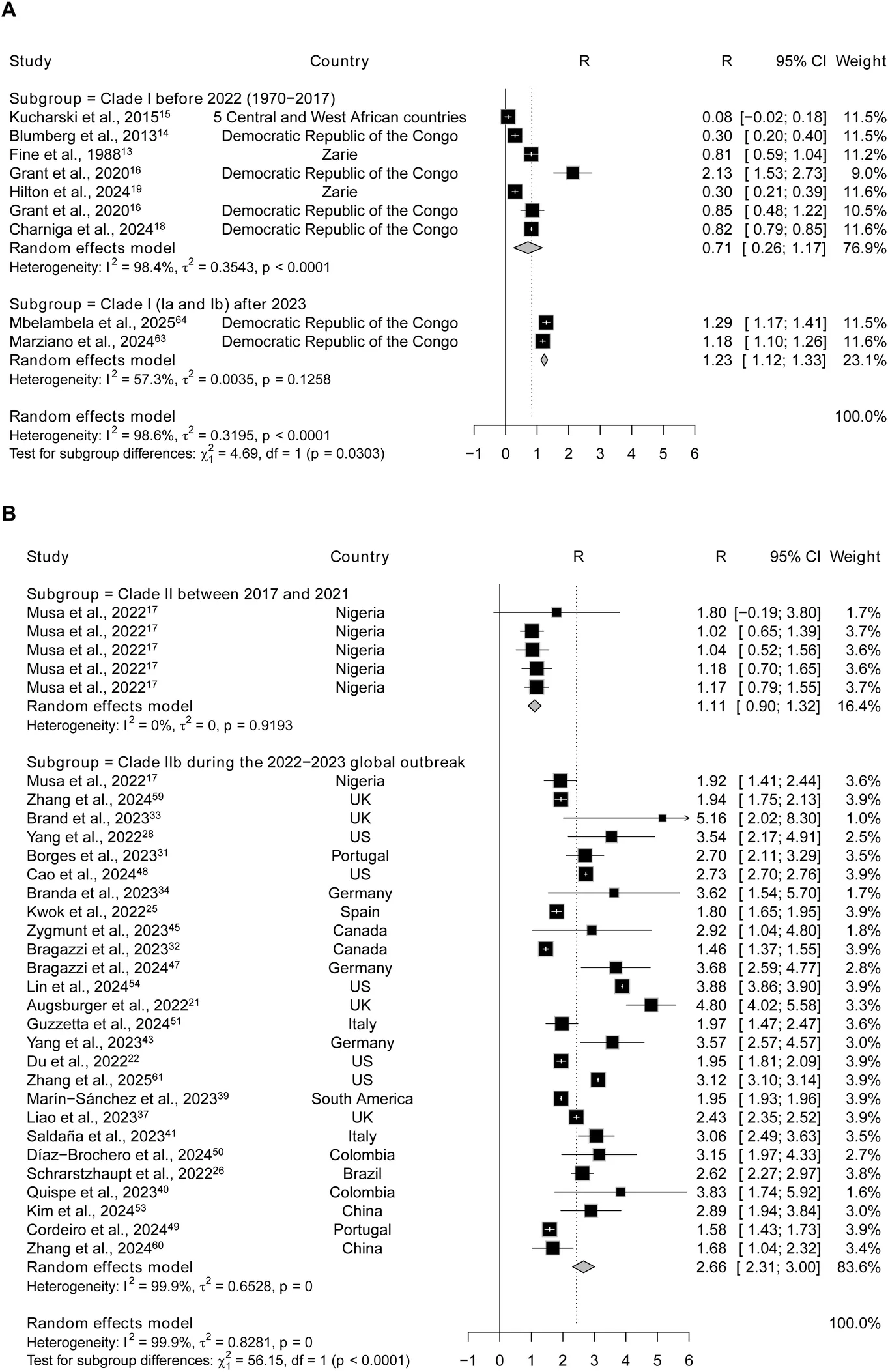
Forest plots of mpox reproduction number estimates for (**A**) clade I and (**B**) clade II, grouped by subclade and period of estimation. Studies are listed in the order of their periods of R estimation.

Of the R estimates included in the meta-analysis (n=40), 17 estimated R_0_, 9 peak initial R_t_ values (used as proxies for R_0_) and 14 mean or median R_t_ values (also known as R_e_). Random-effects meta-analyses of reproduction number estimates within clade I and clade II subgroups showed extremely high between-study heterogeneity (I²=98.6% and 99.9%, respectively) (figure 2A,B; see also online supplemental figure H). Funnel plot analyses and Egger’s tests (p=0.80 and p=0.52, respectively) did not reveal evidence of substantial publication bias (online supplemental figure I).

For clade I, pooled R estimates increased from 0.71 (95% CI 0.26 to 1.17) during 1970–2017 to 1.23 (1.12–1.33) for subclades Ib/Ia in the ongoing outbreak since 2023 (p=0.0303 for subgroup difference, figure 2A). For clade II, R rose from 1.11 (0.90–1.32) during 2017–2021 to 2.66 (2.31–3.00) for subclade IIb in the 2022–2023 global outbreak (p<0.0001 for subgroup difference, figure 2B).

### Evidence for impact of interventions in the 2022 global epidemic

Thirteen empirical modelling studies^5 6 33 51 54 59 61 65–70^ evaluated interventions during the 2022–2023 outbreak. The studied measures included behaviour change, vaccination and strategies such as contact tracing and isolation (see online supplemental table G and text E for the characteristics of empirical modelling studies). Seven of these studies^5 6 54 59 65 66 68^ reported prevention percentages and were included in the meta-analysis (see online supplemental table E for detailed reasons of modelling studies included in the review but not eligible for quantitative synthesis). The remaining 27 simulation-based studies^4 32 43 48 49 52 56 71–90^ (see online supplemental texts F and G for characteristics of the studies) explored optimal vaccine distribution strategies and the theoretical effects of pre-emptive vaccination and other public health interventions on the downturn of mpox cases. These included one model of a hypothetical mass vaccination before the DRC clade I outbreak beginning in 2023^84^ and another that simulated the spread of clade Ib in Asia.^89^

Most empirical models identified voluntary behaviour change—particularly reductions in sexual activity among MSM in response to health messaging—as a key factor in outbreak mitigation. In the UK, behaviour change alone prevented the majority of infections and substantially reduced R_t_.^33 59^ Similar findings were reported by models from Canada,^67^ Italy^51^ and the Netherlands.^70^ However, models from the USA^6 54^ and three Canadian cities^68^ suggested that vaccination eventually averted more cases, while behaviour change primarily delayed transmission, allowing time for vaccine rollout and immune response development.^6 54^ A narrative summary of these modelling findings is provided in online supplemental text E.

Despite evidence of moderate to high individual-level effectiveness of the modified Vaccinia Ankara-Bavarian Nordic vaccine,^91^ modelling studies yielded divergent estimates for their contribution to mpox outbreak control at the population level (online supplemental text E). In Canada and the USA, vaccination alone prevented an estimated 21%–39% of cases^54 68^; in Washington DC, as much as 79%.^6^ In contrast, only small impacts were estimated in the UK (averted an estimated 9.8% of cases)^59^ and Netherlands (7.1%).^5^

**Figure 3:**
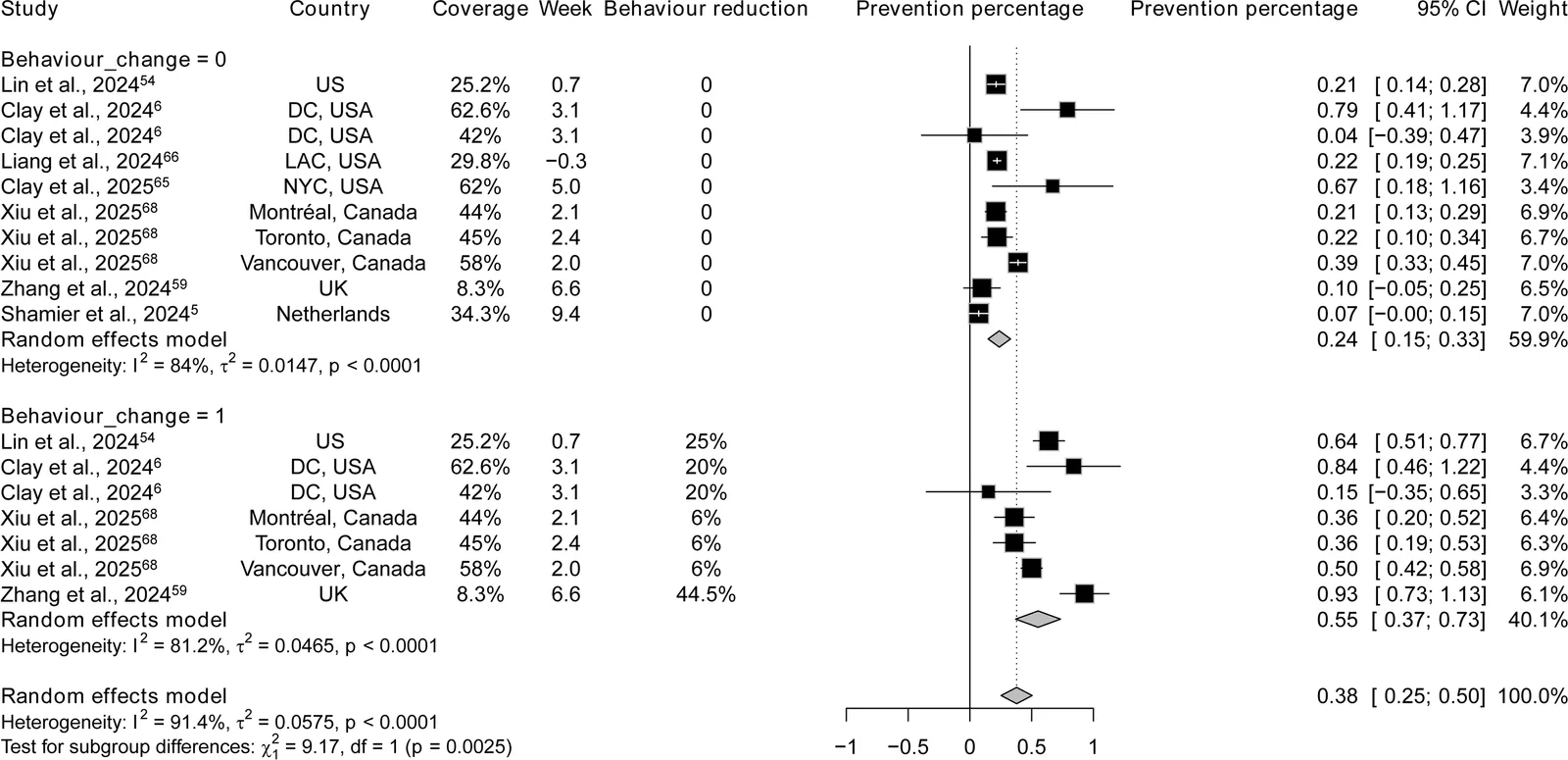
Forest plot of the estimates of prevention percentages for vaccination campaign (either with or without the incorporation of behaviour change), grouped by the incorporation of behaviour change. Prevention percentages are shown in decimal form (eg, 0.24=24%). The ‘Coverage’ column indicates the first dose vaccine coverage of the model population by the time for estimating prevention percentages. The ‘Week’ column indicates the number of weeks from the first case confirmation to the initiation of vaccination rollout. The ‘Behaviour reduction’ column indicates the extent of behaviour change, quantified as the proportional reduction in sexual contact rates, transmission probability per sexual contact, or number of sexual partners, relative to preoutbreak levels. DC, Washington, District of Columbia; LAC, Los Angeles County; NYC, New York City.

**Figure 4:**
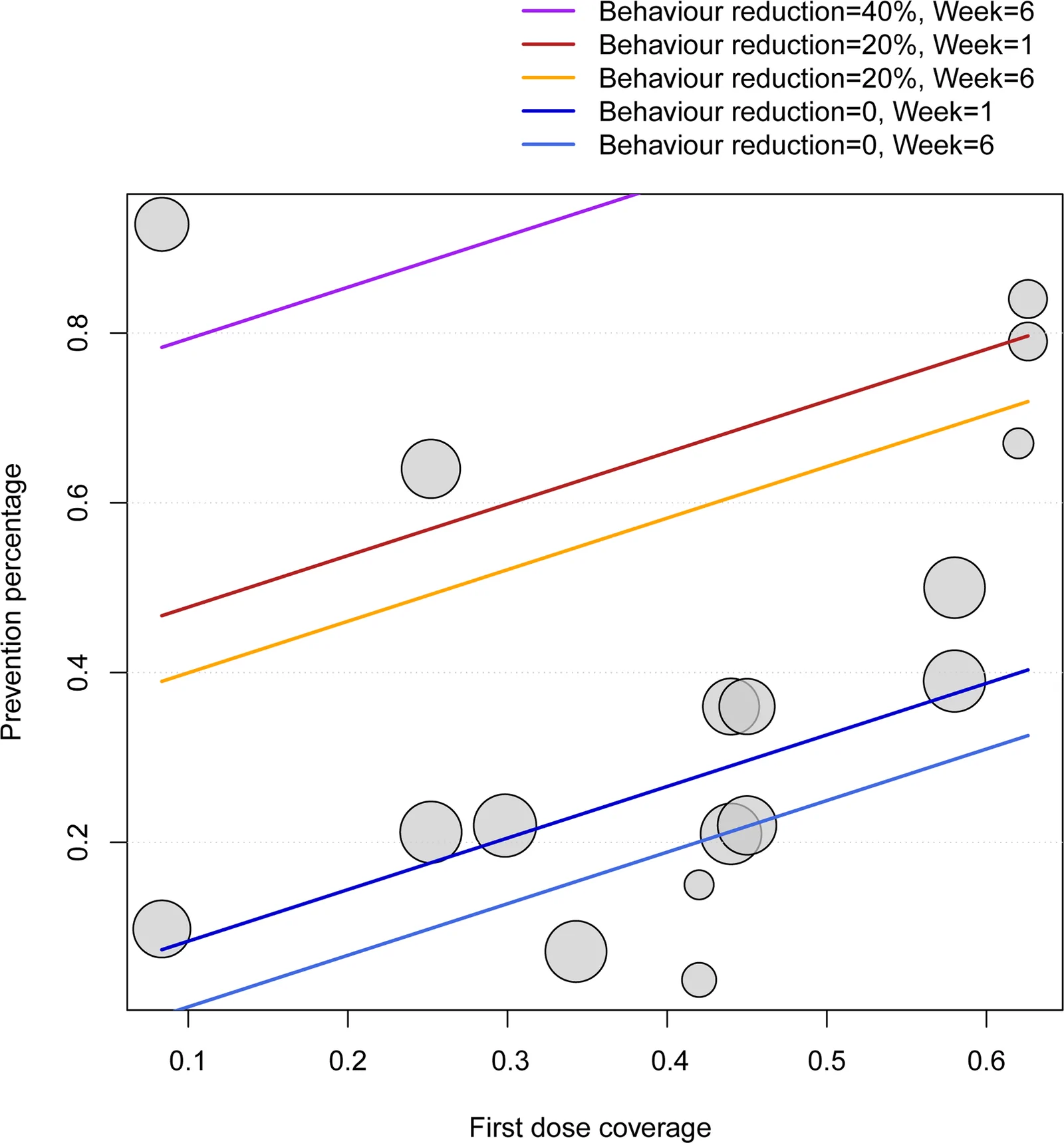
Meta-regression of prevention percentages for vaccination campaign, by first-dose coverage, weeks from first case confirmation to vaccination initiation and extent of behaviour reduction. The x-axis represents first-dose mpox vaccine coverage. Coloured lines represent different combinations of behaviour reduction (0, 20%, 40%) and weeks from first case confirmation to vaccination initiation (1 week, 6 weeks). Meta-regression identified first-dose coverage (coefficient: 0.61, SE: 0.094, 95% CI 0.42 to 0.79), weeks from first case confirmation to vaccination initiation (coefficient: −0.016, SE: 0.0043, 95% CI −0.024 to −0.0070) and extent of behaviour reduction (coefficient: 1.97, SE: 0.17, 95% CI 1.63 to 2.31) as significant predictors in this model. The model accounted for 99.8% of the between-study heterogeneity (R^2^=99.8%).

The pooled estimate from 17 vaccination-associated prevention percentages was 38% (95% CI 25% to 50%; I^2^=91.4%) (figure 3). During the 2022 global epidemic, behaviour change combined with vaccination campaigns prevented 55% (95% CI 37 to 73; I^²^=81.2%) of cases compared with counterfactual scenarios without interventions, whereas vaccination alone would have prevented 24% (95% CI 15 to 33; I²=84.0%) (p=0.003; figure 3). However, effect sizes varied across settings. Meta-regression suggested that heterogeneity was associated with the extent of behaviour change, the timing of vaccine rollout and first-dose coverage (R^2^=99.8%; figure 4). High first-dose coverage, early initiation of vaccination and greater extent of behaviour change were associated with a larger proportion of mpox cases prevented. The funnel plot demonstrates asymmetry suggestive of potential publication bias, although Egger’s test does not reach statistical significance (p=0.087; online supplemental figure J).

A few studies examined the effectiveness of contact tracing and isolation, although the accuracy of these estimates could be affected by whether the model structures in the studies accounted for presymptomatic and asymptomatic transmission. In the UK, reducing the infectious period by 20% through earlier diagnosis and isolation prevented an estimated 47.4% of infections.^59^ Contact tracing at 20% coverage of diagnosed cases averted 14% of cases in Canada,^68^ and approximately 50% in Italy compared with no tracing,^51^ with higher tracing coverage, potentially leading to a greater probability of stochastic extinction.^51^

### Susceptible depletion and the 2022 epidemic decline

Murayama *et al*^4^ constructed mpox dynamical models in 30 countries incorporating heavy-tailed sexual networks (ie, assuming a very small but high-risk group) among MSM, and suggested that, theoretically, the depletion of susceptible individuals (after infection-derived immunity) alone, in the absence of intervention effects, can explain the observed mpox epidemic peak and decline.

Empirical modelling studies examined this hypothesis. Xiridou *et al*^70^ modelled the scenario without behaviour adaptation and when vaccination programme had not yet started, in which the simulated daily mpox cases peaked and began to decline around 8 July 2022, similar to what had been observed in the Netherlands. However, this no-behaviour-adaptation scenario had a less steep decline trajectory compared with real-world data, indicating that factors beyond the depletion of susceptible individuals, namely, public health interventions, contributed to the rapid drop in mpox cases in the Netherlands. On the other hand, Clay *et al*^6^ and Brand *et al*^33^ fit their models to the observed mpox epidemic trend under hypothetical scenarios without behaviour adaptation and/or vaccination in the USA and the UK, respectively. The former found a worse fit under the susceptible-depletion-alone scenario, compared with the main scenario with control effects from behaviour change and vaccination.^6^ The latter also observed that the model with no effects from behaviour change exhibited worse performance in forecasting mpox incidence.^33^ Collectively, these modelling results serve as additional evidence for the impact of public health measures in the decline of the 2022 global mpox epidemic, while depletion of susceptible individuals in a relatively small but high-risk group following infection-derived immunity also contributed to the downturn of mpox cases.^4^

### Role of targeted vaccination in preventing resurgence

Several studies modelled mpox resurgence risk in Europe and North America under varied circumstances (online supplemental text G). Targeted vaccination of high-risk groups was consistently identified as critical for preventing future waves. In the UK, models suggested that when sexual activity of the high-risk group rebounded to preoutbreak levels by late 2022, random vaccination of sexually active MSM led to resurgences,^59^ whereas prioritising highly sexually active individuals averted further transmission.^33 59^ In the Netherlands, vaccination and immunity from prior infection were estimated to reduce future outbreak size by 74% compared with the 2022–2023 outbreak.^5^ In contrast, infection-induced immunity alone (ie, without vaccination) would reduce a future outbreak by only an estimated 16.6%.^5^ In Italy, vaccinating all MSM with more than 10 sexual contacts per year (23% of MSM) in combination with 20% contact tracing coverage was estimated to be necessary to reduce R_t_ below 1 and prevent potential resurgences.^51^

### Risk of bias assessment and certainty of the evidence

Risk of bias assessment for the included studies is presented in online supplemental text H and figures K–M and table H. Studies estimating R after 2022 and empirical models had the highest average scores (0.67), compared with pre-2022 R estimates (0.59) and theoretical models (0.55). The most common sources of bias were insufficient justification of modelling assumptions and methodological choices.

Leave-one-out sensitivity analyses showed that no individual study had a substantial influence on the pooled estimates of R or prevention percentages (online supplemental figures N and O). The certainty of evidence was high for the combination of vaccination campaigns and behaviour change to control the mpox outbreak and moderate for the effectiveness of vaccination campaigns alone (online supplemental table I).

## Discussion

To our knowledge, this is the first systematic review and meta-analysis to characterise longitudinal changes in mpox transmissibility in a phylogenetic context and assess the effectiveness of vaccination for outbreak control. We found stepwise increases in human-to-human transmissibility within each clade, associated with the emergence of specific subclades in the context of ongoing viral evolution, culminating in the highly transmissible subclade IIb that fuelled the 2022 global outbreak and the subclades Ib/Ia that are driving the ongoing epidemic in Africa. Our meta-analysis further suggests that behaviour change and vaccination campaigns likely played important roles in the decline of the 2022 mpox epidemic in many studied settings. Empirical modelling studies estimated substantial reductions in cases associated with these interventions, although the magnitude of effect varied across contexts. Between-study heterogeneity was associated with differences in the extent of behaviour change as well as the timing and first-dose coverage of vaccine rollout. Taken together, these findings help reconcile previously divergent interpretations of the 2022 global mpox outbreak and support a context-specific understanding of its decline.

Our systematic review and meta-analysis of empirical modelling studies suggests that early implementation of vaccination campaigns together with behaviour change, when achieved at sufficient coverage, is likely to be effective in controlling mpox outbreaks (figure 4; online supplemental figure P). Previous work by Xiu *et al*^68^ highlighted the importance of vaccination timing and coverage, showing that standardising these two factors across three Canadian cities yielded identical estimates of the cumulative fraction of new infections averted by vaccination. Most modelling studies also found that sexual behaviour adaptation among MSM towards lower risk practices contributed substantially to reductions in mpox R, more rapid declines in epidemic curves and fewer cases.^5 33 51 59 67 70^ However, only a few studies from the US explicitly evaluated the synergistic effect of combining vaccination and behaviour change.^6 54^ These studies suggested that behaviour change may slow transmission and flatten the epidemic curve, thereby allowing time for vaccination to confer more durable protection and resulting in greater case reduction overall.^6 54^ Importantly, although we present pooled estimates from counterfactual vaccination-alone scenarios, these should be interpreted cautiously, because in real-world settings, behaviour change and vaccination were often implemented concurrently and may have acted synergistically. Accordingly, the independent effect of vaccination cannot be cleanly disentangled from that of behaviour change in many settings.

The observed increases in mpox transmissibility, particularly for clade I in Africa, should be interpreted in light of the accumulation of susceptible individuals and declining herd immunity following the cessation of smallpox vaccination since 1980, together with ecological changes such as deforestation that may facilitate zoonotic reintroductions and increase contact between humans and animal reservoirs, creating opportunities for subsequent human-to-human transmission.^92^ In addition, introduction of the virus into populations with high-contact sexual networks has played an important role in amplifying transmission in recent outbreaks.^93^ Available molecular and phylogenetic data are also consistent with a possible contributing role for viral evolution, although direct evidence that viral adaptation was a primary driver of increased human-to-human transmission remains limited. Previous studies have shown that clade IIb strains from the 2022–2023 outbreak clustered closely with post-2017 Nigerian isolates and travel-related sequences detected in the USA, UK, Singapore and Israel during 2018–2021.^42 94^ The accelerated mutation rate and excess GA-to-AA and TC-to-TT substitutions observed in clade IIb MPXV, compared with earlier clades Ia and IIa and with other orthopoxviruses, have been suggested to reflect host apolipoprotein B mRNA-editing enzyme, catalytic polypeptide 3 (APOBEC3)-mediated editing associated with sustained human transmission.^94^ The 1971 Nigerian isolate KJ642617.1 may represent a zoonotic sister lineage predating the emergence of APOBEC3-associated mutational signatures, which may have accompanied sustained human-to-human transmission beginning around 2016.^95^ Genomic studies from the DRC have also demonstrated APOBEC3 enrichment in newly emerged clade Ib sequences.^96 97^ In a 2024 phylogenomic study from Kinshasa, similar proportions of APOBEC3-type mutations were observed in co-circulating clade Ia (68%) and Ib (72%) isolates.^97^ Our pooled R estimate for clade I subclades Ia/Ib (1.23) is consistent with these observations, but should be interpreted cautiously and not as evidence that viral evolution alone explains the observed increase in transmissibility. Continued genomic and epidemiological surveillance is, therefore, essential to clarify the relative contributions of viral evolution, population susceptibility, ecological change and network-driven transmission to future mpox outbreaks.

Some uncertainty remains regarding the R estimates assigned to clade II during 2017–2021, as the original Nigerian study did not explicitly specify the clade corresponding to its reported estimates. In our meta-analysis, these estimates were classified as clade II based on the best available phylogenetic and epidemiological evidence, including genomic data, indicating that outbreak strains in Nigeria from 2017 onwards were predominantly assigned to clade IIb lineage A.^94^ Using a renewal equation-based method, the original study reported annual R estimates in a setting where repeated zoonotic spillovers and human-to-human transmission co-occurred. If zoonotic reintroductions of mpox were not fully distinguished from secondary human-to-human transmission, the reported R estimates may have been overestimated, thereby attenuating the observed difference in transmissibility between the 2017–2021 and 2022–2023 periods within clade II.

This study had several important limitations. First, diagnostic complexities may reduce case ascertainment, particularly in resource-limited settings.^98^ Second, primary studies varied in the methods and assumptions used to estimate R and intervention effectiveness. For instance, R estimates based on renewal equations rely on generation time or serial interval distributions derived from relatively few infector–infectee pairs identified through contact tracing, which may themselves be limited by the quality of contact reports due to disease-related stigmatisation and the intensity of tracing efforts. To ensure scientific rigour, we included only 40 R estimates considered indicative of the intrinsic transmissibility of MPXV clades with lower risk of bias (online supplemental text A.2) in the meta-analysis of R estimates. Regarding mathematical model structure, parsimonious assumptions of homogeneous random mixing within MSM populations may underestimate R values, whereas model structures that do not account for presymptomatic or asymptomatic transmission are more likely to overestimate the effects of early diagnosis, isolation and contact tracing. To minimise such biases, we prioritised risk-structured model-based R estimates during data extraction and focused our meta-analysis on the likely impacts of vaccination campaigns and behaviour change, as estimates for these interventions were less likely to be influenced by differences in model structure across primary studies. In some studies, the extent of sexual risk reduction following awareness of mpox outbreaks among at-risk populations may not have been accurately estimated solely through calibration to epidemic curves, potentially affecting the reliability of the estimated effects of behaviour change. We note that two US modelling studies^48 59^ used empirical behaviour survey data or social media activity data to support parameterisation of behaviour adaptation; both studies demonstrated synergistic effects between behaviour change and vaccination campaigns. In addition, we recognise that restricting eligibility to peer-reviewed publications may have led to the exclusion of some relevant grey literature. However, unlike individual-level outcomes such as treatment response or the proportion of infection or severe disease following vaccination, R estimates and prevention percentages are population-level measures that require substantial methodological rigour in their estimation. We, therefore, adopted this approach to ensure greater credibility and quality of the included evidence. Finally, our study identified the scarcity of empirical modelling studies on vaccination impact in low-income and middle-income settings, probably reflecting substantial delays in initiating vaccination campaigns under resource constraints, as an important research gap. This highlights the pressing need for global investment to improve access to mpox vaccines in these regions.

The ongoing mpox crisis in Africa, declared a PHEIC by WHO on 14 August 2024,^7^ represents a complex public health challenge. Characterised by geographic expansion beyond the traditional endemic regions, involvement of multiple clades and mixed modes of transmission including sexual, household contact and zoonotic pathways, current spread has had a disproportionate impact on children under the age of 15.^2^ Although the PHEIC was lifted on 5 September 2025,^99^ mpox persists as a continental emergency, with the Africa Centres for Disease Control and Prevention (Africa CDC) maintaining a PHECS at high alert^100^ and recommending continued surveillance, vaccine supply and community engagement. The findings from this systematic review and meta-analysis provide robust evidence for the merits of early and extensive mpox vaccination rollout targeting at-risk populations in affected countries. Furthermore, our results indicate that risk-reducing behaviour change may be a critical component in control strategies. By vaccinating the most at-risk populations as early as possible and simultaneously implementing risk communication to reduce contact transmission rates (both human-to-human and zoonotic), sufficient time could be created for immunisation to occur before exposure. Given the complex epidemiology of mixed clade outbreaks in the DRC and surrounding countries, future modelling efforts are essential to better understand evolving transmission dynamics across regions and to guide optimisation of intervention strategies for containing each outbreak.

## Conclusion

In conclusion, this systematic review and meta-analysis found that behaviour change and vaccination likely played important roles in the decline of the 2022 mpox epidemic in many studied settings. Empirical modelling studies estimated substantial reductions in cases associated with these interventions, although effect sizes varied across settings according to vaccine rollout timing, coverage and the extent of behaviour change. As the ongoing viral evolution of MPXV has been accompanied by increasing human-to-human transmissibility and spread into previously unaffected populations, effective epidemic control will require proactive, rapid and equitable vaccine rollout, supplemented by culturally tailored risk communication. The near-absence of empirical data from low and middle-income countries highlights a critical equity gap: delayed vaccine access not only undermines outbreak control but also weakens the evidence base needed to guide global responses. Addressing this gap through timely vaccine allocation and sustained investment in surveillance and research across diverse settings is essential both for controlling the ongoing epidemic in Africa and for preventing future global mpox outbreaks.

## Supplementary material

10.1136/bmjgh-2025-022252 online supplemental file 1

## Data Availability

All data relevant to the study are included in the article or uploaded as supplementary information.
