# Supplementary material for: Dynamics of global mpox epidemics, 1970–2025: a systematic review and meta-analysis of evolving transmissibility and intervention effects
Source: BMJ Glob Health. 2026 Aug 20;11(8):e022252. doi: 10.1136/bmjgh-2025-022252 (PMC13504931; doi:10.1136/bmjgh-2025-022252)
Supplement: Supplementary file 1 [file bmjgh-11-8-s001.pdf]

Supplemental material

Supplement to:

Dynamics of global mpox epidemics, 1970–2025: A systematic review and meta-analysis of evolving transmissibility and intervention effects

Yin-Chien Lin, Tzai-Hung Wen, Wei-Liang Shih, Chia-Wei Liao, Sten H. Vermund, Chi-Tai Fang

Content:

Supplemental Method:

Text A. Method: Search strategies, selection criteria, and data extraction ··········· 4
A.1 MPXV sequences
A.2 Mpox reproduction number
A.3 Modelling studies on the effects of control measures during the 2022-2023 mpox global outbreak
Text B. Method: Meta-analysis ··········· 9
B.1 Mpox reproduction number
B.2 Prevention percentages for vaccination (either with or without the incorporation of behaviour change)
B.3 Meta-regression
Text C. Method: Risk of bias assessment ··········· 10

Supplemental Results:

Text D. Results: Description of the mpox reproduction number ··········· 12
D.1 Overview of the mpox reproduction number estimates
D.2 R of clade Ia mpox in the DRC between 1970 and 2017
D.3 R of clade II mpox in Nigeria between 2017 and 2022
D.4 R of clade IIb mpox during the 2022-2023 global outbreak
D.5 R of clade Ia and Ib mpox in the DRC after 2023
Text E. Results: Narrative review of the effects for control measures in empirical modellings··········· 13
E.1 Behaviour adaptations
E.2 Vaccination

Text F. Results: Theoretical simulations of vaccine allocation and public health strategies in the 2022-2023 mpox clade IIb global outbreak and clade I outbreaks after 2023 ······ 16
F.1 Modelling studies targeting the 2022-2023 global outbreak
F.2 Modelling studies targeting the DRC clade I outbreak since 2023 and importation-triggered clade Ib outbreaks in Asia
Text G. Results: Possibilities of mpox resurgences under various conditions ······ 17
Text H. Results: Risk of bias assessment ······ 18

Reference ······ 72

Supplemental Figures

Figure A. PRISMA flow diagram for the review on estimates of mpox reproduction number ······ 19
Figure B. PRISMA flow diagram for the review on the effects of mpox mitigation measures assessed by transmission dynamic model ······ 20
Figure C. Time series plot of mpox R estimates (R_0, R_t or R_e) and their 95% confidence or credible intervals (interquartile range shown when 95% CI or CrI weren't available) from studies included in the meta-analysis ······ 21
Figure D. Time series plot of the reproduction number estimates of mpox clade I between 1970 and 2017, from all the included studies ······ 22
Figure E. Time series plot of the reproduction number estimates of mpox clade II before 2022, from all the included studies ······ 23
Figure F. Time series plot of the reproduction number estimates of mpox clade IIb after 2022, from all the included studies ······ 25
Figure G. Time series plot of the reproduction number estimates of mpox clade I (Ia + Ib) after 2023, from all the included studies ······ 26
Figure H. Forest plot of mpox reproduction number estimates for (A) clade I and (B) clade II, grouped by period of estimation and type of reproduction number estimated ······ 27
Figure I. Funnel plot of R estimates included in the meta-analysis of mpox reproduction numbers (A) clade I and (B) clade II, with pseudo 95% confidence limits ······ 28
Figure J. Funnel plot of prevention percentages included in the meta-analysis, with pseudo 95% confidence limits ······ 29
Figure K. Trends in risk of bias assessment scores by publication year ······ 30
Figure L. Boxplot of risk of bias assessment scores, by study type ······ 31
Figure M. Proportion of studies rated Yes, No, or NA for each of the seven risk-of-bias assessment items ·· 32
Figure N. Leave-one-out forest plot of mpox R estimates ······ 33
Figure O. Leave-one-out forest plot of prevention percentages ······ 35
Figure P. Meta-regression of prevention percentages for vaccination only, by first-dose coverage and weeks

from first case confirmation to vaccination initiation ········ 36
Figure Q. Sensitivity phylogenetic tree generated from an independent repeat of the same stratified sampling framework as the main analysis ········ 37

Supplemental Tables

Table A. Sequences included in phylogenetic tree construction ········ 38
Table B. Information on studies excluded by full-text reading, for the review on estimates of mpox reproduction number ········ 41
Table C. Information on studies excluded by full-text reading, for the review on the effects of mpox mitigation measures assessed by transmission dynamic model ········ 47
Table D. Information on studies included in the review of mpox reproduction number, but not eligible for quantitative synthesis ········ 49
Table E. Information on studies included in the review of the effects of mpox mitigation measures assessed by transmission dynamic model, but not eligible for quantitative synthesis ········ 50
Table F. Data extraction form for papers of the reproduction number estimates ········ 52
Table G. Dynamic modelling studies of mpox evaluating the impact of mitigation measures implemented in each country or region during the 2022–2023 mpox outbreak ········ 55
Table H. Risk of bias (quality) assessment score of the included studies, by study type ········ 67
Table I. Certainty of evidence for effectiveness of vaccination campaigns without or with behaviour change ········ 71

Supplemental Method

Text A. Method: Search strategies, selection criteria, and data extraction

A.1 MPXV sequences

We retrieved all available MPXV full genome sequences from GenBank as of 18 May, 2025. We categorised sequences into strata based on the following order: clades, subclades, periods of collection (before or after the 2022 mpox global outbreak and the 2023-2025 mpox outbreak in the Democratic Republic of the Congo (DRC)), and geographical regions of collection (from countries endemic to clade I or II mpox, or from non-endemic countries).

Given the large number of publicly available sequences (n=3,323) and the objective of the phylogenetic analysis, we applied a stratified sampling approach to construct a representative dataset for phylogenetic tree construction. The aim of this analysis was to illustrate the broad evolutionary patterns of mpox across clades, subclades, and time periods, ensuring clarity and interpretability of the phylogenetic tree, and to contextualise estimates of transmissibility (R) within their corresponding phylogenetic and epidemiological settings.

Sampling was therefore conducted within each stratum to ensure coverage across major epidemiological categories while maintaining interpretability of the phylogenetic tree and avoiding over-representation of strata with large numbers of sequences (e.g., clade IIb sequences from the 2022–2023 global outbreak). For strata with a large number of sequences from multiple countries, a two-stage sampling strategy was applied: countries were first randomly selected, followed by random selection of one or more sequences within each selected country.

Of the 3,323 MPXV full genome sequences retrieved from GenBank, 644 sequences belong to clade Ia (40 collected before 2022), 173 belong to clade Ib (all collected after 2023), 23 belong to clade IIa (all collected before 2022), and 2,483 belong to clade IIb (46 collected before 2022). We randomly sampled sequences from each category to build the final dataset of 102 representative sequences for the phylogenetic tree analysis, to ensure both clarity and comprehensiveness. The detail of MPXV sequences in our phylogenetic tree are listed in supplemental table A. The detailed categorisation and sampling process are described below:

1. Clade Ia: 644 full genome sequences retrieved from GenBank (40 collected before 2022, 8 collected in 2022, 9 collected in 2023, and 587 collected in 2024), 37 sequences included in the phylogenetic tree analysis
   1.1 Sequences collected before 2022 (12 sequences included):
   Among the 40 sequences collected before 2022, the geographical regions of collection included only those endemic to mpox, namely Zaire, the Democratic Republic of the Congo, Cameroon, Sudan, and Gabon. 12 sequences were randomly sampled from the 40 sequences to be included in the

phylogenetic tree analysis.

1.2 Sequences in 2022 (3 sequences included):

Among the 8 sequences collected in 2022, the geographical regions of collection included only those endemic to mpox, namely the Democratic Republic of the Congo, the Central African Republic, and Sudan. 3 sequences were randomly sampled from the 8 sequences to be included in the phylogenetic tree analysis.

1.3 Sequences in 2023 (4 sequences included):

Among the 9 sequences collected in 2023, the geographical regions of collection included only those endemic to mpox, namely the Democratic Republic of the Congo, and the Central African Republic. 4 sequences were randomly sampled from the 9 sequences to be included in the phylogenetic tree analysis.

1.4 Sequences in 2024 (18 sequences included):

Among the 587 sequences collected in 2024, 34 were collected in the Central African Republic, and the remaining 553 in the Democratic Republic of the Congo. 3 sequences collected in the Central African Republic and 15 in the Democratic Republic of the Congo were randomly sampled from the 34 and 553 available sequences, respectively, to be included in the phylogenetic tree analysis.

2. Clade Ib: 173 full genome sequences retrieved from GenBank (6 collected in 2023, 162 collected in 2024, and 5 collected in 2025), 16 sequences included in the phylogenetic tree analysis

2.1 Sequences in 2023 (2 sequences included):

Among the 6 sequences collected in 2023, all of them were collected in the Democratic Republic of the Congo. 2 sequences were randomly sampled from the 6 sequences to be included in the phylogenetic tree analysis.

2.2 Sequences in 2024 (11 sequences included):

Among the 162 sequences collected in 2024, 157 were collected in the Democratic Republic of the Congo, and 5 sequences from 3 countries non-endemic to mpox (Thailand, Kenya, and Canada). 8 sequences collected in the Democratic Republic of the Congo and 3 in non-endemic countries were randomly sampled from the 157 and 5 available sequences, respectively, to be included in the phylogenetic tree analysis.

2.3 Sequences in 2025 (3 sequences included):

Among the 5 sequences collected in 2025, all were collected in countries non-endemic to mpox (Thailand, USA, and Canada). 3 sequences were randomly sampled from the 5 available sequences to be included in the phylogenetic tree analysis.

3. Clade IIa: 23 full genome sequences retrieved from GenBank (all collected before 2022), 8 sequences included in the phylogenetic tree analysis

3.1 Sequences before 2022 (8 sequences included):

Among the 23 sequences collected before 2022, 14 were collected in countries endemic to mpox

(Sierra Leone, Liberia, Nigeria, and Cote d'Ivoire), the remaining 9 from countries non-endemic to mpox (the US, Denmark, Netherlands, and France; the majority of sequences were collected from non-human primates, only sequences in the US includes those collected from human). 5 sequences collected in endemic countries and 3 in non-endemic countries were randomly sampled from the 14 and 9 available sequences, respectively, to be included in the phylogenetic tree analysis.

4. Clade IIb: 2483 full genome sequences retrieved from GenBank (46 collected before 2022, 2317 collected between 2022 and 2023, 120 collected between 2024 and 2025), 41 sequences included in the phylogenetic tree analysis
   4.1 Sequences collected before 2022 (10 sequences included):
   Among the 46 sequences collected before 2022, 39 were collected in countries endemic to mpox (Nigeria [37], and Cameroon [2]), the remaining 7 from countries non-endemic to mpox (the US, Singapore, Israel, and the UK). 5 sequences collected in Nigeria, 1 sequence collected in Cameroon and 4 in non-endemic countries were randomly sampled from the 39, 2, and 7 available sequences, respectively, to be included in the phylogenetic tree analysis.
   4.2 Sequences collected between 2022 and 2023 (25 sequences included):
   Among the 2317 sequences collected between 2022 and 2023, 93 were collected in countries endemic to mpox (Nigeria [86], and Cameroon [7]), the remaining 2224 from 32 countries (Europe [979 sequences from 16 countries], the Americas [1102 sequences from 6 countries], Asia [112 sequences from 7 countries], Africa [3 sequences from Egypt and South Africa], and Oceania [28 sequences from Australia]) non-endemic to mpox. For sequences from the endemic countries, 4 sequences collected in Nigeria and 1 sequence collected in Cameroon were randomly sampled from the 86 and 7 available sequences, respectively, to be included in the phylogenetic tree analysis. For sequences from the non-endemic countries, we first randomly sampled 7 countries from the 16 countries in Europe, 6 countries from the 6 countries in the Americas, 4 countries from the 7 countries in Asia, 1 country from the 2 countries in Africa. Then within each selected country, one sequence was randomly sampled, plus a total of 2 sequences randomly sampled from the 28 sequences in Australia, to be included in the phylogenetic tree analysis.
   4.3 Sequences collected between 2024 and 2025 (6 sequences included):
   Among the 120 sequences collected between 2024 and 2025, the geographical regions of collection included only those non-endemic to mpox (8 countries), namely Australia, China, Japan, South Africa, Ireland, Portugal, Slovakia, and the US. We first randomly selected 6 countries from the 8 countries. Then within each selected country, one sequence was randomly sampled to be included in the phylogenetic tree analysis.

An independent repeat of the same stratified sampling framework was conducted to generate an alternative phylogenetic tree for sensitivity analysis, which yielded consistent overall clustering patterns and clade assignments (supplemental figure Q).

A.2 Mpox reproduction number

Search terms:
("mpox" OR "monkeypox" OR "MPXV" OR "monkey pox") AND ("reproduction number*" OR "reproductive number*" OR "reproduction ratio*" OR "reproductive ratio*" OR "transmission" OR "transmissibility")

We searched PubMed and Embase for studies that assessed the transmissibility of mpox and reported mpox reproduction number (R) estimates over defined time intervals, published until May 18, 2025. Eligible studies estimated the reproduction number of mpox (basic reproduction number (R_0), effective reproduction number (R_e), time-dependent reproduction number (R_t), or R unspecified) in human populations within specified countries/regions during a defined period, using methods such as data-driven approaches, renewal-equation-based methods, and mathematical modelling. There were no restrictions on the language of the articles. We excluded studies that estimated reproduction numbers in hypothetical settings or non-human populations, animal challenge models, estimations of within-host reproduction numbers, studies that presented estimates of mpox reproduction number in plots but did not report numerical values, or those not peer-reviewed. Cross-checking of references was performed to identify additional articles that might be relevant.

The extracted information for included studies reporting reproduction numbers are as follows: title, authors, published year, study design for R estimation, materials and settings, time period of the R estimates, type of R outcome reported (R_0, R_e, or R_t) and its value with uncertainty intervals. When available, we extracted R_0 or the maximum R_t (R_e) for the initial period of the epidemic from each study, which serves as a proxy for R_0; otherwise, the mean or median R_t (R_e) of the estimation period was extracted. For studies that employed both homogeneous and risk-structured mixing assumptions in their modellings, we prioritised R estimates from risk-structured models for estimates of the 2022–2023 outbreak. For studies that estimated R in more than one country during the 2022-2023 mpox outbreak, we extracted the R estimates of the country with the highest R values from each individual study.

For the quantitative synthesis, the meta-analysis comprised: (1) all R estimates for clade I and clade II outbreaks prior to 2022; (2) R estimates for clade IIb during 2022 that indicate the intrinsic transmissibility of the new clade to cause outbreak and met all of the following epidemiological criteria; (3) All R estimates for clade IIb during 2023–2024; and (4) all R estimates for clade Ib/Ia outbreaks between 2023 and 2025.

R estimates for clade IIb during 2022 were included only if the corresponding studies met all of the following epidemiological criteria:

(1) the cumulative number of cases during the R estimation period exceeded 400;
(2) the estimation period encompassed both the initial and ascending phases of the outbreak;
(3) the study received a risk-of-bias (quality) assessment score greater than 0.5.

The PRISMA flow diagram (supplemental figure A) presents both the overall inclusion of studies in the review and those included in the quantitative synthesis.

A.3 Modelling studies on the effects of control measures during the 2022-2023 mpox global outbreak

Search terms:
("mpox" OR "monkeypox" OR "MPXV" OR "monkey pox") AND ("vaccination*" OR "vaccine*" OR "immuniz*" OR "immunis*" OR "aware*" OR "behavior*" OR "behaviour*" OR "adapt*" OR "change*") AND ("men who have sex with men" OR "MSM" OR "high-risk" OR "high risk" OR "at-risk" OR "at risk") AND ("modelling*" OR "modeling*" OR "model*")

For reviewing the effects of control measures such as vaccination and behaviour change on the 2022-2023 mpox global outbreak, we searched PubMed and Embase for dynamic modelling studies on mpox outbreaks by May 18, 2025. Eligible studies employed transmission dynamic models (such as compartmental models, network models, or agent-based models) to simulate mpox epidemics during the 2022-2023 global outbreak, and to evaluate the effects of public health measures on the subsidence or control of the outbreaks. We focused on measures such as vaccination and behaviour adaptations, while not omitting studies that considered other reasons for their decline. We did not restrict the settings to country-level modelling, the language of articles, or whether studies performed calibration and validation against real-world case data. Studies that applied regression models, those that only reported modelling results of nowcasting and forecasting mpox outbreaks, or those not peer-reviewed were excluded. Cross-checking of references was performed to identify additional articles that might be relevant.

For the included modelling studies, the extracted information are as follows: title, authors, published year, the type of model constructed, model population (including sex where reported), the simulation time period and region, the method of model calibration and validation, mitigation measures implemented in the models with their initiation dates, the effects of measures in terms of the numbers or fractions of cases prevented or other outcomes, and the factors contributing to the containment of the 2022-2023 mpox outbreaks in each region.

For the quantitative synthesis, we included all empirical modelling studies (i.e., those that calibrated their models with the mitigation measures implemented in the real world to the observed epidemic data) that reported percentage of cases prevented by vaccination campaigns with or without the incorporation of behaviour change during 2022-2023. Modelling studies included in the review were excluded from the quantitative synthesis if they met any of the following criteria:
(1) theoretical simulation studies that did not calibrate their transmission dynamic models (with the mitigation measures implemented in the real world) to the observed epidemic curves; or
(2) empirical modelling studies that did not provide uncertainty intervals for the prevention percentages, or

sufficient information (e.g., uncertainty intervals for the model-estimated cumulative cases/infections under the corresponding scenarios) to derive them.

The PRISMA flow diagram (supplemental figure B) presents both the overall inclusion of studies in the review and those included in the quantitative synthesis.

Text B. Method: Meta-analysis

B.1 Mpox reproduction number

Descriptions on all the extracted R, arranged by their clades and periods of estimation, are presented in supplemental text D. For R estimates that indicative of intrinsic transmissibility of mpox and included in the quantitative synthesis, the mean or median of R estimates with uncertainty intervals (95% confidence intervals or credible intervals) were directly extracted from studies, or derived from reported interquartile ranges, standard deviations, or standard errors. When no uncertainty intervals were available, we approximated the standard error of R as $\sqrt{R}/\sqrt{N}$, where N represents the cumulative case count of the data used to simulate mpox reproduction numbers, assuming a Poisson distribution of case occurrences.

B.2 Prevention percentages for vaccination (either with or without the incorporation of behaviour change)

Effects of control measures implemented during the 2022–2023 mpox outbreaks, as estimated in empirical transmission dynamic modelling studies calibrated to observed epidemic curves, were reported using a wide range of outcome measures. A commonly reported outcome was the numbers or proportion of cumulative cases/infections prevented due to certain control measures, compared to counterfactual scenarios without interventions. Other reported measures included reductions in epidemic peak size, epidemic duration, and time-dependent reproduction numbers. As epidemic peak size and duration depend on size of susceptible population, and the decrease in time-dependent reproduction number is typically the result of depletion of susceptible hosts, these measures are not suitable for cross-study comparison.

In this meta-analysis, we chose to define the effect measure as the proportion of cumulative cases/infections averted (hereafter referred to as prevention percentages), given its greater robustness and suitability for cross-study comparison. As our focus was on the effects of the two control measures—vaccination campaigns and behaviour adaptation—and given the limited number of studies evaluating contact tracing and isolation, we included prevention percentages for vaccination with or without behaviour adaptation in our meta-analysis. Prevention percentage for other intervention strategies, as well as other reported outcomes from the included studies, are summarised in supplemental table G and supplemental text E – G.

The mean or median estimates of the prevention percentages, along with their uncertainty intervals (95% confidence intervals, credible intervals, or intervals derived from interquartile ranges) were directly extracted from studies, or calculated based on their reported numbers/infections of cumulative cases across different scenario simulations.

B.3 Meta-regression

For the three variables employed in our meta-regression of prevention percentages, values were collected as follows.

(1) The extent of behaviour adaptation, quantified as the proportional reduction in sexual contact rates, transmission probability per sexual contact, or number of sexual partners, relative to pre-outbreak levels, was directly extracted from the parameterisation section or calibration results of each modelling study. For models that incorporated time-varying functions for behaviour-related adaptations, half of the maximum reduction (representative value) was extracted. For prevention percentages attributed to vaccination alone, this value was set to 0%.

(2) First-dose mpox vaccine coverage was defined as the cumulative number of first doses administered by the time for estimating prevention percentages, divided by the model population of each study. Coverage was directly extracted from studies if values were reported, or calculated based on daily dose administration data provided by the studies or the national health authority.[1-4]

We chose to employ first-dose mpox vaccine coverage as the regression variable rather than full-dose coverage, considering that many countries, in response to limited vaccine supplies, initially prioritised administering first doses and expanding first-dose coverage by delaying second-dose administration until supply became more sufficient,[5] with estimated vaccine effectiveness being 76% for one dose and 82% for two doses based on results of a meta-analysis.[6]

(3) The number of weeks from the first case confirmation to vaccination initiation in the country or region of modelling was calculated by subtracting the two time points, as provided in the studies or reported by national health authorities,[4,7-12] and dividing the interval by seven (days per week).

Text C. Method: Risk of bias assessment

Risk of bias was assessed using a seven-item tool with a total score of 1 (higher scores indicate better quality), previously employed in a systematic review of transmission dynamic modellings and epidemiological parameters of Marburg virus disease,[13] to ensure quality of the methodological approaches in studies. The first two questions assess the clarity and reproducibility, robustness and appropriateness of the methods applied in

each study to obtain the statistical results, respectively.[13] The third and fourth questions measure whether the assumptions in the methods were clear, reproducible, and justified.[13] The last three questions evaluate whether the data used in the methods of each study were clearly described, whether issues in data were acknowledged, and whether the issues were resolved.[13]

Supplemental Results

Text D. Results: Description of the mpox reproduction number

D.1 Overview of the mpox reproduction number estimates (supplemental figure C)

From the mpox R estimates we included in the meta-analysis (in the main text), we also compiled a narrative plot of these R, arranged by their time period of estimates, and classify them according to their estimated clade and population (supplemental figure C). A trend of increasing R over time was observed, with estimates generally below 1 for clade Ia during 1970-2017, ranging between 1 and 2 during the 2017-2019 clade II outbreak in Nigeria, and rising to mostly above 2 (1.46 to 5.16) during the 2022-2023 clade IIb outbreaks, which predominantly affected the MSM population. The R for clade I mpox (Ia and Ib) after 2023 also exceeded 1 in South Kivu (1.18) and Sankuru province (1.29) of the DRC. Detailed illustrations for all the extracted R estimates, including their countries and periods of estimation, are illustrated in the following sections.

D.2 R of clade Ia mpox in the DRC between 1970 and 2017 (supplemental figure D)

The smallest R_t of mpox, 0.08, was the estimate of five Central and West African countries (including cases from both countries affected by clade Ia and those affected by clade II, with the majority of these cases [38 out of 47] reported in the DRC) during 1970-1979.[14] In general, the R estimates for clade Ia didn't exceed 1 and were lower than those for clade II.[15-18] One exception came from Grant et al.,[19] who derived the R_0 estimate, 2.13, for the DRC during 1980-1984 based on a previous estimate of 0.32 for mpox net reproduction number from Blumberg et al.,[15] and assumed an 85% effective coverage of vaccinia against mpox in the early 1980s.

D.3 R of clade II mpox in Nigeria between 2017 and 2022 (supplemental figure E)

The R_t estimates for clade II in Nigeria (subclade of these estimates not explicitly mentioned but most likely belonging to clade IIb) from 2017 to 2021 ranged between 1 and 2, with an R_t peak of 1.81 during the major mpox epidemic in Nigeria in 2017.[20] The same study also reported a historical peak of R_t (1.92) in Nigeria in 2022, plausibly linked to the relaxation of COVID-19 measures.[20]

D.4 R of clade IIb mpox during the 2022-2023 global outbreak (supplemental figure F)

For studies that estimated R in more than one country during the 2022-2023 mpox outbreak, we extracted only the R estimates of the country with the highest R values from each individual study. The R estimates for clade IIb mpox during the 2022-2023 global outbreak were significantly higher than those estimated in the endemic areas of Central and West African countries before 2022. These estimates ranged between 0.95 and 7.99, with the highest R_0 (7.99) estimated in the USA between May and July, 2022.[21] The maximum R values from

countries in the Americas and Europe were comparably high, depending on their periods and methods of estimation.

D.5 R of clade Ia and Ib mpox in the DRC after 2023 (supplemental figure G)

Of the two studies estimating R for clade I mpox in the DRC during the 2023-2024 outbreak, Marziano et al.[22] reported an R_e of 1.18 for clade Ib mpox in South Kivu province of the DRC, where the novel mpox subclade Ib was first identified and no mpox cases had been reported in this region until 2023.[23,24] The same study, on the other hand, reported an R_e of clade Ia and Ib mpox for the entire DRC to be below the epidemic threshold, ranging from 0.60 to 0.95 in 2024. Mbelambela et al.[25] estimated an R_0 of 1.29 in Sankuru Province of the DRC, where clade I mpox has long been endemic and was also one of the provinces most affected by the 2023-2024 clade Ib outbreak, alongside South Kivu.[26]

Text E. Results: Narrative review of the effects for control measures in empirical modellings

E.1 Behaviour adaptations

Most of the studies identified voluntary reductions in sexual behaviour among the MSM population, prompted by health education campaigns or in response to the mpox epidemic trend, as playing a crucial role in curbing mpox outbreaks. However, only three applied the results of empirical behaviour survey[27] or the data of activity on social media[28,38] to support parameterisation of behaviour adaptations. The majority employed curve-fitting to estimate the reduction of MSM sexual risk (reduction in the numbers of sexual contacts, the transmission probability per sexual contact, or the infectious period not refraining from sex), either after certain time points or using time-varying functions.

E.1.1 The UK

Brand et al.[29] fitted their model to the mpox epidemic curve in the UK, with R_t within the route of GBMSM sexual transmission dropped from 5.16 to 3.02 by 23 July 2022, and to 1.98 immediately after the WHO's announcement of PHEIC, assuming a continuous decrease in transmission probability per sexual act after gradually raised awareness. They estimated that behaviour adaptations played a predominant role in preventing approximately two-thirds of mpox cases in the UK by March 2023. Using a similar approach, Zhang et al.[30] estimated that 81.7% of mpox infections in the UK were averted by November 16, 2022, due to reduced sexual contact rate; when combined with a 20.0% reduction in effective infectious period, the two approaches collectively averted 98.0% of infections, which were instrumental in explaining the downturn of mpox outbreak in the UK.

E.1.2 The US

Four studies estimated the effect of sexual behaviour reduction in the US. Lin et al.[27] and Liang et al.[31] modelled behaviour-change-related reductions in transmission rates in the US and the Los Angeles County (LAC), based on survey results and curve-fitting, respectively. Clay et al.[28] assessed the reduction in daily one-time sexual contact rate in Washington D.C. by analysing the activity of mpox-related posts on social medias. Both Clay et al.[28] and Lin et al.[27] estimated that behaviour change of the high-risk population averted fewer cases than that of vaccination in the long run, accounting for a 25% and 15.4% of cases prevented in Washington D.C. by June 2023[28] and in the entire US by December 2022,[27] respectively. Furthermore, implementing only behaviour change leads to a flattened epidemic curve but not the diminishment of mpox transmission, resulting in an extended duration of outbreak compared to the scenario with no interventions in the US.[27,28] However, both studies emphasised the effect of immediate sexual risk reduction to allow time for the vaccination rollout and the induction period for the vaccines to take effect, which, although time-consuming, provided more prolonged protection against mpox infection. Liang et al.[31] highlighted both the importance of timely behaviour adaptations and vaccination from their analysis in LAC, where they found a 95% increase in cumulative cases if the reduction in sexual partnerships was delayed by three weeks. An alternative, minority view was presented by Zhang et al.,[32] who presented that the epidemic transitioned to a low-risk phase from August 6, 2022 to May 6, 2023 under a calibrated assumption of 84-86% reduction in transmission rate (sexual and non-sexual) due to behaviour change across the entire US population, with R_0 dropping from 3.1186 (as of July 30) to 0.4492. Under such assumption, the vaccination campaign, which was assumed to be proportionately distributed between MSM and non-MSM populations according to population size, would lead to only a non-significant reduction in R_e compared to the no-vaccination scenario, and was concluded to have limited impact on the outbreak trajectory by this study.

E.1.3 The Netherlands

Contrary to the predominant findings in the US, Xiridou et al.[33] suggested that the decline of mpox in the Netherlands was primarily due to infection-induced immunity in the highly sexually active MSM, accelerated by behaviour adaptations. From their simulations, Xiridou et al.[33] revealed a less steep decline in the epidemic trajectory and a delayed fade-out of mpox in the absence of behaviour adaptations, which supported the effect of behaviour change on facilitating the subsidence of mpox.

E.1.4 Italy

Guzzetta et al.[34] showed that behaviour change in MSM communities was the main driver of the rapid decline of mpox epidemic in Italy as well. They calibrated that a 61% and 87% decrease in the transmission rate in sexual network and in clubs, respectively, sharply cut the overall reproduction number from 1.97 to 0.87, and also interrupted local transmission chains.

E.1.5 Canada

For the mpox outbreak in Canada, Milwid et al.[35] attributed its downturn to behaviour change. They calculated that the 51% reduction in transmission rate by July 27, 2022 lowered the epidemic peak in Canada from about 4200 cases per day to 30 cases per day, and also shortened the outbreak duration. Conversely, Xiu et al.[36] calibrated a much more conservative 6% reduction in the number of sexual partners, which was estimated to avert 10-15% of mpox infections in the three Canadian cities of Montréal, Toronto, and Vancouver.

E.2 Vaccination

E.2.1 The US

The vaccination campaign in the US successfully averted a substantial proportion of mpox cases that would have otherwise occurred. Clay et al. and Lin et al. estimated that the targeted vaccination programme alone averted 79% and 21.2% of cases in Washington D.C.[28] and in the entire US,[27] respectively. Liang et al.[31] and Xiong et al.,[37] in contrast, estimated that the cumulative cases in LAC and New York City (NYC) would surge by 28% and 385%, respectively, if the current vaccination campaign were not implemented. Clay et al.,[38] who also modelled the mpox outbreak in NYC, found that by adopting first-dose prioritisation vaccination strategy (status quo) in NYC, 67% of cases were averted compared to the counterfactual without vaccination, and averted an additional 0.61% or 17% more cases compared to the counterfactual scenario in which the intermediate strategy (prioritising vaccine supplies to second-dose-eligible individuals, without preallocating doses) or the second-dose priority strategy (preserving each person's second dose upon first-dose administration) were implemented, respectively. A key phenomenon observed by Lin et al.[27] is the synergistic effect between high-risk group awareness and vaccination campaign, which together prevented a greater number of cases (64.0%) than the sum of percentages prevented when each of the two measures was implemented alone. This was also reflected by the results of Zhang et al.[32] in the US, stating that the assumed proportionate vaccination of MSM and non-MSM populations according to population size did not lead to significant decline in mpox epidemic.

E.2.2 The UK

The vaccination programme in the UK, however, provided relatively minimal effects, averting 9.8% of infections when implemented alone.[30] It leads to only a marginal increase of 0.1% in cases averted when being an adjunct to behaviour change and the reduced effective infectious period.[30] Brand et al.[29] concurred that the epidemic curve in a counterfactual scenario without vaccine administration did not deviate much from what was actually observed by September 2022 in the UK. Nevertheless, both studies underscored the role of vaccination-induced immunity in preventing future resurgences of mpox, which will be discussed in further sections.[29,30]

E.2.3 The Netherlands and Italy

In the Netherlands and Italy, the decline of epidemic trajectories began before the initiation of national vaccination programmes. Shamier et al.[39] simulated that the vaccination campaign in the Netherlands yielded only a 7% decrease in cumulative cases during the 2022-2023 outbreak.

E.2.4 Canada

In Canada, conclusions diverged between Milwid et al. and Xiu et al.[35,36] While Milwid et al.[35] showed that under the status quo scenario with vaccination and behaviour adaptation, varying the vaccine effectiveness and the proportion of GBMSM vaccinated demonstrated minimal impact on the epidemic projections, Xiu et al.[36] estimated that the early vaccination in Montréal, Toronto, and Vancouver contributed the greatest share of cases prevented (21–39%) among the three interventions, and, together with behaviour adaptation and contact tracing, averted 46–58% of cases that would have otherwise occurred.

Text F. Results: Theoretical simulations of vaccine allocation and public health strategies in the 2022-2023 mpox clade IIb global outbreak and clade I outbreaks after 2023

F.1 Modelling studies targeting the 2022-2023 global outbreak

Twenty-four theoretical simulation studies explored different vaccination and control strategies under hypothetical or country-based scenarios. Dimitrov et al.[40] tested the potential benefit of the fractional vaccine administration strategy to spare a full dose of mpox vaccine into 3.5 fractional doses within the MSM population of Seattle, USA. Knight et al.[41] simulated the mpox outbreak between two partially connected GBMSM sexual networks (cities) in Ontario, Canada. The optimal strategies of vaccine allocation between two cities were tested under conditions considering the relative size and epidemic potential of each city, as well as the degree of inter-city mixing. Gan et al.[42] simulated mpox outbreaks in Singapore, Hong Kong, and Sydney, characterised by varying patterns and densities of sexual contact networks, and evaluated the effect of pre-emptive or immediate vaccination upon notification of the first case on cessation of the epidemic. Savinkina et al.[43] and Huang et al.[44] simulated mpox outbreaks in hypothetical college cohorts, considering a combination of control measures to develop effective strategies and decision-support tools for the impediment of mpox transmission in campus settings. Bragazzi et al.,[45] Cao et al.,[46] Chaturvedi et al.,[47] Chitwood et al.,[48] Cordeiro et al.,[49] Haridas K. Das,[50] Idisi et al.,[51] Khan et al.,[52] Ko et al.,[53] Leandry et al.,[54] Musafir et al.,[55] Van Dijck et al.,[56] Spicknall et al.,[57] Yang et al.,[58] Yuan et al.,[59] and Yuan et al.,[60] assessed how the implementation of mitigation measures, such as detection and isolation of cases, contact tracing and quarantine, sexual behaviour adaptations, restrictions on attendance at mass gatherings events, and PrEP or PEP vaccination, across a range of exploratory scales, could contribute to the prevention of mpox cases and the suppression of sustained mpox transmission in various settings: Canada, the US, Berlin city, the US, Portugal, multiple countries and continents, Nigeria, the

UK, a simulated country non-endemic to mpox, a simulated setting with human and rodent sub-populations, the US, Belgium, a Washington D.C.-like area, multiple countries, a hypothetical city considering sexual contacts at gathering events, and a hypothetical metropolitan area with animal-to-human transmission, respectively. Jia et al.,[61] who considered the transmission of animal-to-human in Canada, evaluated the final epidemic size under the various level of reduction in contact rate of the high-risk humans and between humans and infectious animals, placing the concern of epidemic resurgence in the low risk human as the transmission risks in high-risk group had been controlled. Omame et al.[62,63] constructed co-infection models of mpox and HIV to test how adaptations in sexual behaviours, along with the coverage and efficacy of antiretroviral treatment, influence mpox dynamics when HIV infection increases susceptibility to mpox, and subsequently extended their model to perform stability and bifurcation analyses under HIV-endemic conditions.[63]

F.2 Modelling studies targeting the DRC clade I outbreak since 2023 and importation-triggered clade Ib outbreaks in Asia

A modelling study by Savinkina et al.[64] examined the effect of pre-emptive vaccination strategies and resource needed in the DRC clade I outbreak since 2023. Considering clade Ia sylvatic transmission as the predominant cause of mpox in the DRC during 2023, Savinkina et al.[64] estimated that vaccinating children under the age of 5 or 15 in endemic areas of the DRC would be more efficient, potentially averting 27% or 54% of overall mpox cases, and 43% or 71% of overall mpox deaths, respectively, when reaching a vaccination coverage of 80%. Jin et al.,[65] on the other hand, simulated mpox clade Ib outbreaks in 37 Asian cities following one-time importation events, considering the varying immunity levels from smallpox vaccination, and proportions of high-risk individuals in each population. Their exploration of the effectiveness of two non-pharmaceutical interventions revealed that, under the scenario with three initial local infections, isolation alone could prevent 43.8% to 88.0% of cumulative cases within one year, and could reduce the cumulative case count by an additional 22.0% to 8.4% when combined with quarantining close contacts, across scenarios with 25% to 100% reductions in interpersonal contacts.

Text G. Results: Possibilities of mpox resurgences under various conditions

Despite the waning of the mpox epidemic in most European and American countries by the end of 2022, studies assessed the risk of mpox resurgence under various circumstances. Importation of new cases was thought to be less likely to cause resurgences of mpox epidemic. Xiridou et al.[33] simulated an importation of 20 cases to the Netherlands on 1 November, 2022 or 1 case per day from November to December with behaviour adaptations in July 2022, causing only a small increase of 41 or 81 cumulative cases by the end of 2022. In addition, consistent case importation of more than 5 cases per 10,000 GBMSM in Canada was necessary to induce a sustained mpox resurgence in the model of Milwid et al.[35] Rebounds toward risky sexual behaviours may cause mpox resurgences of mpox when they occurred early and on a large scale. Given all other conditions identical to the status quo, Liang et al.[31] simulated that a half rebound of sexual behaviours in December 2022 would only cause

a 0.09% increase in cumulative cases by April 2023 in the Los Angeles County; however, cases surged by 453% if a full rebound occurred as early as October 2022. The replenishment of susceptibility due to demographic turnover could also cause future mpox outbreaks, as indicated by Shamier et al.,[39] simulating outbreak sizes ranging from 254 to 725 over the next 10 years if vaccination of the high-risk population was discontinued in the Netherlands. With regard to the possibility of mpox resurgences, it is crucial that targeted vaccinations play a role in preventing future outbreaks, as evidenced in the main text.

Text H. Results: Risk of bias assessment

The results of risk of bias assessments are summarised in supplemental figures K–M. The average score for the overall 81 papers is 0.630, with a trend of higher scores for papers published after 2022. Papers on reproduction number estimates (R) after 2022 had, on average, the highest assessment scores (0.674) but also the greatest variability, followed by empirical modelling papers (0.670, all of which were published after 2022), papers on reproduction number estimates before 2022 (0.587), and theoretical simulation modellings (0.546). For the distribution of scores on each question, the number of 'No' and 'NA' was highest on the last question, which assessed whether issues in the data were accounted for in the chosen methodology.

Supplemental Figure A. PRISMA flow diagram for the review on estimates of mpox reproduction number.

Reasons for studies excluded for the review by full-text reading, and included in the review but ineligible for quantitative synthesis, are presented in supplemental tables B and D, respectively.

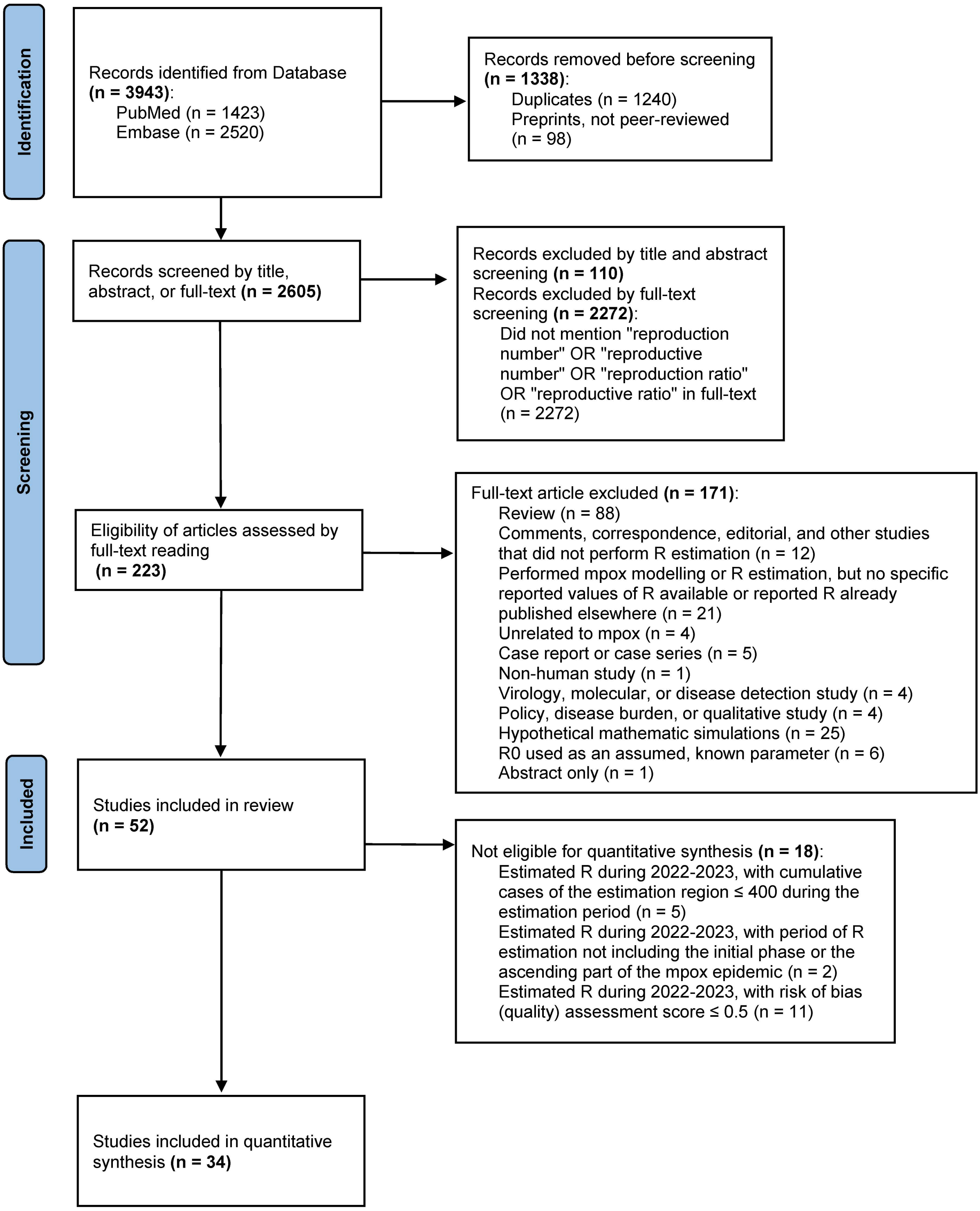

Supplemental Figure B. PRISMA flow diagram for the review on the effects of mpox mitigation measures assessed by transmission dynamic model.

Reasons for studies excluded for the review by full-text reading, and included in the review but ineligible for quantitative synthesis, are presented in supplemental tables C and E, respectively.

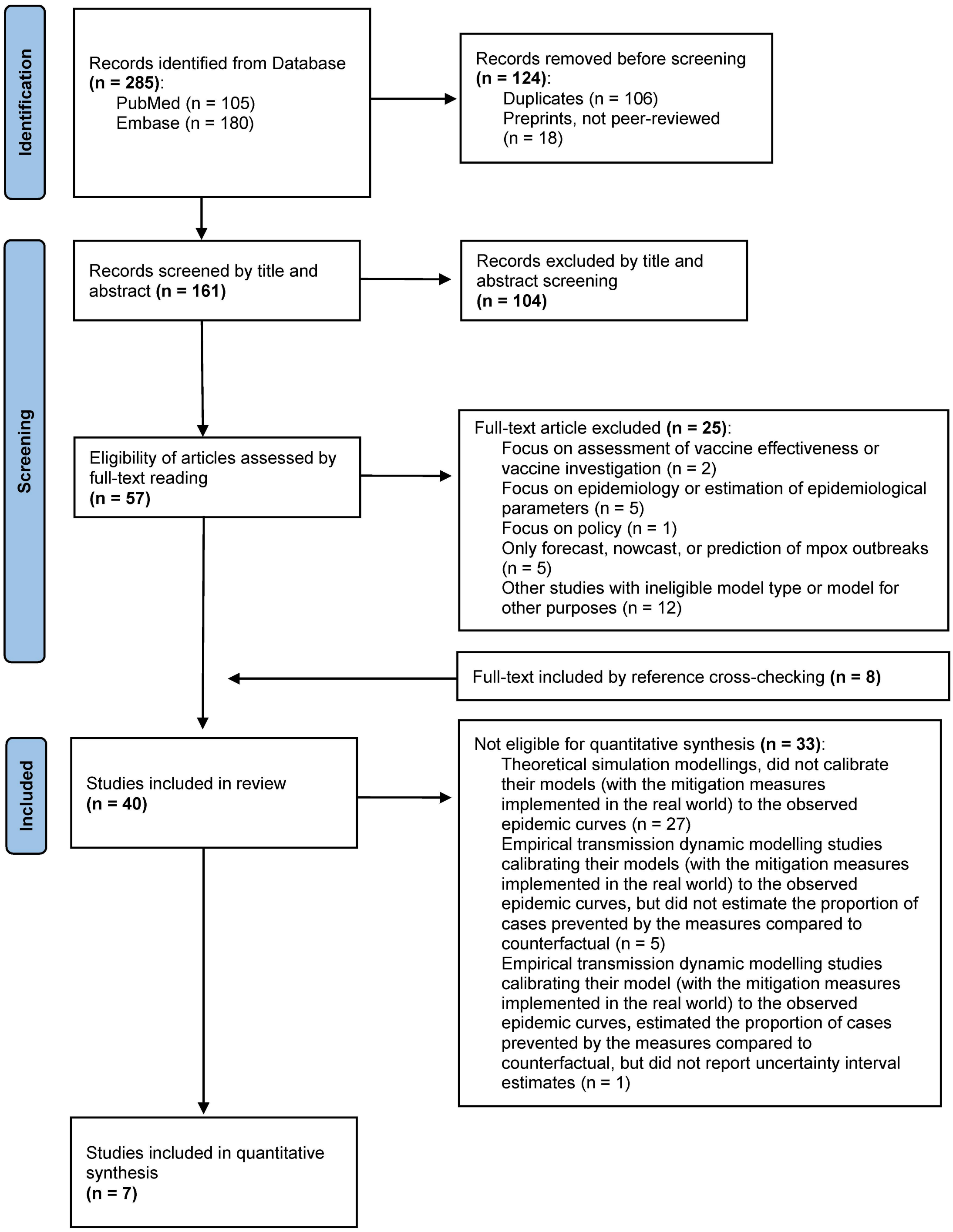

Supplemental Figure C. Time series plot of mpox R estimates (R_0, R_t or R_e) and their 95% confidence or credible intervals (interquartile range shown when 95% CI or CrI weren't available) from studies included in the meta-analysis. R estimates are presented in the same order as in figure 2 of the main text.

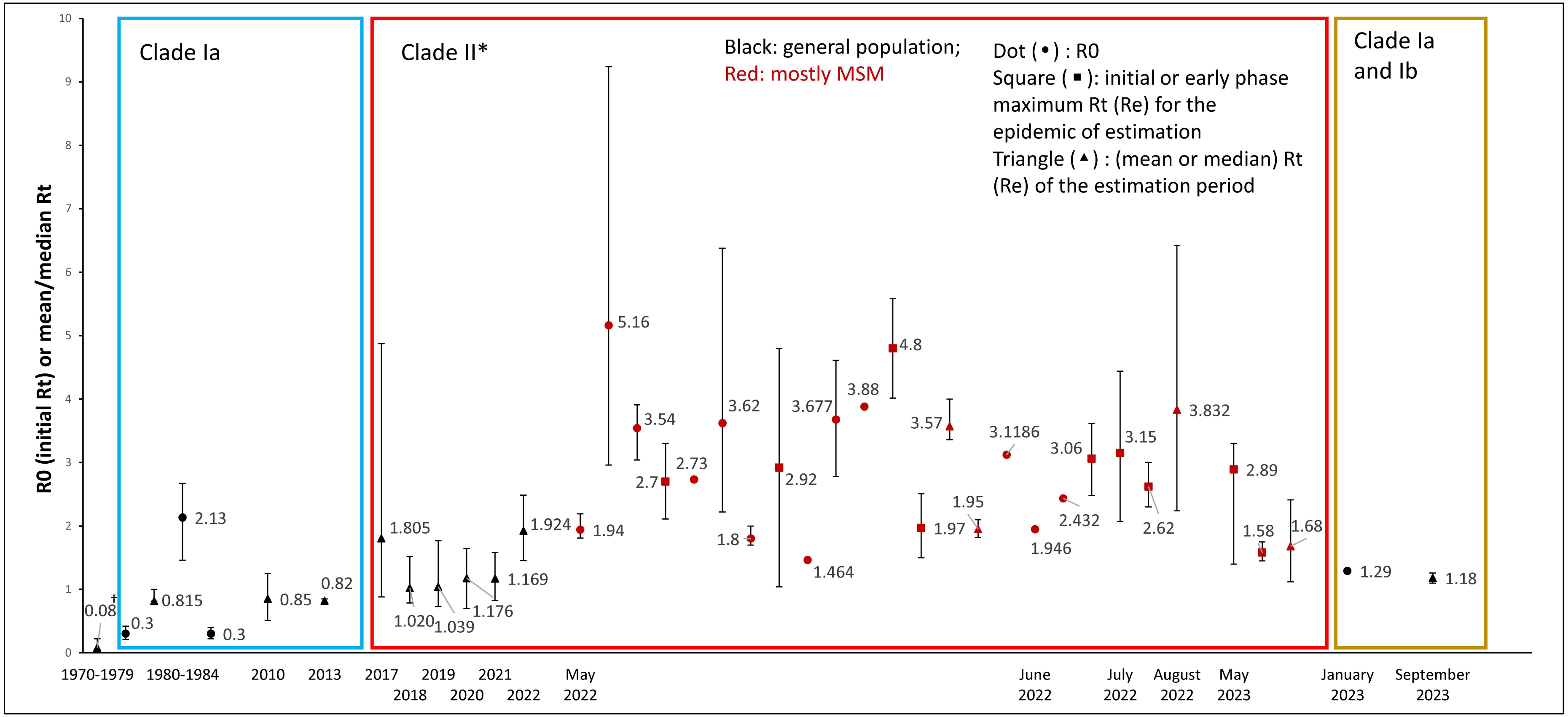

* R estimates of clade II before 2022 (2017-2021) predominately derived from Nigeria, where outbreak strains were mostly assigned to clade IIb lineage A.[67,68] R estimates after 2022 were predominantly based on the global outbreak of clade IIb.

† R_e estimated from countries affected by either clade Ia or clade II (IIa) outbreaks.

Supplemental Figure D. Time series plot of the reproduction number estimates of mpox clade I before 2022, from all the included studies.

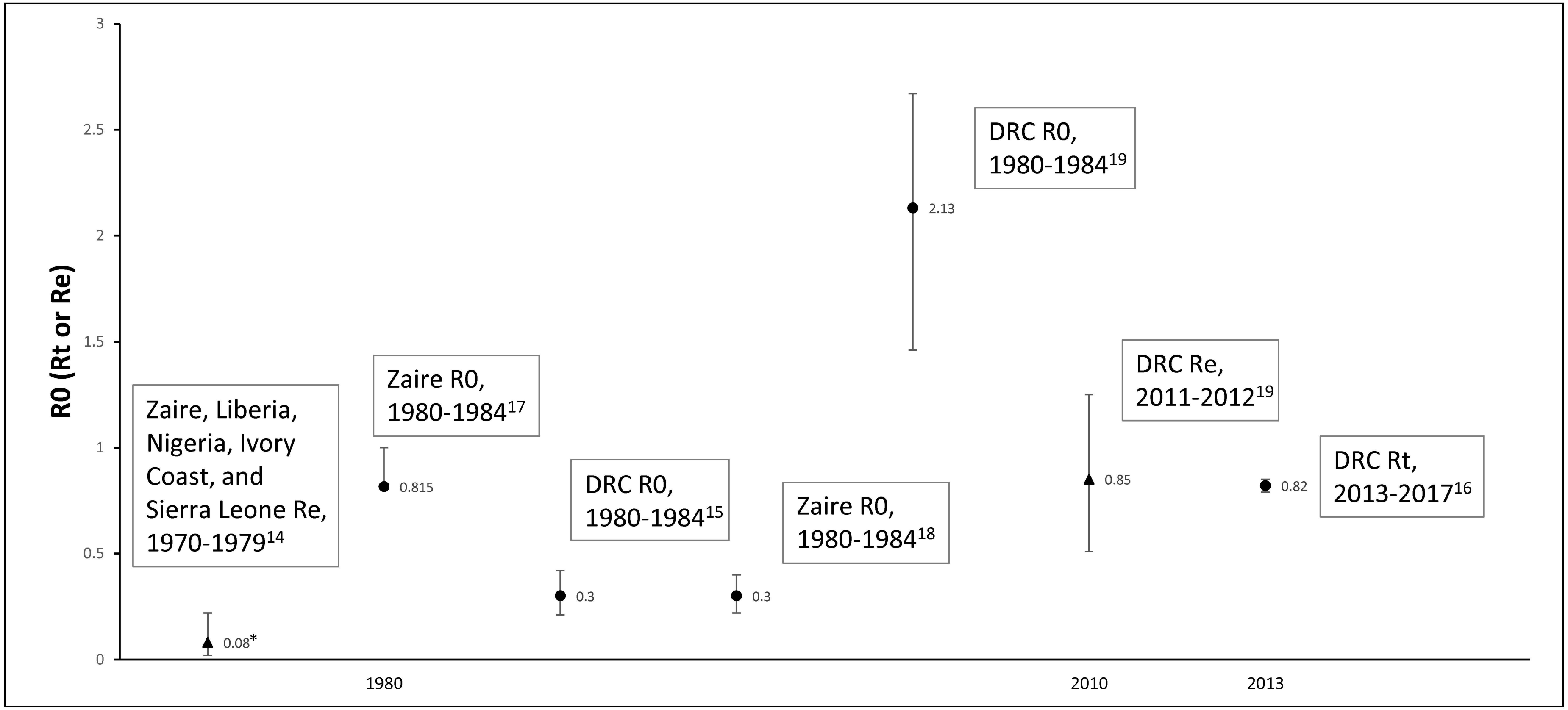

R_0 are represented with dots, R_t or R_e are represented with triangles. R estimated for the general population are shown in black, while those estimated during outbreaks where the MSM population was mostly affected were shown in red.

* R_e estimated from countries affected by either clade Ia or clade II (IIa) outbreaks.

Supplemental Figure E. Time series plot of the reproduction number estimates of mpox clade II before 2022, from all the included studies.

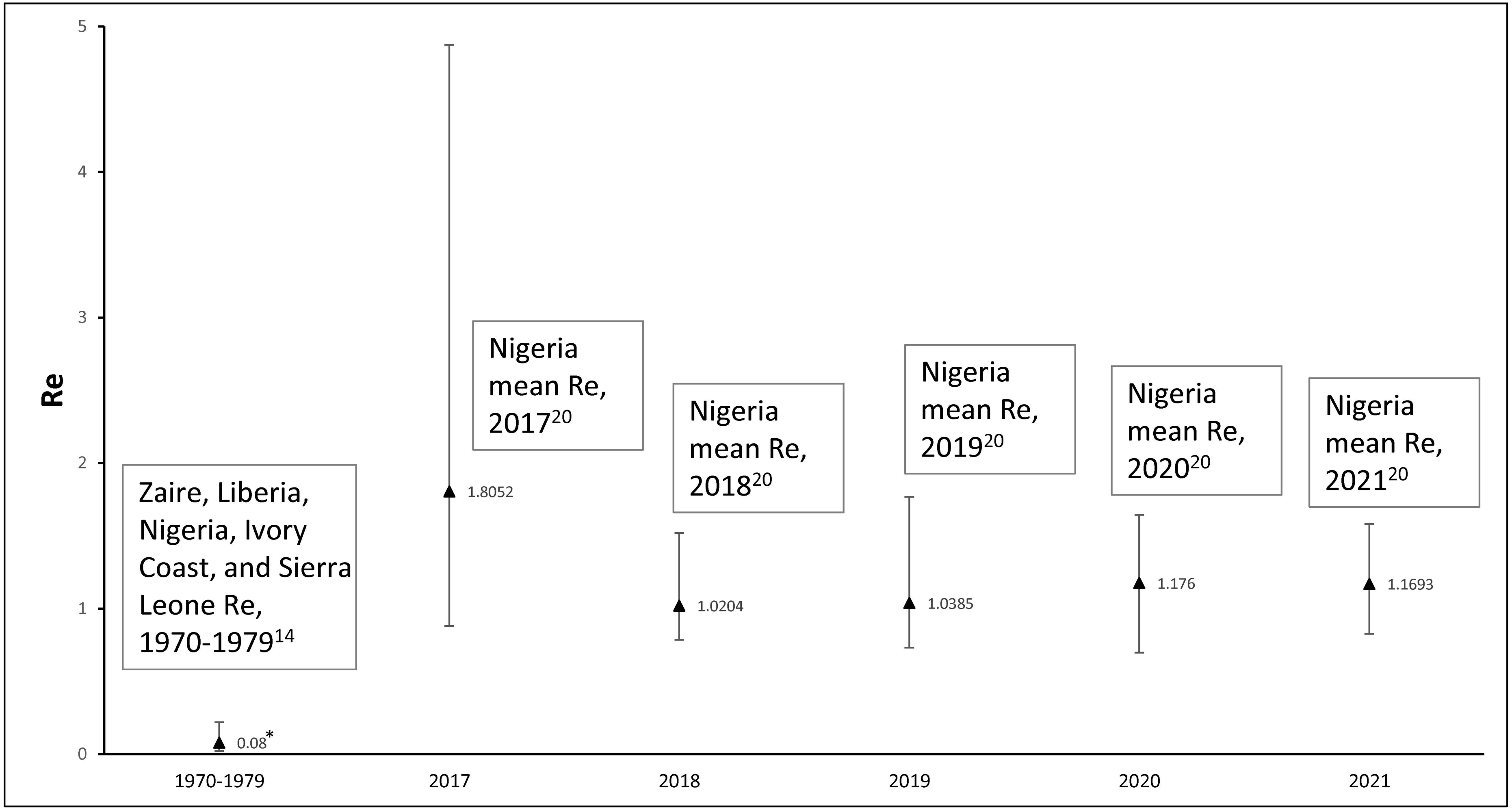

R_0 are represented with dots, R_t or R_e are represented with triangles. R estimated for the general population are shown in black, while those estimated during outbreaks where

the MSM population was mostly affected were shown in red. R estimates of clade II between 2017-2021 predominantly derived from Nigeria, where outbreak strains were mostly assigned to clade IIb lineage A.[67,68]

* R_e estimated from countries affected by either clade Ia or clade II (IIa) outbreaks.

Supplemental Figure F. Time series plot of the reproduction number estimates of mpox clade IIb after 2022, from all the included studies.

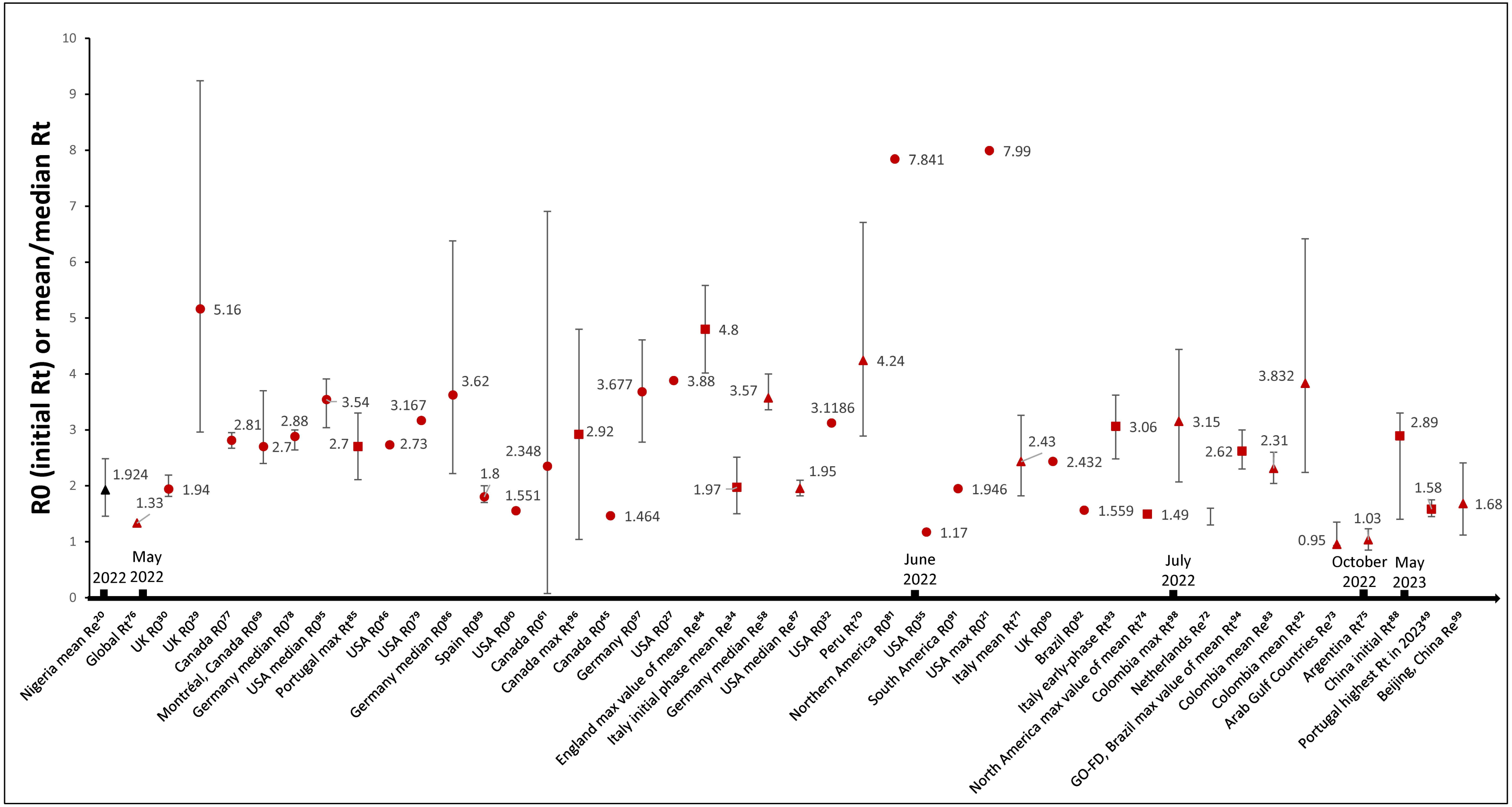

R_0 are represented with dots, initial or early phase R_t (R_e) for the epidemic of estimation are represented with squares, and mean or median R_t (R_e) of the estimation period are represented with triangles. R estimated for the general population are shown in black, while those estimated during outbreaks where the MSM population was mostly affected were shown in red. For studies that estimated R for more than one country during the 2022-2023 mpox outbreak, we present only the R estimate of the country with the highest R values from each individual study.

Supplemental Figure G. Time series plot of the reproduction number estimates of mpox clade I (Ia + Ib) after 2023, from all the included studies.

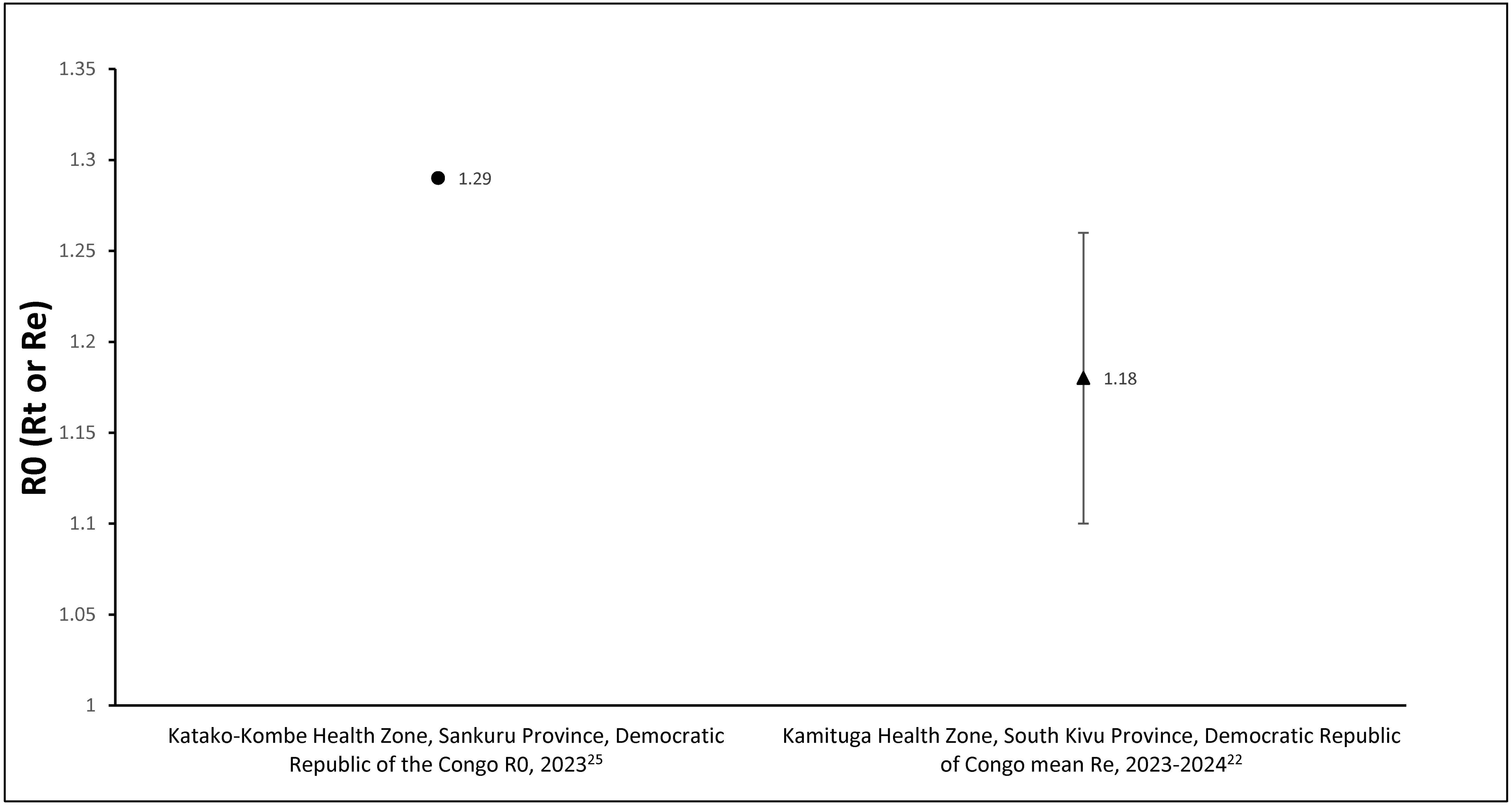

R_0 are represented with dots, R_t or R_e are represented with triangles. R estimated for the general population are shown in black, while those estimated during outbreaks where the MSM population was mostly affected were shown in red.

Supplemental Figure H. Forest plots of mpox reproduction number estimates for (A) clade I and (B) clade II, grouped by period of estimation and type of reproduction number estimated.

(A)

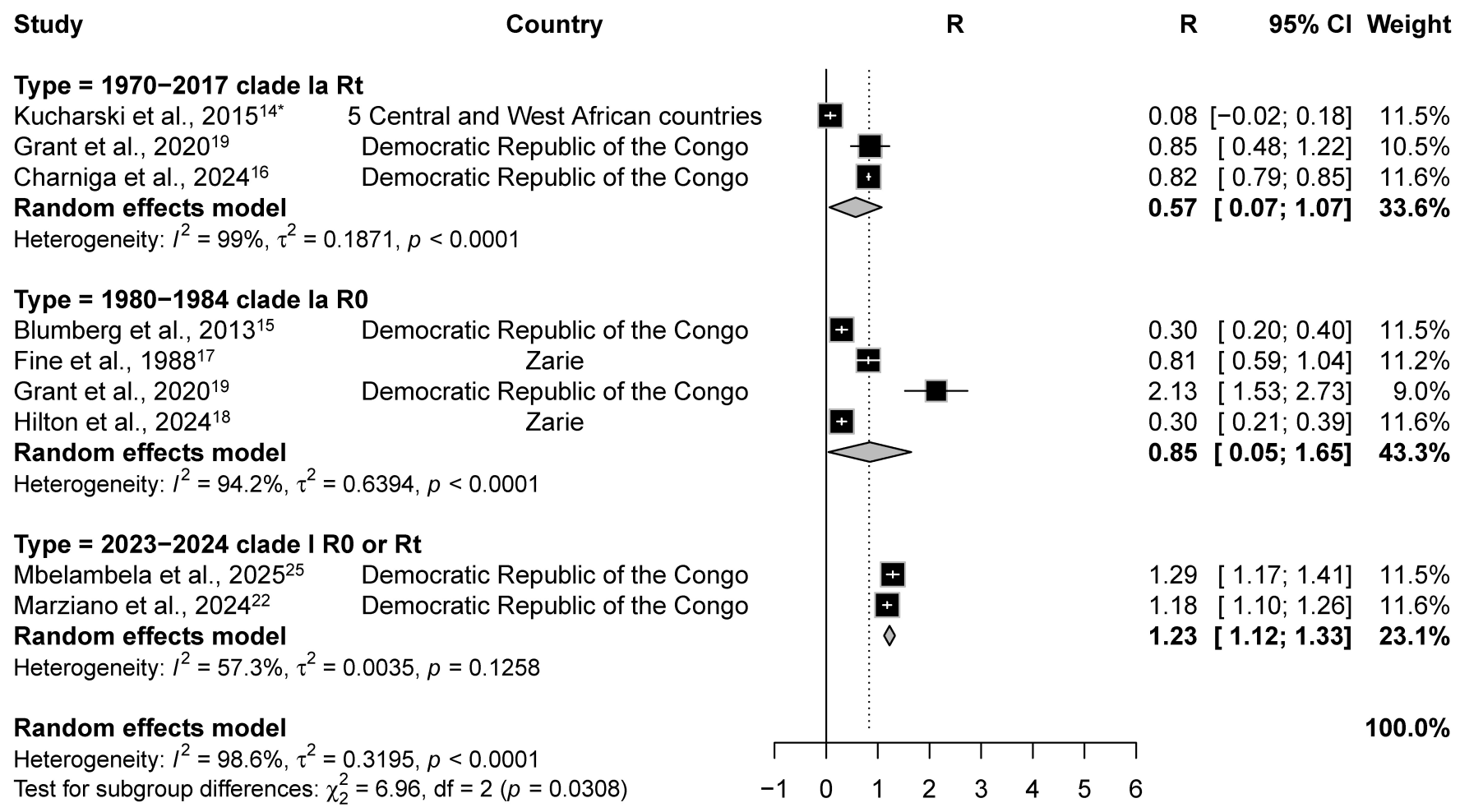

(B)

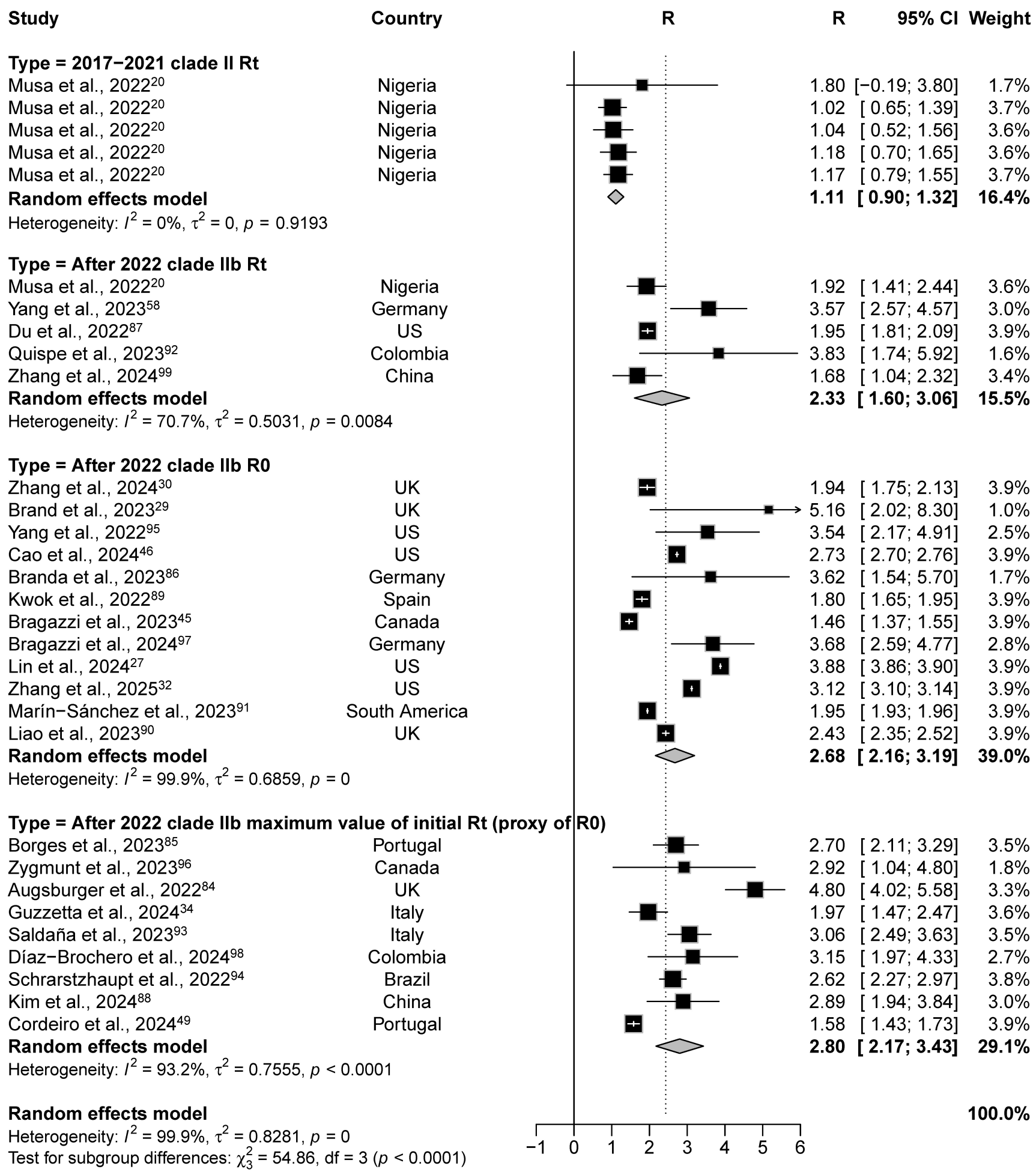

* The R estimated by Kucharski et al., 2015[14] was derived from cases in five Central and West African countries affected by either clade Ia or clade II (IIa) outbreaks. As the majority of these cases (38 out of 47) was reported in Zaire (the former name of the Democratic Republic of the Congo [DRC] from 1971 to 1997), which was affected by clade Ia outbreaks during the period of R estimation, we categorised this estimate under clade Ia.

Supplemental Figure I. Funnel plots of R estimates included in the meta-analysis of mpox reproduction numbers for (A) clade I and (B) clade II, with pseudo 95% confidence limits.

(A)

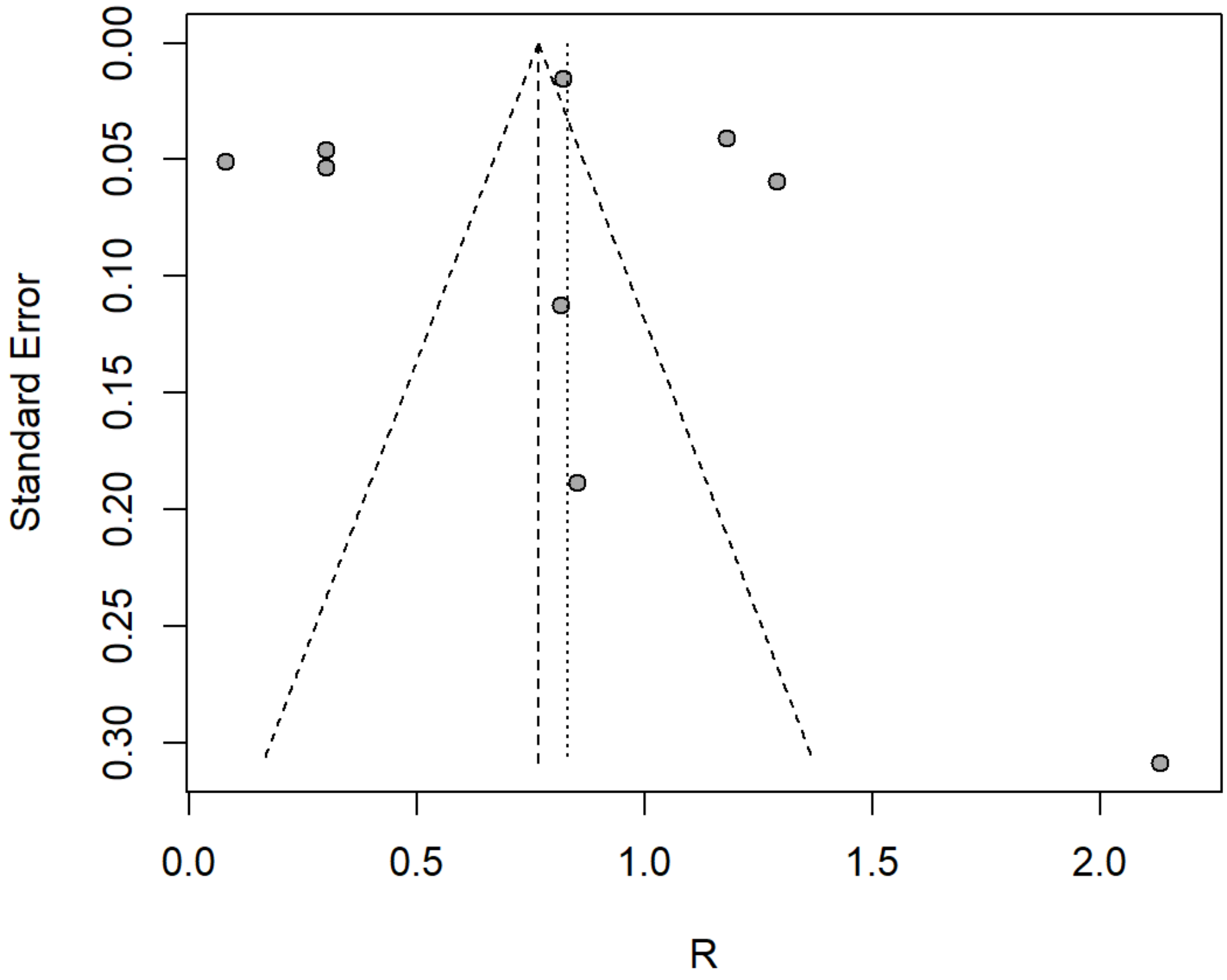

Egger's test indicated no significant publication bias (p=0.803).

(B)

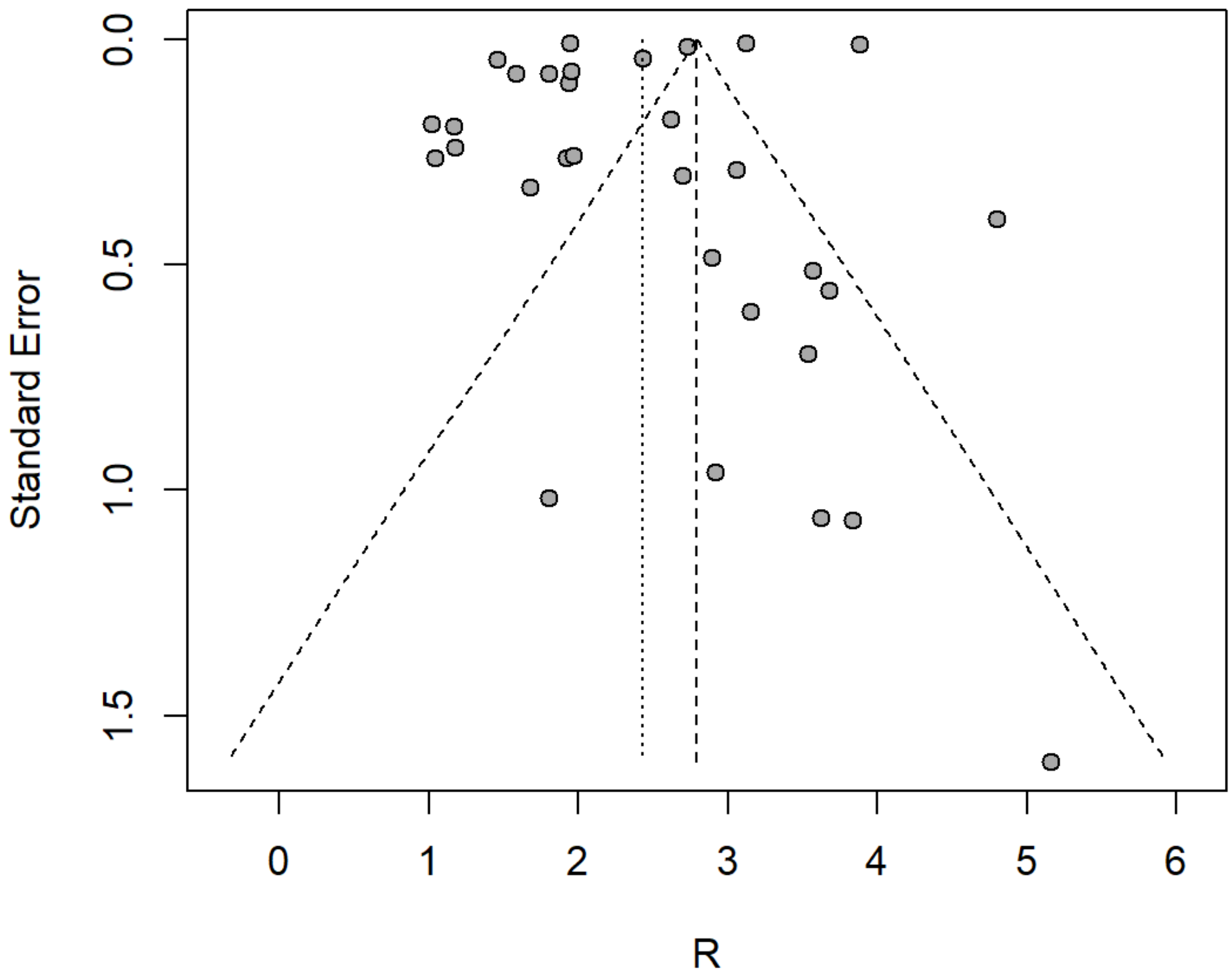

Egger's test indicated no significant publication bias (p=0.522).

Supplemental Figure J. Funnel plot of all prevention percentages included in the meta-analysis, with pseudo 95% confidence limits.

Prevention percentages are shown in decimal form (e.g., 0.2 = 20%).

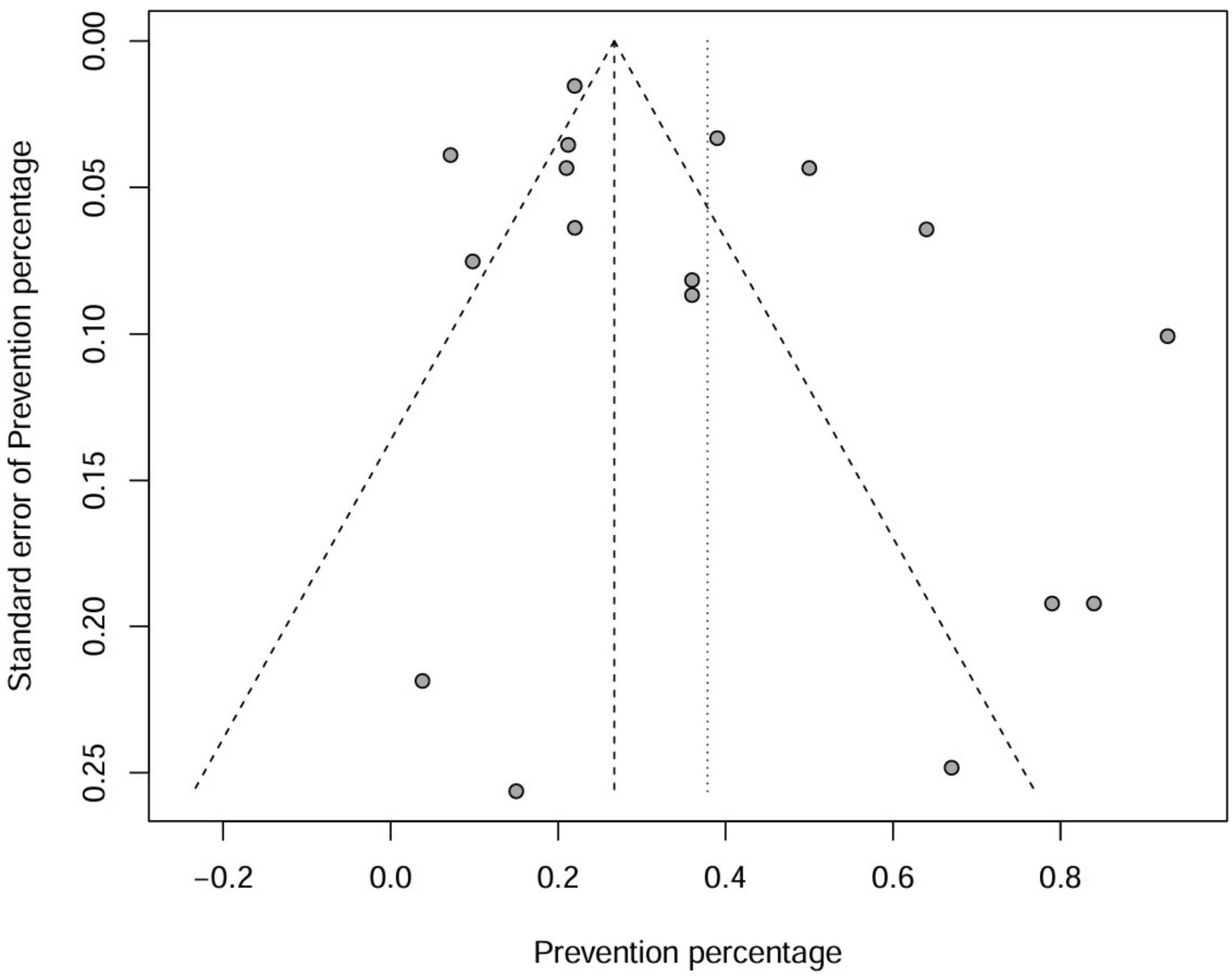

Egger's test indicated no significant publication bias (p=0.0874)

Supplemental Figure K. Trends in risk of bias assessment scores by publication year.

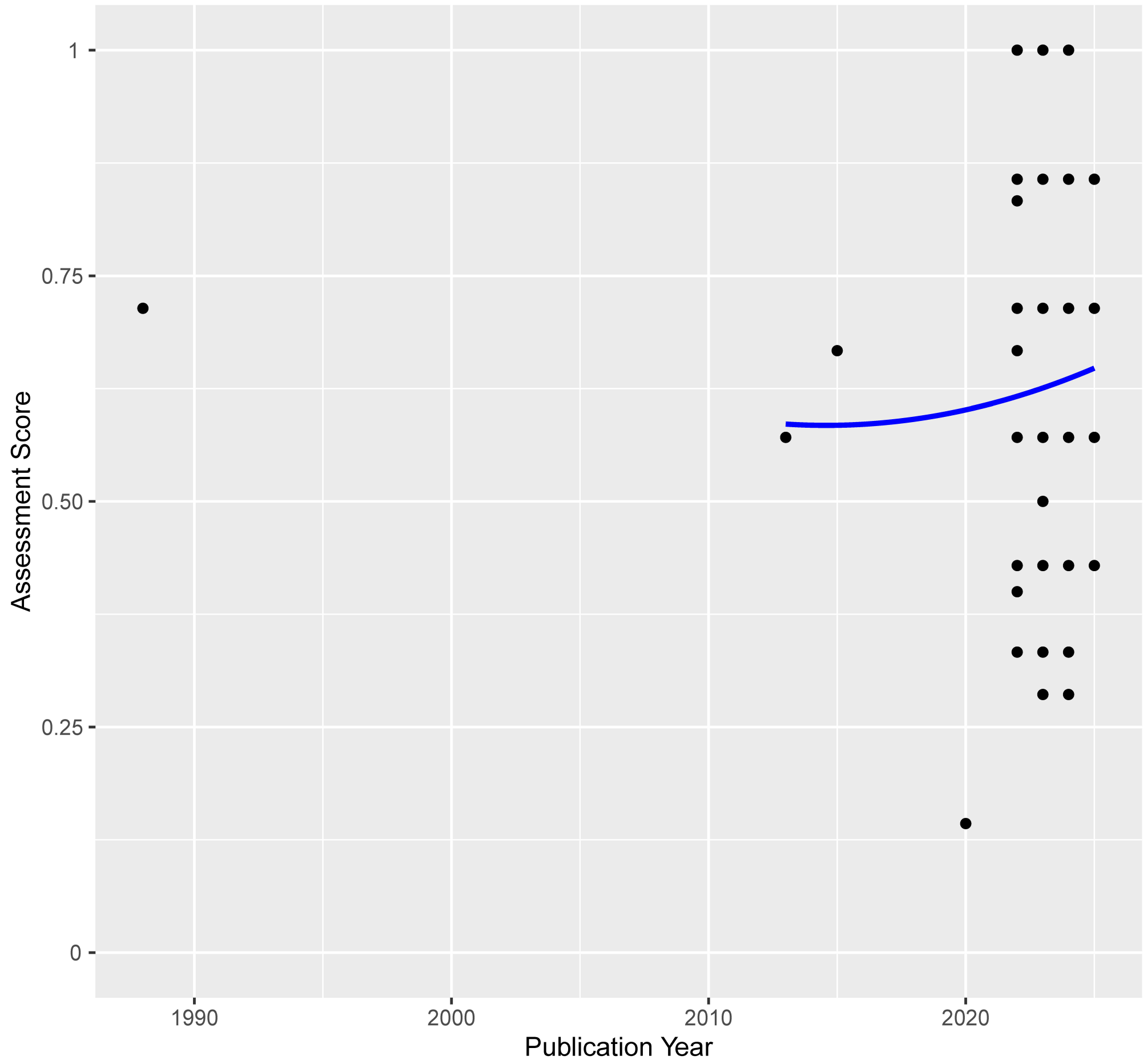

Supplemental Figure L. Boxplot of risk of bias assessment scores by study type.

Empirical modellings refer to studies that calibrated their mpox transmission models (with mitigation measures implemented in the real-world status quo scenario) to their respective epidemic curves. Theoretical simulation modellings refer to studies that did not calibrate their model to real-world epidemics or models that performed calibration but assessed the potential effects of public health interventions across exploratory scales.

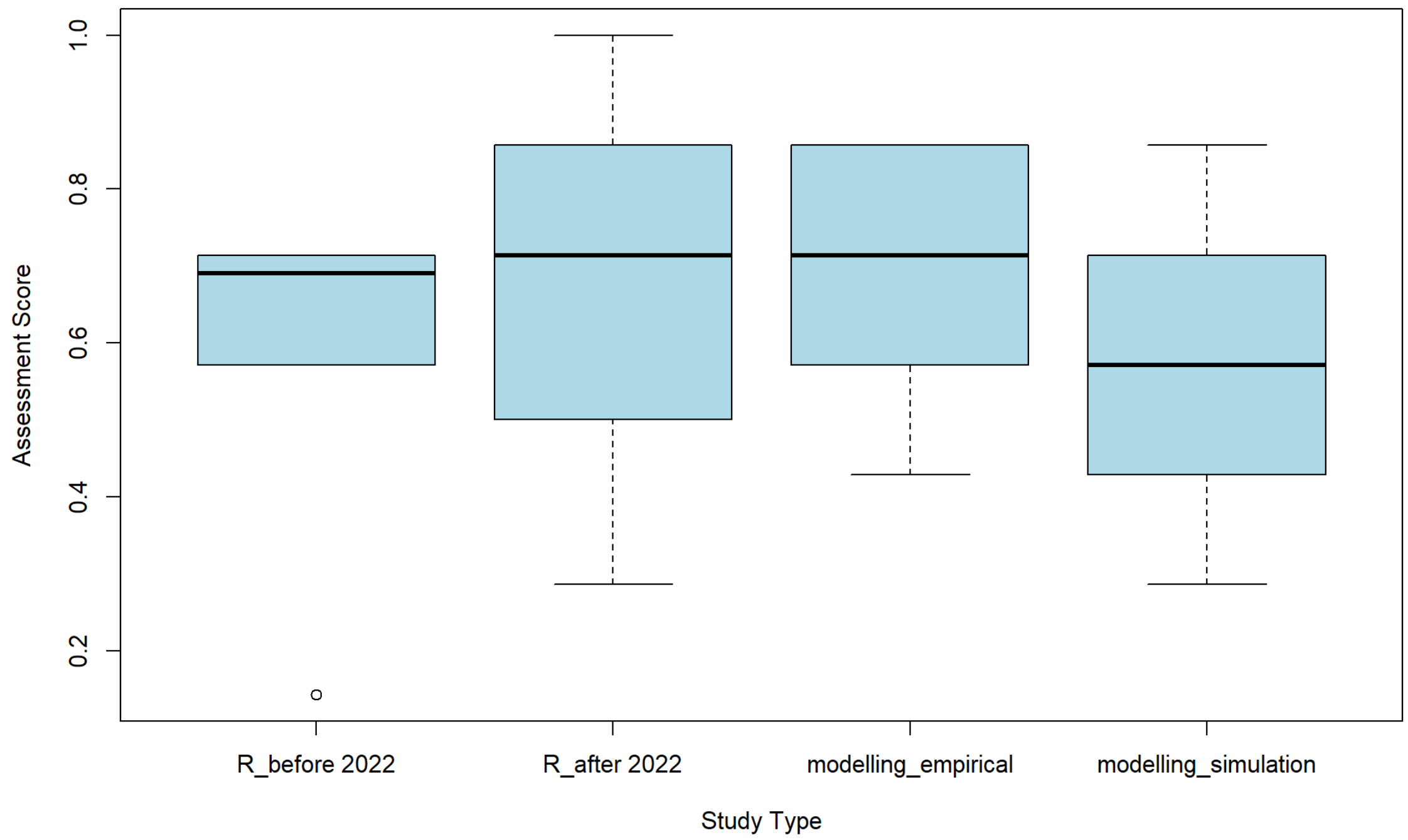

Supplemental Figure M. Proportion of studies rated Yes, No, or NA for each of the seven risk-of-bias assessment items.

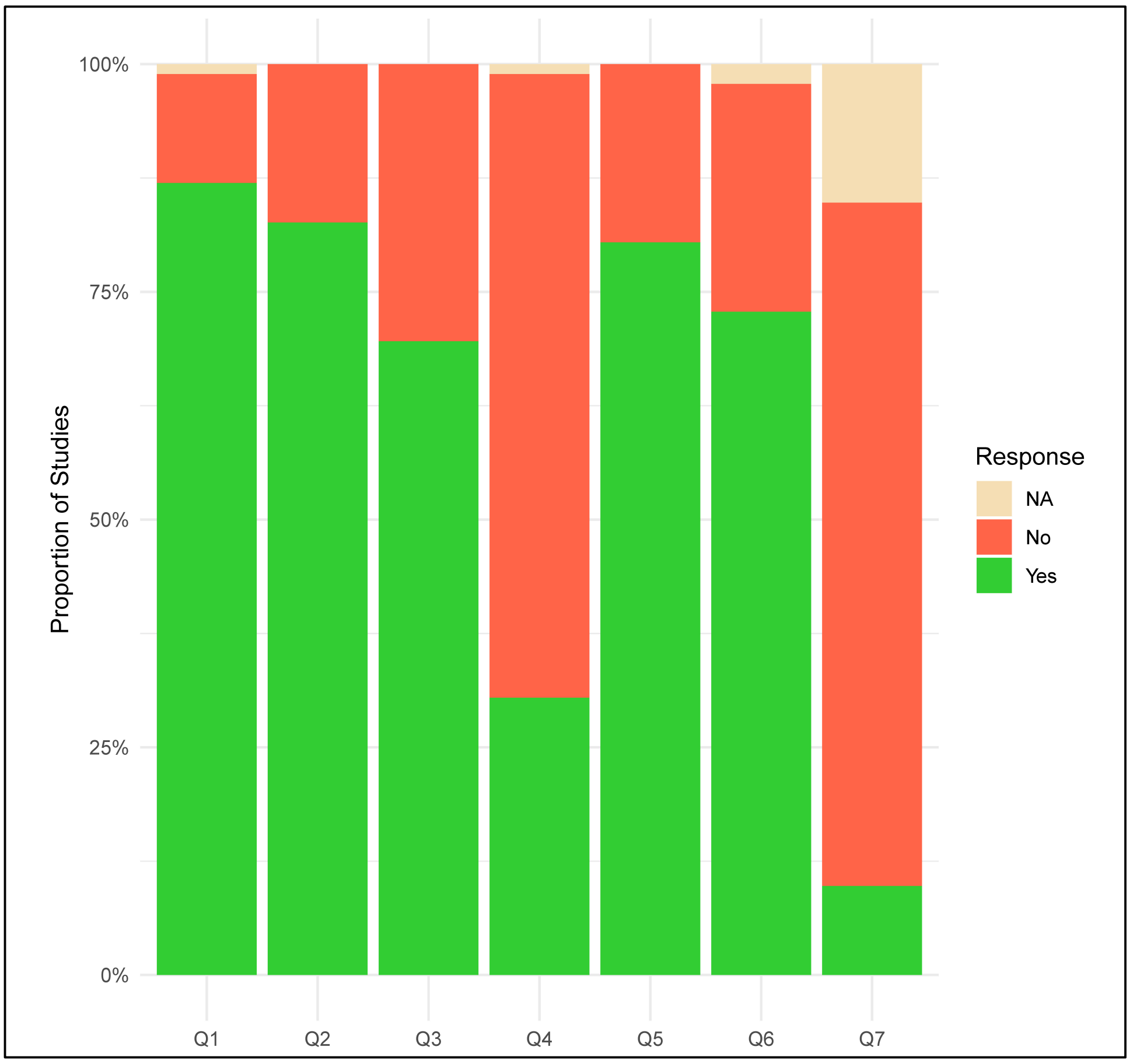

Supplemental Figure N. Leave-one-out forest plot of mpox R estimates.

(i) Mpox R estimates, within the subgroup of clade I before 2022
The pooled R estimate in the subgroup of clade I before 2022 ranged from 0.51 to 0.82, when each study was omitted in turn. The lowest pooled R (0.51) was observed when omitting Grant et al., 2020, who estimated an R_0 of 2.13 for the DRC during 1980–1984, suggesting that this relatively high estimate may have exerted upward influence on the pooled effect.

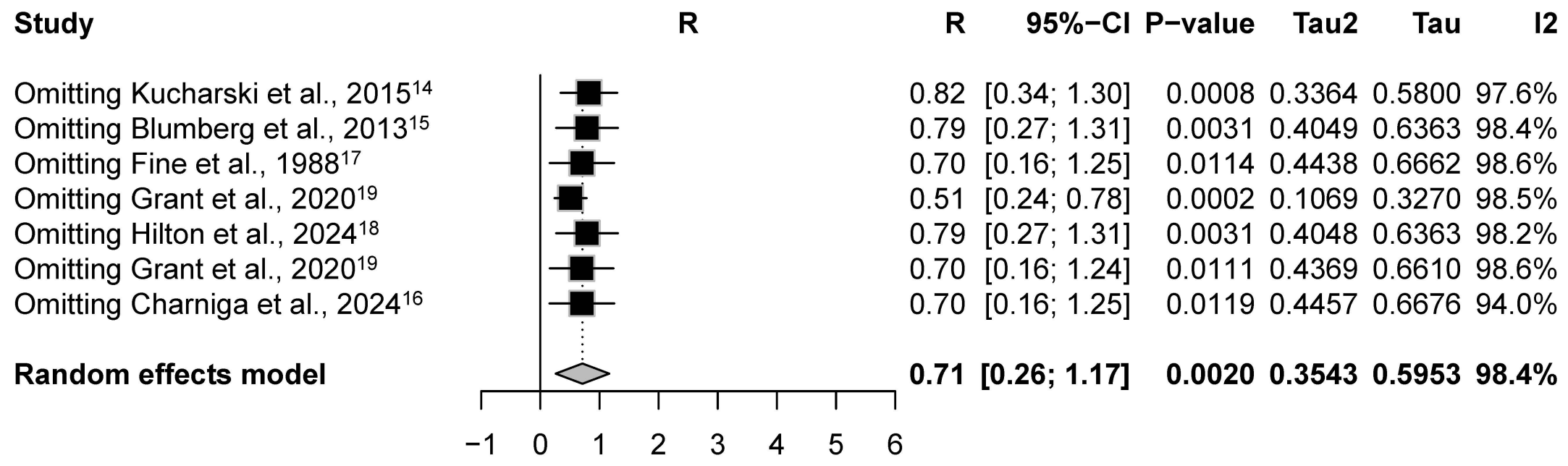

(ii) Mpox R estimates, within the subgroup of clade I (Ia and Ib) after 2023
The pooled R estimate in the subgroup of clade I (Ia and Ib) after 2023 ranged from 1.18 to 1.29, when each study was omitted in turn (shown for completeness; only two studies were in this subgroup).

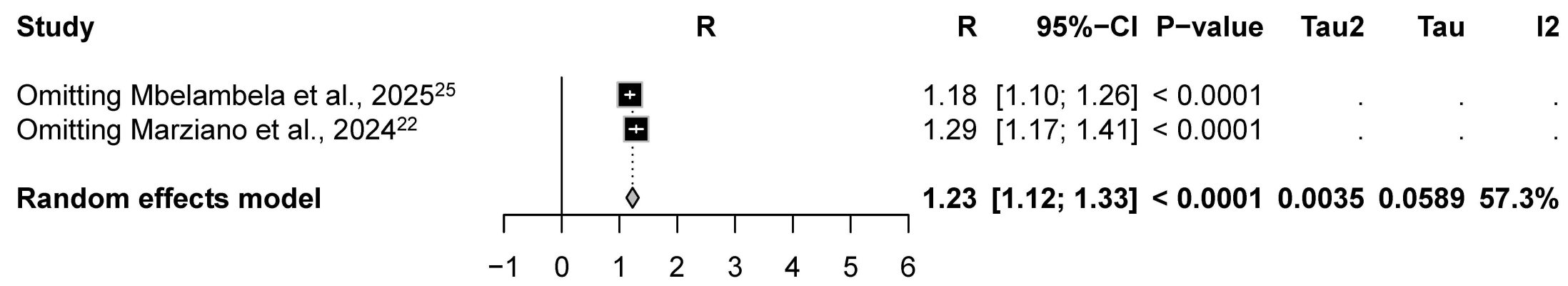

Supplemental Figure N (continued). Leave-one-out forest plot of mpox R estimates.

(iii) Mpox R estimates, within the subgroup of clade II between 2017 and 2021
The pooled R estimate in the subgroup of clade II between 2017 and 2021 ranged from 1.08 to 1.15, when each study was omitted in turn.

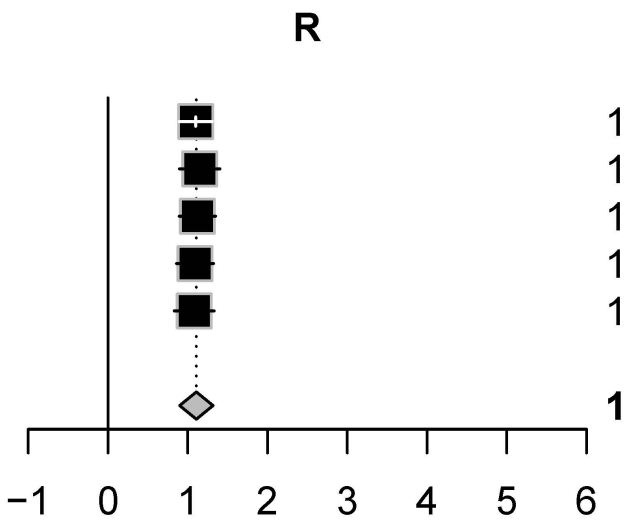

Study	R	95%−CI	P−value	Tau2	Tau	I2
Omitting Musa et al., 2022[20]	1.10	[0.89; 1.31]	< 0.0001	0	0	0.0%
Omitting Musa et al., 2022[20]	1.15	[0.90; 1.40]	< 0.0001	0	0	0.0%
Omitting Musa et al., 2022[20]	1.12	[0.89; 1.35]	< 0.0001	0	0	0.0%
Omitting Musa et al., 2022[20]	1.09	[0.86; 1.32]	< 0.0001	0	0	0.0%
Omitting Musa et al., 2022[20]	1.08	[0.83; 1.33]	< 0.0001	0	0	0.0%
**Random effects model**	**1.11**	**[0.90; 1.32]**	**< 0.0001**	**0**	**0**	**0.0%**

(iv) Mpox R estimates, within the subgroup of clade IIb during the 2022−2023 global outbreak
The pooled R estimate in the subgroup of clade IIb during the 2022−2023 global outbreak ranged from 2.54 to 2.71, when each study was omitted in turn.

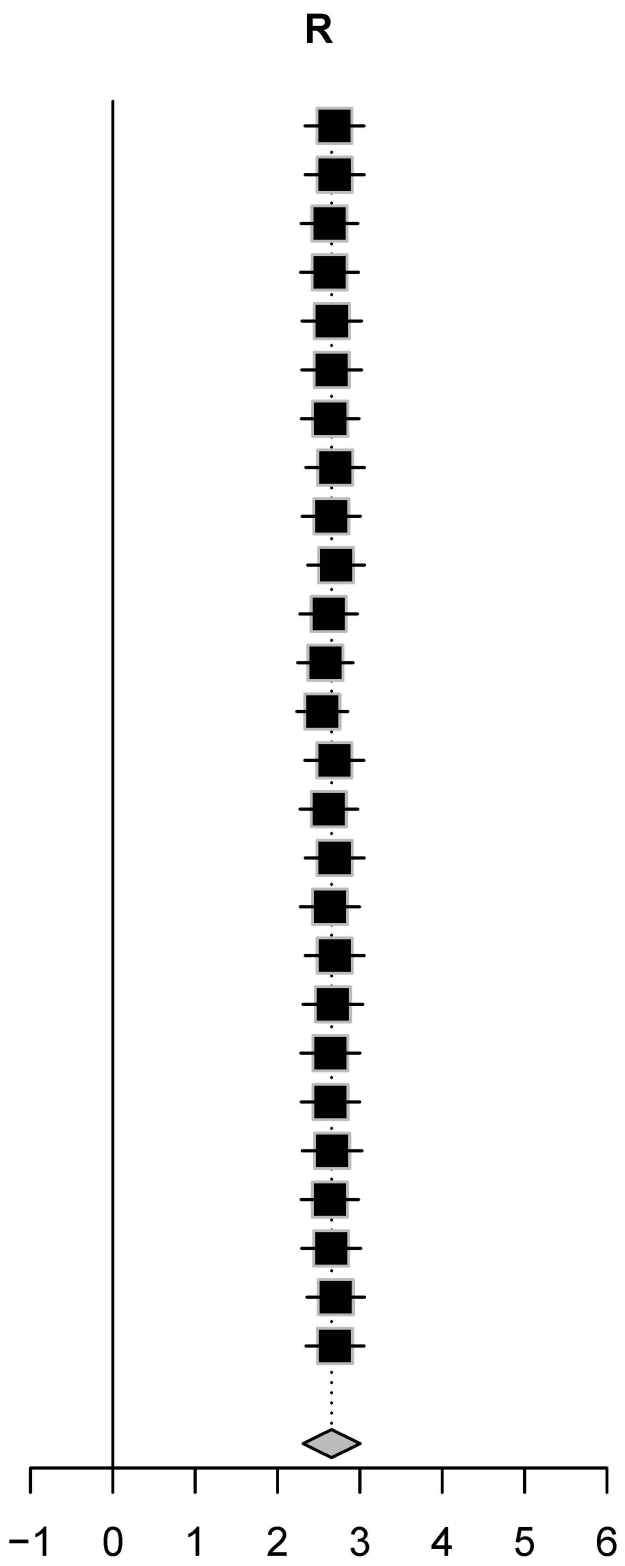

Study	R	95%−CI	P−value	Tau2	Tau	I2
Omitting Musa et al., 2022[20]	2.69	[2.33; 3.05]	< 0.0001	0.6681	0.8174	99.9%
Omitting Zhang et al., 2024[30]	2.69	[2.34; 3.05]	< 0.0001	0.6677	0.8171	99.9%
Omitting Brand et al., 2023[29]	2.63	[2.28; 2.98]	< 0.0001	0.6409	0.8005	99.9%
Omitting Yang et al., 2022[95]	2.63	[2.28; 2.98]	< 0.0001	0.6544	0.8090	99.9%
Omitting Borges et al., 2023[85]	2.66	[2.30; 3.02]	< 0.0001	0.6874	0.8291	99.9%
Omitting Cao et al., 2024[46]	2.66	[2.29; 3.02]	< 0.0001	0.6915	0.8316	99.9%
Omitting Branda et al., 2023[86]	2.64	[2.29; 2.99]	< 0.0001	0.6562	0.8101	99.9%
Omitting Kwok et al., 2022[89]	2.70	[2.34; 3.06]	< 0.0001	0.6552	0.8095	99.9%
Omitting Zygmunt et al., 2023[96]	2.65	[2.30; 3.01]	< 0.0001	0.6676	0.8171	99.9%
Omitting Bragazzi et al., 2023[45]	2.71	[2.37; 3.06]	< 0.0001	0.6138	0.7835	99.9%
Omitting Bragazzi et al., 2024[97]	2.62	[2.27; 2.97]	< 0.0001	0.6424	0.8015	99.9%
Omitting Lin et al., 2024[27]	2.58	[2.25; 2.92]	< 0.0001	0.5757	0.7588	99.7%
Omitting Augsburger et al., 2022[84]	2.54	[2.23; 2.85]	< 0.0001	0.4825	0.6946	99.9%
Omitting Guzzetta et al., 2024[34]	2.69	[2.33; 3.05]	< 0.0001	0.6710	0.8192	99.9%
Omitting Yang et al., 2023[58]	2.62	[2.27; 2.98]	< 0.0001	0.6475	0.8047	99.9%
Omitting Du et al., 2022[87]	2.69	[2.34; 3.05]	< 0.0001	0.6684	0.8175	99.9%
Omitting Zhang et al., 2025[32]	2.64	[2.28; 3.00]	< 0.0001	0.6738	0.8209	99.9%
Omitting Marín−Sánchez et al., 2023[91]	2.69	[2.34; 3.05]	< 0.0001	0.6679	0.8172	99.7%
Omitting Liao et al., 2023[90]	2.67	[2.31; 3.04]	< 0.0001	0.6917	0.8317	99.9%
Omitting Saldaña et al., 2023[93]	2.64	[2.28; 3.00]	< 0.0001	0.6762	0.8223	99.9%
Omitting Díaz−Brochero et al., 2024[98]	2.64	[2.29; 3.00]	< 0.0001	0.6689	0.8179	99.9%
Omitting Schrarstzhaupt et al., 2022[94]	2.66	[2.30; 3.03]	< 0.0001	0.6912	0.8314	99.9%
Omitting Quispe et al., 2023[92]	2.64	[2.29; 2.99]	< 0.0001	0.6518	0.8074	99.9%
Omitting Kim et al., 2024[88]	2.65	[2.29; 3.01]	< 0.0001	0.6787	0.8238	99.9%
Omitting Cordeiro et al., 2024[49]	2.71	[2.36; 3.06]	< 0.0001	0.6307	0.7942	99.9%
Omitting Zhang et al., 2024[99]	2.70	[2.34; 3.05]	< 0.0001	0.6518	0.8074	99.9%
**Random effects model**	**2.66**	**[2.31; 3.00]**	**< 0.0001**	**0.6528**	**0.8080**	**99.9%**

Supplemental Figure O. Leave-one-out forest plot of prevention percentages.

(i) Prevention percentages for vaccination (either with or without the incorporation of behaviour change)
Prevention percentages in the plots are shown in decimal form (e.g., 0.38 = 38%). Reference numbers correspond to the supplementary references list. Leave-one-out sensitivity analysis showed that the pooled prevention percentages ranged from 34% to 40% when each study was omitted in turn.

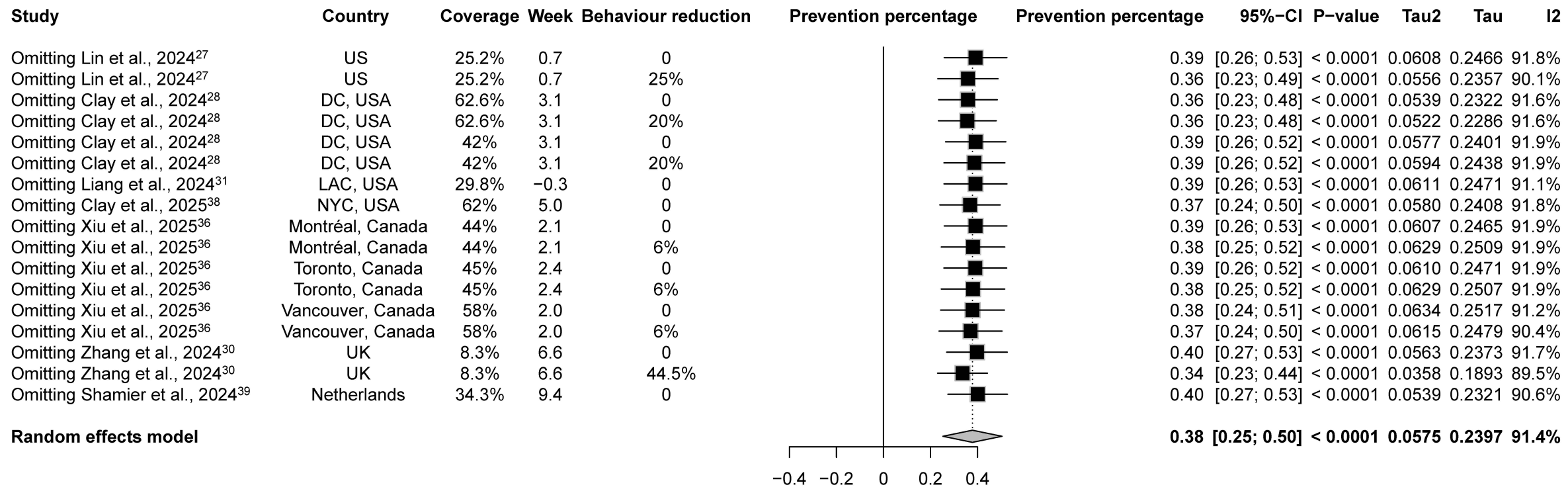

(ii) Prevention percentages for vaccination
The pooled prevention percentages for vaccination ranged from 20% to 26% when each study was omitted in turn.

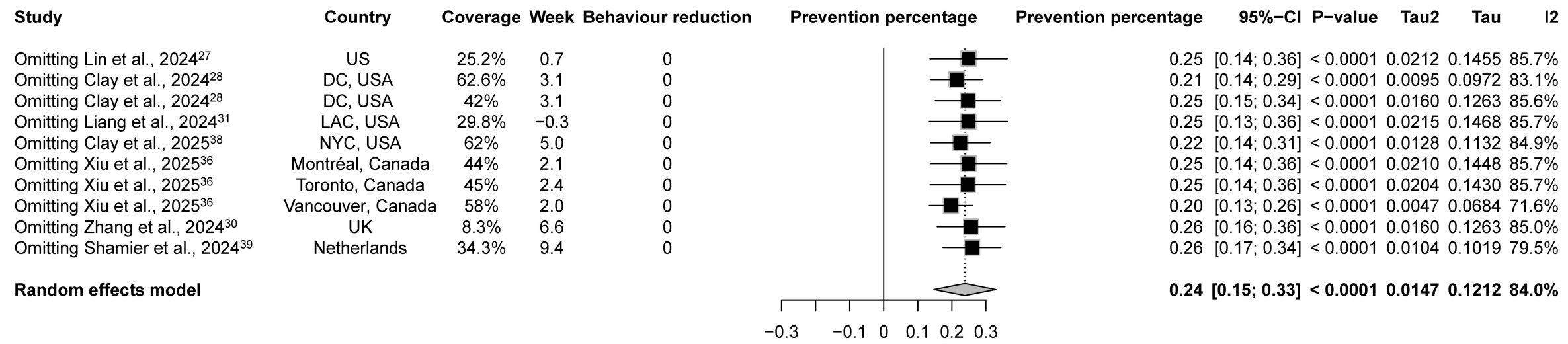

(iii) Prevention percentages for vaccination and behaviour change
The pooled prevention percentages for vaccination and behaviour change ranged from 49% to 59% when each study was omitted in turn.

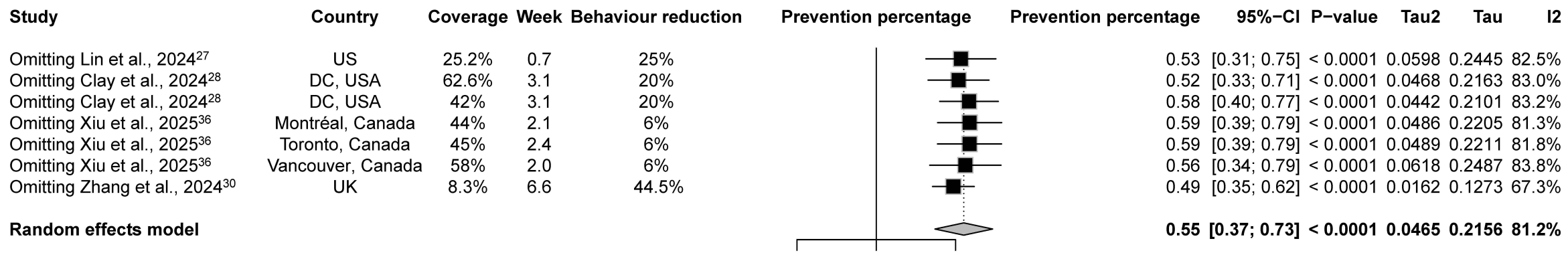

Supplemental Figure P. Meta-regression of prevention percentages for vaccination only, by first-dose coverage and weeks from first case confirmation to vaccination initiation.

Prevention percentages are shown in decimal form (e.g., 0.2 = 20%).

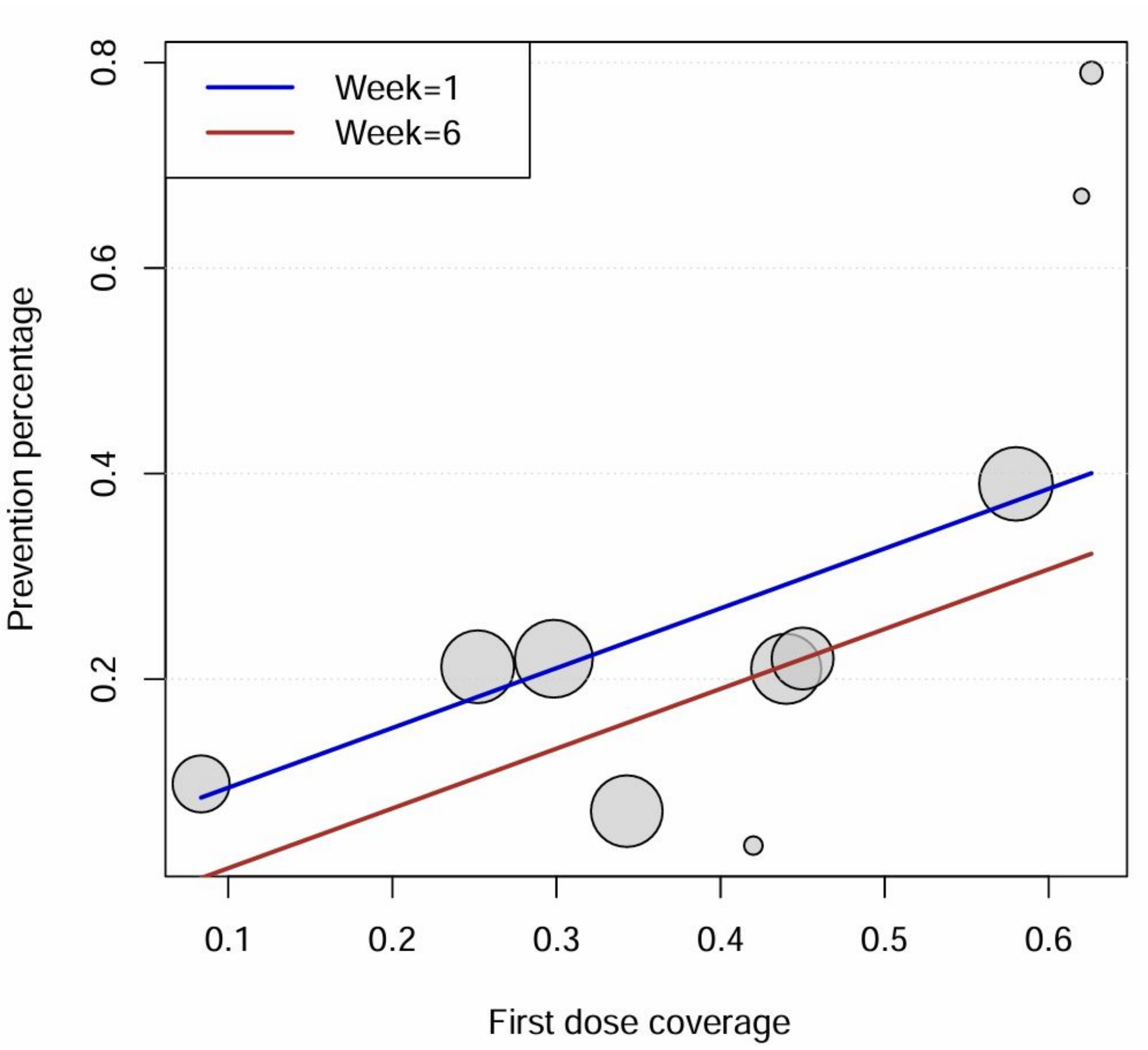

Colored lines indicate meta-regression fits stratified by weeks from first case confirmation to vaccination initiation (blue: week=1; red: week =6), plotted against varying first-dose coverage levels (x-axis). Meta-regression identified first-dose coverage (coefficient: 0.582, standard error [SE]: 0.148, 95% CI 0.293 to 0.871), and weeks from first case confirmation to vaccination initiation (coefficient: -0.0157, SE: 0.0061, 95% CI -0.0276 to -0.0037) as significant predictors in this model. The model accounted for 91.8% of the between-study heterogeneity (R^2 = 91.8%).

Supplemental Figure Q. Sensitivity phylogenetic tree generated from an independent repeat of the same stratified sampling framework as the main analysis.

The overall phylogenetic placement and clade structure remained unchanged, supporting the robustness of the main findings. OQ621553.1 exhibits a longer terminal branch, likely reflecting subsample composition (its closest neighbour in the main analysis, KC257459.1, was not sampled), which increases apparent divergence and rescales branch lengths.

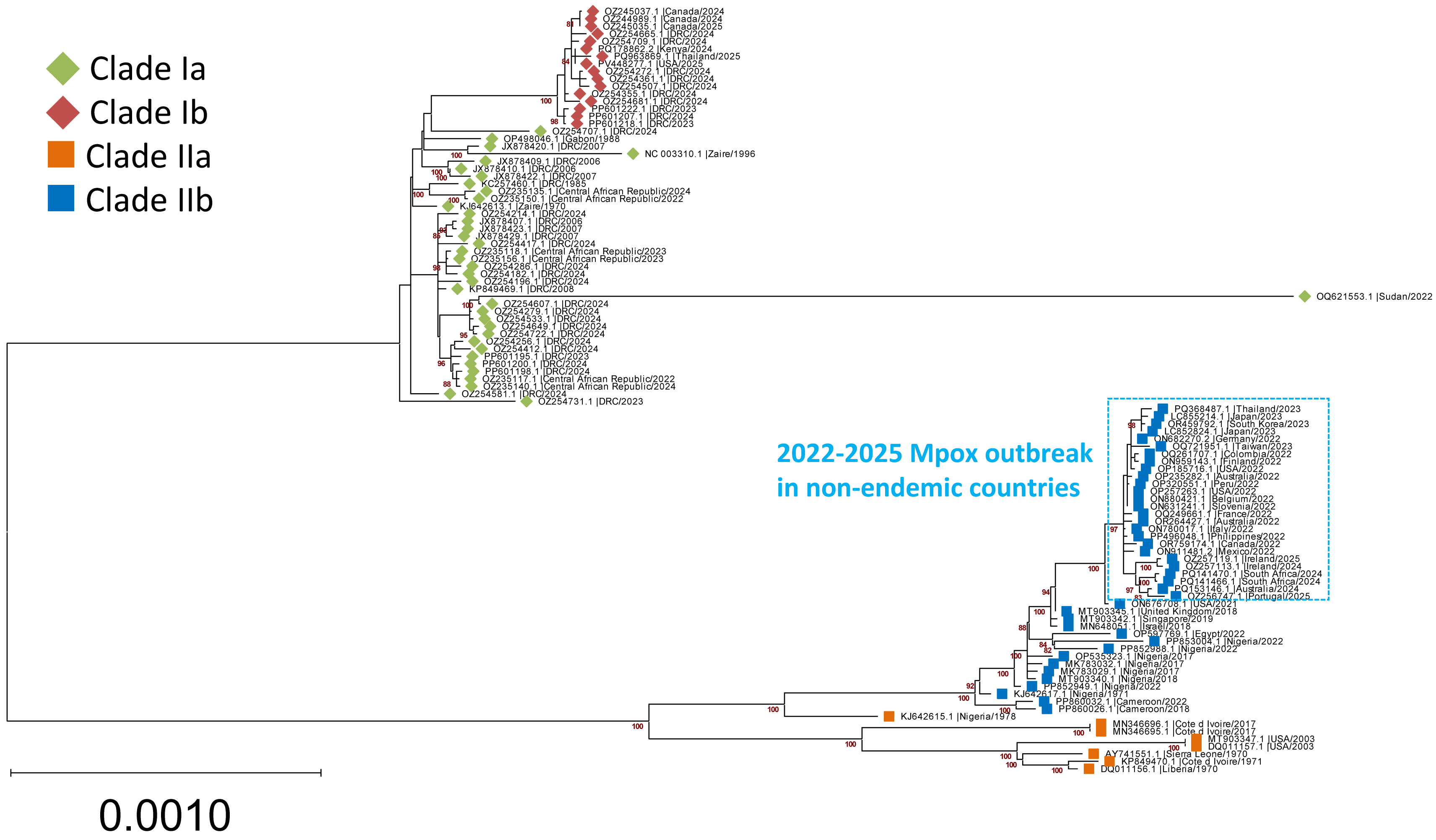

Supplemental Table A. Sequences included in phylogeny tree construction (arranged by accession number)

Accession Number	Organism	Location of Collection	Length (base pairs)	Time of Collection	Clade
AY741551.1	Monkeypox virus	Sierra Leone	198756	1970	IIa
DQ011155.1	Monkeypox virus	Zaire	196967	1978	Ia
DQ011157.1	Monkeypox virus	USA	198780	2003	IIa
HQ857562.1	Monkeypox virus	Zaire	196849	1979	Ia
JX878413.1	Monkeypox virus	Democratic Republic of the Congo	197627	2006/12/14	Ia
JX878415.1	Monkeypox virus	Democratic Republic of the Congo	197488	2006/12/26	Ia
JX878422.1	Monkeypox virus	Democratic Republic of the Congo	197346	2007/3/20	Ia
KC257459.1	Monkeypox virus	Sudan: Nuria	206372	2005	Ia
KJ642615.1	Monkeypox virus	Nigeria	197792	1978	IIa
KJ642616.1	Monkeypox virus	France: Paris	198741	1968	IIa
KJ642617.1	Monkeypox virus	Nigeria	197551	1971	IIb
KJ642618.1	Monkeypox virus	Cameroon	194363	1990	Ia
KJ642619.1	Monkeypox virus	Gabon	196546	1988	Ia
LC756924.1	Monkeypox virus	Japan: Tokyo	197141	2023/1/30	IIb
MK783031.1	Monkeypox virus	Nigeria: Rivers State	197566	2017/11/9	IIb
MN346690.1	Monkeypox virus	Cote d'Ivoire	198441	2017/3/13	IIa
MN346694.1	Monkeypox virus	Cote d'Ivoire	198406	2017/3/28	IIa
MN346698.1	Monkeypox virus	Cote d'Ivoire	198298	2017/1/24	IIa
MN648051.1	Monkeypox virus	Israel	197417	2018/10/4	IIb
MT903339.1	Monkeypox virus	Nigeria: Delta State	197556	2018	IIb
MT903342.1	Monkeypox virus	Singapore	197309	2019	IIb
MT903344.1	Monkeypox virus	United Kingdom: Great Britain	197233	2018	IIb
MT903346.1	Monkeypox virus	USA	198778	2003	IIa
NC_003310.1	Monkeypox virus	Zaire: Sankuru subregion, Kasdai Oriental	196858	1996	Ia
ON649720.1	Monkeypox virus	Portugal	197204	2022/5/19	IIb
ON676707.1	Monkeypox virus	USA: State of Texas	197265	July, 2021	IIb
ON782022.1	Monkeypox virus	Finland	197176	2022	IIb
ON792322.1	Monkeypox virus	Switzerland: Zurich	197205	2022/6/2	IIb
ON880517.1	Monkeypox virus	Canada	197195	2022/5/27	IIb
ON911481.2	Monkeypox virus	Mexico: Nuevo Leon	197413	2022/6/28	IIb
ON927248.1	Monkeypox virus	South Africa	197126	2022/6/24	IIb
OP205125.1	Monkeypox virus	United Kingdom	197205	2022/6/24	IIb
OP320548.1	Monkeypox virus	Peru	197194	2022/7/1	IIb
OP422343.1	Monkeypox virus	Austria	197204	2022/8/8	IIb
OP484678.1	Monkeypox virus	Germany	197140	September, 2022	IIb
OP498046.1	Monkeypox virus	Gabon	196640	1988	Ia
OP535333.1	Monkeypox virus	Nigeria: Oyo	197398	January, 2018	IIb
OP612682.1	Monkeypox virus	Nigeria	197052	2019/4/20	IIb
OP752119.1	Monkeypox virus	USA: State of Tennessee	197172	August, 2022	IIb
OQ621553.1	Monkeypox virus	Sudan	204808	October, 2022	Ia
OQ721920.1	Monkeypox virus	USA: State of Illinois	197155	September, 2022	IIb

OQ721953.1	Monkeypox virus	Taiwan	197129	March, 2022	IIb
OQ729808.1	Monkeypox virus	Democratic Republic of the Congo	197433	2022	Ia
OR146389.1	Monkeypox virus	Ireland	197130	2022/9/20	IIb
OR264402.1	Monkeypox virus	Australia: Victoria	197205	2022/8/22	IIb
OR264422.1	Monkeypox virus	Australia: Victoria	197188	2022/7/13	IIb
OR759200.1	Monkeypox virus	Canada: Ontario	196770	2022/7/1	IIb
OR855764.1	Monkeypox virus	South Korea	197189	2023/9/15	IIb
OZ235118.1	Monkeypox virus	Central African Republic: Bangui; Bimbo	190429	2023/9/15	Ia
OZ235136.1	Monkeypox virus	Central African Republic: Lobaye; Mbaiki	189522	2024/8/11	Ia
OZ235145.1	Monkeypox virus	Central African Republic: Ouaka; Bambari	190459	2024/3/28	Ia
OZ235149.1	Monkeypox virus	Central African Republic: Sangha-Mbaere; Bayanga	190420	2022/10/12	Ia
OZ245035.1	Monkeypox virus	Canada	195413	January, 2025	Ib
OZ245036.1	Monkeypox virus	Canada: Manitoba	195458	2024/11/21	Ib
OZ246959.1	Monkeypox virus	Slovakia	192382	2025/1/13	IIb
OZ254230.1	Monkeypox virus	Democratic Republic of the Congo: Tshopo	196459	2024/8/30	Ia
OZ254289.1	Monkeypox virus	Democratic Republic of the Congo: Kwilu	196056	2024/8/19	Ia
OZ254302.1	Monkeypox virus	Democratic Republic of the Congo: Kasai	195970	2024/8/15	Ib
OZ254338.1	Monkeypox virus	Democratic Republic of the Congo: Sud-Ubangi	196884	2024/7/1	Ia
OZ254371.1	Monkeypox virus	Democratic Republic of the Congo: Nord-Ubangi	196439	2024/8/20	Ia
OZ254377.1	Monkeypox virus	Democratic Republic of the Congo: Kongo-central	196416	2024/8/30	Ia
OZ254431.1	Monkeypox virus	Democratic Republic of the Congo: Maindombe	196812	2024/7/23	Ia
OZ254448.1	Monkeypox virus	Democratic Republic of the Congo: Tshopo	196388	2024/9/8	Ib
OZ254454.1	Monkeypox virus	Democratic Republic of the Congo: Mongala	195230	2024/3/14	Ia
OZ254462.1	Monkeypox virus	Democratic Republic of the Congo: Tshuapa	196685	2017/12/29	Ia
OZ254507.1	Monkeypox virus	Democratic Republic of the Congo: Tanganyika	196409	2024/9/16	Ib
OZ254561.1	Monkeypox virus	Democratic Republic of the Congo: Sankuru	195959	2024/8/14	Ia
OZ254579.1	Monkeypox virus	Democratic Republic of the Congo: Equateur	195902	2024/3/17	Ia
OZ254582.1	Monkeypox virus	Democratic Republic of the Congo: Haut-Uele	196260	2024/10/22	Ia
OZ254613.1	Monkeypox virus	Democratic Republic of the Congo: Kinshasa	196470	2024/11/5	Ia
OZ254664.1	Monkeypox virus	Democratic Republic of the Congo: Bas-Uele	196488	2024/10/2	Ia
OZ254697.1	Monkeypox virus	Democratic Republic of the Congo: Sud-Kivu	196888	2024/5/14	Ib
OZ254705.1	Monkeypox virus	Democratic Republic of the Congo: Kinshasa	195184	2024/11/7	Ib
OZ254731.1	Monkeypox virus	Democratic Republic of the Congo: Sud-Kivu	195965	2023/11/17	Ia
OZ254775.1	Monkeypox virus	Democratic Republic of the Congo: Nord-Kivu	195141	2024/8/6	Ib
OZ254823.1	Monkeypox virus	Democratic Republic of the Congo: Sud-Ubangi	196528	2020/2/15	Ia
OZ254852.1	Monkeypox virus	Democratic Republic of the Congo: Tshuapa	196384	2024/9/7	Ia
OZ256743.1	Monkeypox virus	Portugal	197214	2025/3/20	IIb
PP601189.1	Monkeypox virus	Democratic Republic of the Congo: Sud-Ubangi, Bulu	196784	2023/12/18	Ia
PP601193.1	Monkeypox virus	Democratic Republic of the Congo: Tshopo, Yahuma	196822	2023/12/27	Ia
PP601197.1	Monkeypox virus	Democratic Republic of the Congo: Sud-Ubangi, Bulu	196807	2024/1/7	Ia
PP601199.1	Monkeypox virus	Democratic Republic of the Congo: Tshopo, Basoko	196801	2024/1/13	Ia
PP601206.1	Monkeypox virus	Democratic Republic of the Congo: Equateur, Wangata	196806	2024/1/20	Ia
PP601216.1	Monkeypox virus	Democratic Republic of the Congo: Sud-Kivu, Kamituga	195613	2024/1/26	Ib
PP601220.1	Monkeypox virus	Democratic Republic of the Congo: Sud-Kivu, Kamituga	195580	2023/12/26	Ib
PP601225.1	Monkeypox virus	Democratic Republic of the Congo: Sud-Kivu, Kamituga	195600	2024/1/14	Ib
PP601228.1	Monkeypox virus	Democratic Republic of the Congo: Sud-Kivu, Kamituga	195596	2023/10/13	Ib

PP852959.1	Monkeypox virus	Nigeria: AkwaIbom	197194	2023/2/21	IIb
PP853014.1	Monkeypox virus	Nigeria: Rivers	196917	2022/10/3	IIb
PP853018.1	Monkeypox virus	Nigeria: Bayelsa	197101	2022/10/30	IIb
PP853019.1	Monkeypox virus	Nigeria: Lagos	197088	2022/9/14	IIb
PP860027.1	Monkeypox virus	Cameroon: South-west	197073	2022/10/13	IIb
PP860031.1	Monkeypox virus	Cameroon: Centre	197086	2021/7/22	IIb
PQ141467.1	Monkeypox virus	South Africa: Kwa-Zulu Natal	197171	2024/5/31	IIb
PQ153167.1	Monkeypox virus	Australia: Victoria	197195	2024/6/17	IIb
PQ178862.2	Monkeypox virus	Kenya: Taita Taveta	195321	2024/7/25	Ib
PQ213333.1	Monkeypox virus	South Africa: Gauteng	197159	2024/6/28	IIb
PQ240043.1	Monkeypox virus	Thailand	195599	2024/8/16	Ib
PQ368491.1	Monkeypox virus	Thailand	197181	2023/12/1	IIb
PV002568.1	Monkeypox virus	USA: State of Georgia	195536	January, 2025	Ib
PV031939.1	Monkeypox virus	USA: Washington	197145	2024/11/19	IIb
PV448277.1	Monkeypox virus	USA: State of New York	195403	February, 2025	Ib

Supplemental Table B. Information on studies excluded by full-text reading, for the review on estimates of mpox reproduction number

Study	Title	Reason for Exclusion
Sypsa et al., 2022	Transmission Potential of Human Monkeypox in Mass Gatherings	Hypothetical mathematic simulations of R
Case et al., 2023	Accurately summarizing an outbreak using epidemiological models takes time	Unrelated to mpox
Kollepara et al., 2023	Heterogeneity in network structure switches the dominant transmission mode of infectious diseases	Hypothetical mathematic simulations of R
Branda et al., 2022	Monkeypox: EpiMPX Surveillance System and Open Data with a Special Focus on European and Italian Epidemic	Performed mpox R estimation, but no specific reported values of R available
Endo et al., 2022	Heavy-tailed sexual contact networks and monkeypox epidemiology in the global outbreak, 2022	No reported values of R available
Savinkina et al., 2023	Planning for Mpox on a College Campus: A Model-Based Decision-Support Tool	Simulations of R under hypothetical setting
El-Mesady et al., 2022	On nonlinear dynamics of a fractional order monkeypox virus model	Hypothetical mathematic simulations of R
Okoli et al., 2023	A global systematic evidence review with meta-analysis of the epidemiological characteristics of the 2022 Mpox outbreaks	Review
Banuet-Martinez et al., 2023	Monkeypox: a review of epidemiological modelling studies and how modelling has led to mechanistic insight	Review
Okyere et al., 2023	Modeling and analysis of monkeypox disease using fractional derivatives	Hypothetical mathematic simulations of R
Chitwood et al., 2023	Estimated Testing, Tracing, and Vaccination Targets for Containment of the US Mpox Outbreak	R0 used as an assumed, known parameter
Yuan et al., 2023	Assessing transmission risks and control strategy for monkeypox as an emerging zoonosis in a metropolitan area	R0 used as an assumed, known parameter
Asif et al., 2023	Metaheuristics optimization-based ensemble of deep neural networks for Mpox disease detection	Proposed models for Mpox disease detection
Fernandes et al., 2023	Behavioral aspects and the transmission of Monkeypox: A novel approach to determine the probability of transmission for sexually transmissible diseases	No reported values of R available
Peter et al., 2022	Transmission dynamics of Monkeypox virus: a mathematical modelling approach	Hypothetical mathematic simulations of R
Singh et al., 2023	Modeling Global Monkeypox Infection Spread Data: A Comparative Study of Time Series Regression and Machine Learning Models	Proposed models for forecasts of mpox outbreak, no reported values of R available
Haider et al., 2022	Increased outbreaks of monkeypox highlight gaps in actual disease burden in Sub-Saharan Africa and in animal reservoirs	Focus on disease burden
Jia et al., 2023	Genomic and epidemiological perspectives on the first local sporadic cases of Mpox in China	Genomic or molecular study
Khan et al., 2022	Stochastic modeling of the Monkeypox 2022 epidemic with cross-infection hypothesis in a highly disturbed environment	Hypothetical mathematic simulations of R
Yuan et al., 2022	Modeling vaccination and control strategies for outbreaks of monkeypox at gatherings	R0 used as an assumed, known parameter
Li et al., 2022	[Perspectives on recent monkeypox outbreak in non-endemic areas]	Review
Chavda et al., 2023	Omicron Variant of SARS-CoV-2: An Indian Perspective of Vaccination and Management	Unrelated to mpox
Eggers et al., 2022	Hygiene and disinfection measures for monkeypox virus infections	Focus on strategies of mpox virus disinfection and hygiene
Anoop et al., 2023	Public discourse and sentiment during Mpox outbreak: an analysis using natural language processing	Qualitative study
Li et al., 2023	Modelling the impact of timelines of testing and isolation on disease control	R0 used as an assumed, known parameter
Knight et al., 2022	Maximizing the impact of limited vaccine supply under different early epidemic conditions: a 2-city	R0 used as an assumed, known parameter

	modelling analysis of monkeypox virus transmission among men who have sex with men	
Weber et al., 2023	Emerging infectious diseases, focus on infection prevention, environmental survival and germicide susceptibility: SARS-CoV-2, Mpox, and Candida auris	Review
Van Dijck et al., 2023	Emergence of mpox in the post-smallpox era-a narrative review on mpox epidemiology	Review
Martínez-Fernández et al., 2023	Human Monkeypox: A Comprehensive Overview of Epidemiology, Pathogenesis, Diagnosis, Treatment, and Prevention Strategies	Review
Brüssow H., 2023	Pandemic potential of poxviruses: From an ancient killer causing smallpox to the surge of monkeypox	Review
Khamees et al., 2023	Human monkeypox virus in the shadow of the COVID-19 pandemic	Review
Pinto et al., 2023	Mpox Person-to-Person Transmission-Where Have We Got So Far? A Systematic Review	Review
Gurnani et al., 2023	Ophthalmic manifestations of monkeypox infection	Review
Hamdana et al., 2023	Monkeypox Virus and Other Emerging Outbreaks: An Overview and Future Perspective	Review
Sharma et al., 2023	Emerging evidence on Monkeypox: resurgence, global burden, molecular insights, genomics and possible management	Review
Molla et al., 2023	Mathematical modeling of mpox: A scoping review	Review
Sharma et al., 2023	Monkeypox epidemiology, clinical presentation, and transmission: a systematic review	Review
Islam et al., 2023	Pathogenicity and virulence of monkeypox at the human-animal-ecology interface	Review
Ghate et al., 2023	Molecular detection of monkeypox and related viruses: challenges and opportunities	Review
Pan et al., 2023	Transmission of monkeypox/mpox virus: A narrative review of environmental, viral, host, and population factors in relation to the 2022 international outbreak	Review
Kumar et al., 2023	As the World Struggles With the COVID-19 Pandemic, Another Emergency Threat Arrives on the Horizon, the Monkeypox: A Systematic Review	Review
Zardi et al., 2022	Human Monkeypox-A Global Public Health Emergency	Review
Chen et al., 2022	Epidemiology-based analysis of the risks and elimination strategies of the monkeypox outbreak in 2022	Review
Singh et al., 2022	All That We Need to Know About the Current and Past Outbreaks of Monkeypox: A Narrative Review	Review
Farahat et al., 2022	Asymptomatic transmission of monkeypox: Implications for mass gatherings?	Review
Jiang et al., 2023	Diagnosis, treatment, and prevention of monkeypox in children: an experts' consensus statement	Review
Liang et al., 2022	Biological characteristics, biosafety prevention and control strategies for the 2022 multi-country outbreak of monkeypox	Review
Farah et al., 2023	A review of monkeypox infection during pregnancy	Review
Elsayed et al., 2022	Monkeypox Virus Infections in Humans	Review
DeWitt et al., 2022	Global monkeypox case hospitalisation rates: A rapid systematic review and meta-analysis	Review
Zimmermann et al., 2022	Monkeypox-What Pediatricians Need to Know	Review
Huang et al., 2022	Monkeypox: epidemiology, pathogenesis, treatment and prevention	Review
Li et al., 2022	The evolving epidemiology of monkeypox virus	Review
Obeid et al., 2022	Monkeypox: Emerging virus of concern; antivirals and vaccines therapeutic options	Review
Farahat et al., 2022	Human monkeypox disease (MPX)	Review
Riopelle et al., 2022	Atypical and Unique Transmission of Monkeypox Virus during the 2022 Outbreak: An Overview of the Current State of Knowledge	Review
Gomez-Lucia E., 2022	Monkeypox: Some Keys to Understand This Emerging Disease	Review
Kumbhar et al., 2022	The lurking threat of monkeypox in current times	Review
Dashraath et al., 2022	Monkeypox in pregnancy: virology, clinical presentation, and obstetric management	Review
Kaler et al., 2022	Monkeypox: A Comprehensive Review of Transmission, Pathogenesis, and Manifestation	Review
Tiecco et al., 2022	Omicron BA.2 Lineage, the "Stealth" Variant: Is It Truly a Silent Epidemic? A Literature Review	Review
Kumar et al., 2022	The 2022 outbreak and the pathobiology of the monkeypox virus	Review
Mileto et al., 2022	New challenges in human monkeypox outside Africa: A review and case report from Italy	Review

Feng et al., 2023	Unexpectedly higher levels of anti-orthopoxvirus neutralizing antibodies are observed among gay men than general adult population	Focus on virology
Kröger et al., 2023	Mpox outbreak 2022: an overview of all cases reported to the Cologne Health Department	Case series and epidemiology study
Peter et al., 2022	Transmission dynamics of Monkeypox virus: a mathematical modelling approach	Hypothetical mathematic simulations, no reported values of R available
Wilder-Smith A., 2021	COVID-19 in comparison with other emerging viral diseases: risk of geographic spread via travel	Unrelated to mpox
Mohindra et al., 2021	Superspreaders: A Lurking Danger in the Community	Case report
Alakunle et al., 2020	Monkeypox Virus in Nigeria: Infection Biology, Epidemiology, and Evolution	Review
Devaux et al., 2019	Infectious Disease Risk Across the Growing Human-Non Human Primate Interface: A Review of the Evidence	Review
Beer et al., 2019	A systematic review of the epidemiology of human monkeypox outbreaks and implications for outbreak strategy	Review
Lloyd-Smith JO., 2013	Vacated niches, competitive release and the community ecology of pathogen eradication	Review
Reynolds et al., 2012	Factors affecting the likelihood of monkeypox's emergence and spread in the post-smallpox era	Review
Learned et al., 2005	Extended interhuman transmission of monkeypox in a hospital community in the Republic of the Congo, 2003	Case report
Antia et al., 2003	The role of evolution in the emergence of infectious diseases	No reported values of R available
Pattyn SR., 2000	Monkeypox virus infections	Review
Nguyen et al., 2023	Early administration of tecovirimat shortens the time to mpox clearance in a model of human infection	Estimated within-host basic reproduction number of mpox in non-human population setting
Laurenson-Schafer et al., 2023	Description of the first global outbreak of mpox: an analysis of global surveillance data	Performed mpox R estimation, but no specific reported values of R available
García-García et al., 2022	Estimates of mpox effective reproduction number in Spain, April–August 2022	Performed mpox R estimation, but no specific reported values of R available
Zheng et al., 2023	Disparities in transmission dynamics of the 2022 mpox outbreaks between Europe and Americas	Performed mpox R estimation, but no specific reported values of R available
Ngungu et al., 2023	Mathematical epidemiological modeling and analysis of monkeypox dynamism with nonpharmaceutical intervention using real data from United Kingdom	Performed mpox R estimation, but no specific reported values of R available
Addai et al., 2023	Modelling the impact of vaccination and environmental transmission on the dynamics of monkeypox virus under Caputo operator	Hypothetical mathematic simulations of R
Adom-Konadu et al., 2023	A fractional order Monkeypox model with protected travelers using the fixed point theorem and Newton polynomial interpolation	Hypothetical mathematic simulations of R
Majee et al., 2023	Dynamical analysis of monkeypox transmission incorporating optimal vaccination and treatment with cost-effectiveness	Hypothetical mathematic simulations of R
Abedien et al., 2022	Monkeypox and Its Recent OUTBREAKS; A Systemic Review	Review
Abu-Azzam et al., 2024	Monkeypox infection and pregnancy in lower and middle-income countries: Precautions & recommendations	Review
Adegboye et al., 2024	Monkeypox Outbreak 2022, from a Rare Disease to Global Health Emergence: Implications for Travellers	Review
Al-Eitan et al., 2024	Poxviruses from the Concept of One Health	Review
Al-Tawfiq et al., 2024	Mpox outbreak in South Africa: A wake-up call for strengthening preparedness and response	Editorial, focusing on the response to mpox outbreak, did not perform mpox R estimation
Alakunle et al., 2024	A comprehensive review of monkeypox virus and mpox characteristics	Review
Alfaro et al., 2023	Global Mpox epidemic and its impact on adolescents	Review
Alhalaseh et al., 2024	Mpox Case Presenting With Genital Lesions and Proctitis	Case report

Bankuru et al., 2020	A game-theoretic model of Monkeypox to assess vaccination strategies	Hypothetical mathematic simulations of R
Bhunu et al., 2012	Modelling HIV/AIDS and monkeypox co-infection	Hypothetical mathematic simulations of R
Brasil et al., 2022	What do we need to know about the monkeypox virus infection in humans?	Review
Cheema et al., 2023	Trends in Monkeypox transmission: Investigation into 30 most affected countries	Focus on modelling disease burden, no R estimates available
Cieslak et al., 2019	A methodology for determining which diseases warrant care in a high-level containment care unit	Review
Haridas K Das, 2024	Exploring the dynamics of monkeypox transmission with data-driven methods and a deterministic model	Construct mpox dynamic modellings and performed mpox R estimation, but no specific reported values of R available
Deng et al., 2024	The risk of mpox importation and subsequent outbreak potential in Chinese mainland: a retrospective statistical modelling study	R0 used as an assumed, known parameter
Firoz et al., 2022	Monkeypox: A comprehensive review on transmission, clinical features and management of human monkeypox	Review
Ghazy et al., 2023	How can imported monkeypox break the borders? A rapid systematic review	Review
Gressani et al., 2024	Flexible Bayesian estimation of incubation times	Focus on models for incubation period distribution, no R estimates available
Halton et al., 2013	A systematic review of community-based interventions for emerging zoonotic infectious diseases in Southeast Asia	Review
Johnson et al., 2022	Evolutionary consequences of delaying intervention for monkeypox	Correspondence, did not perform mpox R estimation
Joshi et al., 2022	Human-to-Human Transmission of Monkeypox Virus Old Virus with a New Face	Review
Eric Ka-Wai Hui, 2006	Reasons for the increase in emerging and re-emerging viral infectious diseases	Review
Temi Lampejo, 2023	Risks of monkeypox virus infection in children	Letter to the editor, did not perform mpox R estimation
Lloyd-Smith et al., 2009	Epidemic dynamics at the human-animal interface	Review
McGrail et al., 2024	Comparative Analysis of 2022 Outbreak MPXV and Previous Clade II MPXV	Focus on virology
McMullen et al., 2015	Human monkeypox transmission dynamics thirty years after smallpox eradication in the Sankuru district, democratic republic of Congo	Performed mpox R estimation, but only abstract available
Ng et al., 2023	Monkeypox: A review of data essential in primary care	Review
Prompetchara et al., 2024	Mpox global health emergency: Insights into the virus, immune responses, and advancements in vaccines PART I: Insights into the virus and immune responses	Review
Protopapas et al., 2024	Mpox and Lessons Learned in the Light of the Recent Outbreak: A Narrative Review	Review
Quarleri et al., 2022	Monkeypox: considerations for the understanding and containment of the current outbreak in non-endemic countries	Review
Rehman et al., 2024	An updated review of human monkeypox disease: A new potential global hazard	Review
Rezza et al., 2019	Emergence of human monkeypox in west Africa	Comment, did not perform mpox R estimation
Rohaim et al., 2024	Molecular Virology of Orthopoxviruses with Special Reference to Monkeypox Virus	Review
Safdar et al., 2023	The global spread of human monkeypox virus: Challenges and opportunities for prevention	Review
Saksena et al., 2023	SARS-CoV-2 variants, its recombinants and epigenomic exploitation of host defenses	Unrelated to mpox
Salomon et al., 2024	Monkeypox Outbreak in the Democratic Republic of Congo: A Comprehensive Review of Clinical Outcomes, Public Health Implications, and Security Measures	Review
Schneider et al., 2022	Does it matter who is spreading monkeypox?	Comment, did not perform mpox R estimation
Shanmugam et al., 2024	Comprehending symmetry in epidemiology: A review of analytical methods and insights from models	Review

	of COVID-19, Ebola, Dengue, and Monkeypox	
Jonathan Smith, 2022	Yes, it matters who is spreading monkeypox	Correspondence, did not perform mpox R estimation
Son et al., 2024	A Multi-pronged Approach to Addressing Global Poxviruses Vaccine Inequity: A Case of Monkeypox	Review
Srivastava et al., 2023	The Global Monkeypox (Mpox) Outbreak: A Comprehensive Review	Review
Sultana et al., 2024	Monkeypox: In Quest of Effective Vaccines	Review
Tang et al., 2023	Human mpox: Biology, epidemiology, therapeutic options, and development of small molecule inhibitors	Review
The Lancet Digital Health., 2022	Crowdsourced predictions for emerging infectious diseases	Editorial, did not perform mpox R estimation
Tiecco et al., 2022	Monkeypox, a Literature Review: What Is New and Where Does This concerning Virus Come From?	Review
Toumasis et al., 2022	A comprehensive review of monkeypox (mpox) on the occasion of the ongoing multicountry outbreak of the disease	Review
Wolfe et al., 2005	Bushmeat hunting, deforestation, and prediction of zoonotic disease emergence	Review
Idisi et al., 2023	A new compartmentalized epidemic model to analytically study the impact of awareness on the control and mitigation of the monkeypox disease	Construct mpox dynamic modellings but no R estimates available
Khan et al., 2024	The transmission dynamics of an infectious disease model in fractional derivative with vaccination under real data	Mathematic simulations of R under analytical scenarios
Ko et al., 2023	Estimation of monkeypox spread in a nonendemic country considering contact tracing and self-reporting: A stochastic modeling study	Hypothetical mathematic modelling simulations, no R estimates available
Leandry et al., 2023	An investigation on the Monkeypox virus dynamics in human and rodent populations for a deterministic mathematical model	Hypothetical mathematic modelling simulations
Madewell et al., 2023	Serial Interval and Incubation Period Estimates of Monkeypox Virus Infection in 12 Jurisdictions, United States, May-August 2022	Did not perform mpox R estimation
McQuiston et al., 2025	The Rise of Mpox in a Post-Smallpox World	Review
Peace et al., 2024	A compartmental deterministic epidemiological model with non-linear differential equations for analyzing the co-infection dynamics between COVID-19, HIV, and Monkeypox diseases	Performed mpox R estimation, but no specific reported values of R available
Omame et al., 2024	Understanding the impact of HIV on mpox transmission in the MSM population: A mathematical modeling study	Hypothetical mathematic simulations of R
Shamier et al., 2024	Scenarios of future mpox outbreaks among men who have sex with men: a modelling study based on cross-sectional seroprevalence data from the Netherlands, 2022	Construct mpox dynamic modellings, but no R estimates available
Ullah et al., 2024	Behavioral game of quarantine during the monkeypox epidemic: Analysis of deterministic and fractional order approach	Hypothetical mathematic simulations of R
Xiong et al., 2024	Assessing vaccine strategies for mpox outbreak in New York City using an age-structure model	Performed mpox R estimation and plotted the Re curve through time, but no specific reported values of R available
Xu et al., 2024	From COVID-19 to monkeypox: a novel predictive model for emerging infectious diseases	No specific reported values of R estimates available
Adepoju et al., 2024	An optimal control model for monkeypox transmission dynamics with vaccination and immunity loss following recovery	Hypothetical mathematic simulations of R, no specific reported values of mpox R available
Akinyemi et al., 2023	A tale of two countries: Optimal control and cost-effectiveness analysis of monkeypox disease in Germany and Nigeria	No specific reported values of R available
Dayan et al., 2024	Theoretical Analysis and Numerical Simulation of the Transmission Dynamics of Monkeypox Virus: A Common Disease in Central and West Africa	Hypothetical mathematic simulations of R, no specific reported values of mpox R available
Sankar et al., 2025	Mpox Virus as a Global Public Health Emergency: A Scoping Review	Review

Xiu et al., 2025	Impact of interventions on mpox transmission during the 2022 outbreak in Canada: a mathematical modeling study of three different cities	Construct mpox dynamic modellings, but no specific reported values of R available
Wallinga et al., 2025	Dynamic Landscape of Mpox Importation Risks Driven by Heavy-Tailed Sexual Contact Networks Among Men Who Have Sex With Men in 2022	Performed mpox R estimation and plotted the Rt curve through time, but no specific reported values of R available
Acharya et al., 2025	"Mpox in MSM: Tackling stigma, minimizing risk factors, exploring pathogenesis, and treatment approaches"	Review
Beiras et al., 2025	Concurrent outbreaks of mpox in Africa-an update	Review
Diaz et al., 2025	Decoding mpox: A systematic review and meta-Analysis of the transmission and severity parameters of the 2022-2023 global outbreak	Review
Eslamkhah et al., 2025	Mpox virus (MPXV): comprehensive analysis of pandemic risks, pathophysiology, treatments, and mRNA vaccine development	Review
McGrail et al., 2024	Comparative Analysis of 2022 Outbreak MPXV and Previous Clade II MPXV	Did not perform mpox R estimation
Petersen et al., 2025	Possible scenarios for the spread of mpox outside the endemic focus in Africa	Did not perform mpox R estimation
Rashid et al., 2025	Dynamic analysis and optimal control of a hybrid fractional monkeypox disease model in terms of external factors	Hypothetical mathematic simulations of R
Yadav et al., 2024	Exploring Monkeypox: An In-depth Examination of Its History, Current Status, and Prospects	Review
Blumberg et al., 2014	Detecting differential transmissibilities that affect the size of self-limited outbreaks.	Perform mpox transmission model but the reported mpox R had already been published in Blumberg et al., 2013[15]
Qian et al., 2025	An epidemiological model of monkeypox: model prediction and control application	Hypothetical mathematic simulations of R
Ahmed et al., 2025	The emergence of a novel mpox virus strain (clade Ib) in Central Africa: A global public health concern	Short communication report, did not perform mpox R estimation
Brüssow et al., 2025	Monkeypox Virus: WHO's Second Public Health Emergency of International Concern Within 2 Years	Review
Okoli et al., 2025	Epidemiological characteristics of the 2022 mpox outbreaks: a comprehensive global systematic evidence review with meta-analysis	Review
Idisi et al., 2025	Investigating Mpox Strain Dynamics Using Computational and Data-Driven Approaches	Hypothetical mathematic simulations of R
Omame et al., 2025	Dynamics of Mpox in an HIV endemic community: A mathematical modelling approach	Hypothetical mathematic simulations of R
Sankar et al., 2025	Mpox Virus as a Global Public Health Emergency: A Scoping Review	Review
Pekar et al., 2025	Transmission dynamics of the 2022 mpox epidemic in New York City	Performed mpox R estimation and plotted the Re curve through time, but no specific reported values of R available
Pekar et al., 2025	Transmission through a heavy-tailed sexual contact network explains the New York City mpox outbreak	Research Briefing of another excluded article: Pekar et al. Transmission dynamics of the 2022 mpox epidemic in New York City. Nat Med. 2025 May;31(5):1464-1473.
Musafir et al., 2025	Dynamics and optimal control of fractional-order monkeypox epidemic model with social distancing habits and public awareness	Hypothetical mathematic simulations of R
Kandi, V., 2024	Will mpox disease caused by the human Mpox virus (MPXV) result in a pandemic-like situation similar to the COVID-19 pandemic	Review
Yu et al., 2024	Epidemiology and research progress in prevention and control of mpox	Review
Hashmi et al., 2025	Emergence of mpox in Oman: investigating the first cluster and its implications, October 2023	Case report

Supplemental Table C. Information on studies excluded by full-text reading, for the review on the effects of mpox mitigation measures assessed by transmission dynamic model

Study	Title	Reason for Exclusion
Evers et al., 2023	Using a theoretical framework of Intervention Mapping to inform public health communication messages designed to increase vaccination uptake; the example of mpox in the Netherlands	Focus on forming communication strategies through model framework
Ramchandani et al., 2023	Effectiveness of the Modified Vaccinia Ankara Vaccine Against Mpox in Men Who Have Sex With Men: A Retrospective Cohort Analysis, Seattle, Washington	Focus on estimation of vaccine effectiveness
Wolff Sagy et al., 2023	Real-world effectiveness of a single dose of mpox vaccine in males	Focus on estimation of vaccine effectiveness
Piccolo et al., 2023	Critical Elements of an Mpox Vaccination Model at the Largest Public Health Hospital System in the United States	Focus on empirical policy in hospitals
Branda et al., 2023	Monkeypox: Early estimation of basic reproduction number R(0) in Europe	Focus on estimation of epidemiological parameters
Vairo et al., 2023	The possible effect of sociobehavioral factors and public health actions on the mpox epidemic slowdown	Ineligible model type (regression model)
Gao et al., 2023	Driving effect of multiplex factors on Mpox in global high-risk region, implication for Mpox based on one health concept	Model for other purposes, ineligible model type (ecological niche modelling)
McFarland et al., 2023	Estimated incubation period distributions of mpox using cases from two international European festivals and outbreaks in a club in Berlin, May to June 2022	Focus on estimation of incubation period
Gao et al., 2024	Global transboundary transmission path and risk of Mpox revealed with Least Cost Path model	ineligible model type (spatial model)
Quispe et al., 2023	Monkeypox in Latin America and the Caribbean: assessment of the first 100 days of the 2022 outbreak	Focus on estimation of epidemiological parameters
Gao et al., 2023	Monkeypox outbreaks in the context of the COVID-19 pandemic: Network and clustering analyses of global risks and modified SEIR prediction of epidemic trends	Dynamic model for forecasting mpox epidemic trends (Prediction)
Overton et al., 2023	Nowcasting the 2022 mpox outbreak in England	Model for nowcasting mpox epidemic trends
Pollock et al., 2023	Potential for Recurrent Mpox Outbreaks Among Gay, Bisexual, and Other Men Who Have Sex with Men - United States, 2023	Model for other purposes (association analysis)
Bleichrodt et al., 2023	Real-time forecasting the trajectory of monkeypox outbreaks at the national and global levels, July-October 2022	Model constructed for forecasting mpox epidemic trends
Marwan Al-Raeei, 2023	The study of human monkeypox disease in 2022 using the epidemic models: herd immunity and the basic reproduction number case	Focus on estimation of epidemiological parameters
Betti et al., 2023	A pair formation model with recovery: Application to mpox	Focus on estimation of epidemiological parameters
Fernandes et al., 2023	Behavioral aspects and the transmission of Monkeypox: A novel approach to determine the probability of transmission for sexually transmissible diseases	Model for other purposes (determination of the relation between sexual behaviours and mpox transmissibility)
Endo et al., 2022	Heavy-tailed sexual contact networks and monkeypox epidemiology in the global outbreak, 2022	Model for other purpose (exploration between the sexual partnership distribution and mpox outbreak size)
Ming et al., 2023	MSR72 Sexually Transmitted Diseases Outbreak Prediction and Transmission Dynamics Understanding Using Agent-Based Modeling: Take the Global Spread of Male-Dominated and Msm-Dominated Mpox Outbreak As an Example	Model for other purpose (prediction of mpox epidemic)

Kaftan et al., 2024	Performance analysis of mathematical methods used to forecast the 2022 New York City Mpox outbreak	Model for other purpose (forecasting of mpox epidemic)
Deng et al., 2024	The risk of mpox importation and subsequent outbreak potential in Chinese mainland: a retrospective statistical modelling study	Model for other purpose (assessments of mpox importation under border policies)
Jung et al., 2025	Dynamic Landscape of Mpox Importation Risks Driven by Heavy-Tailed Sexual Contact Networks Among Men Who Have Sex With Men in 2022	Model for other purpose (examining the international importation pattern and risks of mpox)
Zhang et al., 2025	Psychosocial correlates of free Mpox vaccination intention among men who have sex with men in China: model construction and validation	Ineligible model type (regression model)
Jin et al., 2025	Effectiveness of different border control strategies for reducing mpox importation risk: a modelling study	Model for other purpose (assessments of mpox importation under border policies)
Cain et al., 2025	Collective Efficacy: A Protective Factor for Sexual Minority Men During the U.S. Mpox Outbreak	Ineligible model type (regression model)

Supplemental Table D. Information on studies included in the review of mpox reproduction number, but not eligible for quantitative synthesis

Study	Reason for Exclusion from Quantitative Synthesis
Xiu et al., 2024[69]	Estimated R during 2022-2023, with cumulative cases of the estimation region not greater than 400 during the estimation period
Guzzetta et al., 2022[71]	Estimated R during 2022-2023, with cumulative cases of the estimation region not greater than 400 during the estimation period
Miura et al., 2024[72]	Estimated R during 2022-2023, with cumulative cases of the estimation region not greater than 400 during the estimation period
Althobaity et al., 2025[73]	Estimated R during 2022-2023, with cumulative cases of the estimation region not greater than 400 during the estimation period
Paredes et al., 2024[74]	Estimated R during 2022-2023, with period of R estimation not including the initial phase or the ascending part of the mpox epidemic
Zheng et al., 2023[75]	Estimated R during 2022-2023, with period of R estimation not including the initial phase or the ascending part of the mpox epidemic
Sun et al., 2023[70]	Estimated R during 2022-2023, with risk of bias (quality) assessment score not greater than 0.5
Guo et al., 2023[76]	Estimated R during 2022-2023, with risk of bias (quality) assessment score not greater than 0.5
Betti et al., 2023[77]	Estimated R during 2022-2023, with risk of bias (quality) assessment score not greater than 0.5
Liu et al., 2023[78]	Estimated R during 2022-2023, with risk of bias (quality) assessment score not greater than 0.5
Wei et al., 2022[79]	Estimated R during 2022-2023, with risk of bias (quality) assessment score not greater than 0.5
Marwan Al-Raeei, 2023[80]	Estimated R during 2022-2023, with risk of bias (quality) assessment score not greater than 0.5
Jia et al., 2024[61]	Estimated R during 2022-2023, with risk of bias (quality) assessment score not greater than 0.5
Gao et al., 2023[81]	Estimated R during 2022-2023, with risk of bias (quality) assessment score not greater than 0.5
Musafir et al., 2024[55]	Estimated R during 2022-2023, with risk of bias (quality) assessment score not greater than 0.5
Gushchin et al., 2022[21]	Estimated R during 2022-2023, with risk of bias (quality) assessment score not greater than 0.5
Marwan Al-Raeei, 2022[82]	Estimated R during 2022-2023, with risk of bias (quality) assessment score not greater than 0.5
Akhmetzhanov et al., 2024[83]	Estimated R during 2022-2023, with risk of bias (quality) assessment score not greater than 0.5

Supplemental Table E. Information on studies included in the review of the effects of mpox mitigation measures assessed by transmission dynamic model, but not eligible for quantitative synthesis

Study	Reason for Exclusion from Quantitative Synthesis
Bragazzi et al., 2023[45]	Theoretical simulation modellings, did not calibrate their models (with the mitigation measures implemented in the real world) to the observed epidemic curves
Cao et al., 2024[46]	Theoretical simulation modellings, did not calibrate their models (with the mitigation measures implemented in the real world) to the observed epidemic curves
Chaturvedi et al., 2024[47]	Theoretical simulation modellings, did not calibrate their models (with the mitigation measures implemented in the real world) to the observed epidemic curves
Chitwood et al., 2023[48]	Theoretical simulation modellings, did not calibrate their models (with the mitigation measures implemented in the real world) to the observed epidemic curves
Cordeiro et al., 2024[49]	Theoretical simulation modellings, did not calibrate their models (with the mitigation measures implemented in the real world) to the observed epidemic curves
Haridas K Das, 2024[50]	Theoretical simulation modellings, did not calibrate their models (with the mitigation measures implemented in the real world) to the observed epidemic curves
Dimitrov et al., 2023[40]	Theoretical simulation modellings, did not calibrate their models (with the mitigation measures implemented in the real world) to the observed epidemic curves
Gan et al., 2024[42]	Theoretical simulation modellings, did not calibrate their models (with the mitigation measures implemented in the real world) to the observed epidemic curves
Huang et al., 2024[44]	Theoretical simulation modellings, did not calibrate their models (with the mitigation measures implemented in the real world) to the observed epidemic curves
Idisi et al., 2023[51]	Theoretical simulation modellings, did not calibrate their models (with the mitigation measures implemented in the real world) to the observed epidemic curves
Jia et al., 2024[61]	Theoretical simulation modellings, did not calibrate their models (with the mitigation measures implemented in the real world) to the observed epidemic curves
Khan et al., 2024[52]	Theoretical simulation modellings, did not calibrate their models (with the mitigation measures implemented in the real world) to the observed epidemic curves
Knight et al., 2022[41]	Theoretical simulation modellings, did not calibrate their models (with the mitigation measures implemented in the real world) to the observed epidemic curves
Ko et al., 2023[53]	Theoretical simulation modellings, did not calibrate their models (with the mitigation measures implemented in the real world) to the observed epidemic curves
Leandry et al., 2023[54]	Theoretical simulation modellings, did not calibrate their models (with the mitigation measures implemented in the real world) to the observed epidemic curves
Murayama et al., 2024[66]	Theoretical simulation modellings, did not calibrate their models (with the mitigation measures implemented in the real world) to the observed epidemic curves
Musafir et al., 2024[55]	Theoretical simulation modellings, did not calibrate their models (with the mitigation measures implemented in the real world) to the observed epidemic curves
Omame et al., 2024[62]	Theoretical simulation modellings, did not calibrate their models (with the mitigation measures implemented in the real world) to the observed epidemic curves
Savinkina et al., 2023[43]	Theoretical simulation modellings, did not calibrate their models (with the mitigation measures implemented in the real world) to the observed epidemic curves
Savinkina et al., 2024[64]	Theoretical simulation modellings, did not calibrate their models (with the mitigation measures implemented in the real world) to the observed epidemic curves
Spicknall et al., 2022[57]	Theoretical simulation modellings, did not calibrate their models (with the mitigation measures implemented in the real world) to the observed epidemic curves

Van Dijck et al., 2023[56]	Theoretical simulation modellings, did not calibrate their models (with the mitigation measures implemented in the real world) to the observed epidemic curves
Yang et al., 2023[58]	Theoretical simulation modellings, did not calibrate their models (with the mitigation measures implemented in the real world) to the observed epidemic curves
Yuan et al., 2022[59]	Theoretical simulation modellings, did not calibrate their models (with the mitigation measures implemented in the real world) to the observed epidemic curves
Yuan et al., 2023[60]	Theoretical simulation modellings, did not calibrate their models (with the mitigation measures implemented in the real world) to the observed epidemic curves
Jin et al., 2025[65]	Theoretical simulation modellings, did not calibrate their models (with the mitigation measures implemented in the real world) to the observed epidemic curves
Omame et al., 2025[63]	Theoretical simulation modellings, did not calibrate their models (with the mitigation measures implemented in the real world) to the observed epidemic curves
Brand et al., 2023[29]	Empirical transmission dynamic modelling studies calibrating their models (with the mitigation measures implemented in the real world) to the observed epidemic curves, but did not estimate the proportion of cases prevented by the measures compared to counterfactual
Guzzetta et al., 2024[34]	Empirical transmission dynamic modelling studies calibrating their models (with the mitigation measures implemented in the real world) to the observed epidemic curves, but did not estimate the proportion of cases prevented by the measures compared to counterfactual
Zhang et al., 2025[32]	Empirical transmission dynamic modelling studies calibrating their models (with the mitigation measures implemented in the real world) to the observed epidemic curves, but did not estimate the proportion of cases prevented by the measures compared to counterfactual
Milwid et al., 2023[35]	Empirical transmission dynamic modelling studies calibrating their models (with the mitigation measures implemented in the real world) to the observed epidemic curves, but did not estimate the proportion of cases prevented by the measures compared to counterfactual
Xiridou et al., 2024[33]	Empirical transmission dynamic modelling studies calibrating their models (with the mitigation measures implemented in the real world) to the observed epidemic curves, but did not estimate the proportion of cases prevented by the measures compared to counterfactual
Xiong et al., 2024[37]	Empirical transmission dynamic modelling studies calibrating their model (with the mitigation measures implemented in the real world) to the observed epidemic curves, estimated the proportion of cases prevented by the measures compared to counterfactual, but did not report uncertainty interval estimates

Supplemental Table F. Data extraction form for papers of the mpox reproduction number estimates

Study	Method of R estimation	Period of R estimation	Country and its R estimates (95% CI or CrI) extracted
Fine et al., 1988[17]	Based on empirical data of contact rate and attack rate	1980-1984	Zaire* R_0: 0.815 (one-side 5% upper limit ≈1)
Blumberg et al., 2013[15]	Branching process model	1980-1984	Democratic Republic of Congo R_0: 0.30 (0.21, 0.42)
Kucharski et al., 2015[14]	Branching process model and next generation matrix	1970-1979	5 Central and West African countries: Zaire, Liberia, Nigeria, Ivory Coast, Sierra Leone<br>R_e: 0.08 (0.02, 0.22)
Grant et al., 2020[19]	Based on historical data of mpox R estimates and mathematical modelling	1980-1984, 2011-2012	Democratic Republic of Congo<br>1980-1984 R_0: 2.13 (1.46, 2.67)<br>2011-2012 R_e: 0.85 (0.51, 1.25)
Marwan Al-Raeei, 2022[82]	SIRD dynamic model and Jacobean matrix method	During 2022 mpox outbreak	Brazil R_0: 1.5589
Marwan Al-Raeei, 2023[80]	SIRD dynamic model	During 2022 mpox outbreak	USA R_0: 1.5508
Augsburger et al., 2022[84]	*EpiEstim* (version 2.2) package of R (renewal equation method)	From day 10 - day 55 into the 2022 mpox epidemics (max R_t occurred on day 15)	England maximum value of mean R_e: 4.8 (4.016, 5.584)
Betti et al., 2023[77]	pair formation SIR dynamic model and next generation matrix	May - December, 2022	Canada R_0: 2.81 (2.67, 2.95)
Borges et al., 2023[85]	SEIR dynamic model	April - October, 2022 (Portugal max Rt occurred on May 10, 2022)	Portugal maximum R_t: 2.70 (2.11, 3.30)
Bragazzi et al., 2023[45]	SEIQR compartmental model	May 19 - July 25, 2022	Canada R_0: 1.464
Brand et al., 2023[29]	SEPIR compartmental model and next generation matrix	May - September, 2022	UK R_0: 5.16 (2.96, 9.24)
Branda et al., 2023[86]	Renewal equation and Bayesian approach (with Poisson distributed transmission)	From the start of 2022 mpox global outbreak to the end of August, 2022	Germany median R_0: 3.62 (2.22, 6.38)
Du et al., 2022[87]	Renewal equation method	May 18 - July 22, 2022	USA median R_e: 1.95 (1.82, 2.10)
Gao et al., 2023[81]	Flight-SEIR dynamic model	January 31 - December 31, 2022 (early stage R_0: up to June 23)	Northern America early stage R_0: 7.841
Guo et al., 2023[76]	LoteKa-Euler equation	January - August 2022	Global R_t: 1.33
Guzzetta et al., 2022[71]	Renewal equation and Bayesian approach with Monte Carlo Markov Chain (MCMC) procedure	June 1 - June 7, 2022	Italy mean R_t: 2.43 (1.82, 3.26)
Kim et al., 2024[88]	Initial R_t: best-fit phenomenological dynamic growth models<br>Instantaneous R_t: *EpiEstim* package of R (renewal equation method)	Initial R_t: initial 30 days after report of the first local case in each country (April 28, 2023 – May 26, 2023 for China)<br>Instantaneous R_t:<br>start of mpox outbreak in each country - July 1, 2023	China initial R_t: 2.89 (1.44, 3.33)
Kwok et al., 2022[89]	Branching process model	May 18 - June 18, 2022	Spain R_0: 1.80 (1.70, 2.00)
Liao et al., 2023[90]	SIR dynamic model with Bayesian directed acyclic graphic (DAG) method	June 8 - July 7, 2022	UK† R_0 (using the deterministic SIR model): 2.432
Liu et al., 2023[78]	SEIR dynamic model	May 6 - June 30, 2022	Germany median R_0: 2.88 (2.64, 3.00)
Marín-Sánchez et al., 2023[91]	Mathematical modelling (Gompertz function or logistic regression), with graphical method or analytical approach for growth rate estimation	June 3, 2022 - April 30, 2023	South America R_0 (analytical + logistic): 1.94593
Miura et al., 2024[72]	Lotka-Euler equation	June 2022	Netherlands R_e: 1.3 - 1.6‡
Musa et al., 2022[20]	Renewal equation method	January 1, 2017 – August 27, 2022	Nigeria<br>2017 mean R_e: 1.8052 (0.8817, 4.8732)<br>2018 mean R_e: 1.0204 (0.7854, 1.5200)<br>2019 mean R_e: 1.0385 (0.7316, 1.7675)<br>2020 mean R_e: 1.1760 (0.6978, 1.6440)

			2021 mean R_e: 1.1693 (0.8257, 1.5825)<br>2022 mean R_e: 1.924 (1.455, 2.485)
Quispe et al., 2023[92]	An approach by Bonifazi Get al., based on the doubling time (or the growth rate), and generation time of mpox	During the first 100 days of the 2022 mpox outbreaks in Latin American and Caribbean countries (July 19 - August 22, 2022 for Colombia)	Colombia mean R_t: 3.832 (2.239, 6.419)[§]
Saldaña et al., 2023[93]	Renewal equation and filtering type Bayesian inference	June 25 - September 23, 2022 (early-phase R_t in Italy around June 29, 2022)	Italy early-phase R_t (proxy of R_0): 3.06 (2.48, 3.62)
Schrarstzhaupt et al., 2022[94]	*EpiEstim* package of R<br>(renewal equation method)	July 26 - August 22, 2022 (max R_t occurred on July 26, 2022)	The state of Goias´ plus Federal District (Brasília), Brazil<br>max value of mean R_t: 2.62
Sun et al., 2023[70]	2022 mpox outbreak: *EpiEstim* package of R (renewal equation method) and Bayesian approach	May 6 - July 22, 2022	Peru R_t: 4.24 (2.89, 6.71)
Wei et al., 2022[79]	SEIR dynamic model	May 10 - October 21, 2022	USA R_0: 3.167
Yang et al., 2022[95]	SEIR dynamic model and next generation matrix	May 6 - September 20, 2022	USA median R_0: 3.54 (3.04, 3.91)[¶]
Yang et al., 2023[58]	SEIR dynamic model	May 6 - July 1, 2022	Germany median R_e: 3.57 (3.36, 4.00)[¶]
Zhang et al., 2024[30]	*R0* package of R (exponential growth method, or maximum likelihood method), *EpiEstim* package of R (renewal equation method), modified renewal equation transmission model, SEEIIR compartmental models (homogeneous, or structured model)	April 17 - August 16, 2022 for compartmental model calibration, and August 13 - Nov 16, 2022 for validation	UK R_0: 1.94 (1.81, 2.19) under structured SEEIIR model
Zygmunt et al., 2023[96]	*EpiEstim* package of R<br>(renewal equation method)	May 1 - October, 2022 (max R_t occurred in the 7-day period from May 13, 2022)	Canada max R_t: 2.92 (1.04, 4.80)
Akhmetzhanov et al., 2024[83]	Formula by Wallinga and Lipsitch, based on the growth rate and generation time distribution of mpox	During 2022-2023 mpox outbreak (by August, 2023)	Colombia mean R_e during the exponential growth phase (July 6 - August 20, 2022): 2.31 (2.04, 2.60)
Bragazzi et al., 2024[97]	SEIQR compartmental model and next generation matrix	Early stages of the 2022 epidemic (June - September 2022) in each country (May 20 - June 28, 2022 for Germany)	Germany R_0: 3.677 (2.78, 4.61)[‖]
Cao et al., 2024[46]	modified SEIR compartmental model	May 10 - July 29, 2022	USA R_0: 2.73
Charniga et al., 2024[16]	*vimes* package of R for identification of case clusters and *branchr* package of R for branching process model	2013 - 2017	Democratic Republic of Congo R_t: 0.82 (0.79, 0.85)
Cordeiro et al., 2024[49]	SEIR compartmental model and next generation matrix	May, 2023 - October, 2023 (highest Rt occurred at the end of May, 2023)	Portugal highest R_t: 1.58 (1.45, 1.75)
Díaz-Brochero et al., 2024[98]	*EpiEstim* package of R<br>(renewal equation method)	May - November, 2022 (max R_t of Colombia occurred at the week of July 11, 2022)	Colombia max R_t: 3.15 (2.07, 4.44)
Gushchin et al., 2022[21]	*EpiEstimate* package of R (likely to be the *EpiEstim* package of R, renewal equation method)	May - July, 2022 (June, 2022 for the max R_0 of USA)	USA max R_0: 7.99
Guzzetta et al., 2024[34]	SEIRV network model	May 9 - September 5, 2022 (initial phase: May 9 - June 7, 2022)	Italy initial phase mean R_e: 1.97 (1.50, 2.51)
Hilton et al., 2024[18]	Beta-Poisson model for human transmission of mpox	1980 - 1984	Zaire[*] R_0: 0.3 (0.22, 0.4)
Jia et al., 2024[61]	SEPIR-SEIR two-layer network model and next generation matrix	May 18 - August 18, 2022	Canada R_0: 2.3475 (0.0749, 6.9084)
Lin et al., 2024[27]	SEIARV compartmental model	May 22 - December 22, 2022	USA R_0: 3.88
Marziano et al., 2024[22]	Renewal equation method and exponential growth rate model	September 29, 2023 - April 21, 2024	Kamituga Health Zone, South Kivu Province, Democratic Republic of Congo mean R_e: 1.18 (1.10, 1.26)

Mbelambela et al., 2025[25]	Equation based on the final epidemic prevalence	January, 2023 - November, 2023	Katako-Kombe Health Zone, Sankuru Province, Democratic Republic of the Congo R_0: 1.29
Musafir et al., 2024[55]	Fractional order mathematical model and next generation matrix	June 1 - October 5, 2022	USA R_0: 1.17
Paredes et al., 2024[74]	Phylodynamic model and exponential growth rate (based on effective population size) model	July, 2022 - January, 2023 (max value of mean R_t for North America occurred at the beginning of July, 2022)	North America maximum value of mean R_t: 1.49**
Xiu et al., 2024[69]	SEIR compartmental model and next generation matrix	May - July, 2022	Montréal, Canada R_0: 2.7 (2.4, 3.7)
Zhang et al., 2024[99]	Individual-instant heterogeneity model (branching process model)	May 31, 2023 - June 21, 2023	Beijing, China R_e: 1.68 (1.12, 2.41)
Zheng et al., 2023[75]	*EpiEstim* package of R (renewal equation method)	During the 2022 mpox outbreaks (by 28 October, 2022)	Argentina R_t around late-October: 1.03 (0.85, 1.23)
Zhang et al., 2025[32]	Time-varying homogeneous SEIVR dynamic model and next generation matrix	May, 2022 - September, 2023	USA R_0 in the initial development phase (May 28 to July 30, 2022): 3.1186
Althobaity et al., 2025[73]	Individual-instant heterogeneity model (extension of classic branching process model)	July 28 - August 17, 2022	Arab Gulf Countries Re: 0.95 (0.93, 1.35)

* Zaire was the former name of the Democratic Republic of the Congo (DRC) from 1971 to 1997.
† This study estimated a larger R value in the Democratic Republic of the Congo; however, they were considered as biased estimation due the quality of reporting data and the high proportion of animal-to-human transmission.
‡ under the range of average doubling time: 11.2 - 20.5 days.
§ lower and upper estimates using the generation time of 7.5 days and 17.3 days, respectively.
¶ Interquartile range (IQR)
|| 90% credible intervals (CI)
** Larger R were estimated for regions of Europe, but the numerical values of these estimates weren't reported.

Supplemental Table G. Dynamic modelling studies of mpox evaluating the impact of mitigation measures implemented in each country or region during the 2022–2023 mpox outbreak

Study	Model design	Model Setting and population	Modelling time frame	R_0 (transmission probability and contact rate if R_0 unavailable)	Mitigation measures	Infections or cases prevented compared to counterfactual (or other mitigation effects resulted from control measures)	Major factors attributable to the control and containment of the 2022-2023 mpox regional outbreaks
Brand et al., 2023[29]	Stochastic SEPIR compartmental epidemic model	• The UK N=67,200,000 (including 760,839 sexually active GBMSM divided into 10 sexual activity groups)	May 2022 to April 2023 (calibration: May 2022 to September 2022)	Overall R_0 on May 1: 5.16 (95% CI 2.96–9.24)<br>non-sexual pathway: 0.0398 (95% CI 0.00898–0.0716)<br>sexual contact pathway of MSM: 5.16 (95% CI 2.96–9.24)	• PrEP vaccination to the 9.4% most sexually active GBMSM<br>• Behavioural changes with 3 midpoints: decreased transmission rate per sexual contact between GBMSM, and decreased reproductive number for other transmission routes	• A 38.1% and a further 33.8% sexual risk reduction after the changing midpoint of June 11 and after the announcement of PHEIC, respectively, lead to the reduction of the reproductive number for the GBMSM sexual network and other routes (GBMSM R_0=3.02 [95% CI 1.94–4.31] before July 23, and dropped to 1.98 [95% CI 1.06–3.42] immediately after the announcement of PHEIC).<br>• Vaccination and behaviour changes prevented an uncontrolled outbreak that would have led to approximately a threefold increase in cumulative cases, reaching 11,601 by the end of March 2023.	Behaviour changes + infection induced immunity

Clay et al., 2024[28]	Stochastic SEPIRV dynamic network model	• The US, Washington DC<br>N=37,400 MSM, stratified into 6 sexual activity groups, with 5% in the highest sexual activity group	June 2, 2022 to June 2, 2023 (calibration: June 2022 to January 10, 2023)	• Transmission probability per sexual contact: 0.51<br>• Daily probability of one-time sexual contact among individuals in activity group 6: 0.3<br>• Per time-step probability of sexual contact with main partner (casual partner): 0.22 (0.14)	• PrEP Vaccination<br>• Behavioural adaptation: time-varying reduction in daily one-time sexual contact rate (maximum reduction: 39%)	% of cases prevented at initial outbreak phase (by mid-August, 2022):<br>• Vaccination: 3.8% (IQR -8.3% to 19%)<br>• Behavioural adaptation: 13% (IQR -3.5% to 30%)<br>• Both measures: 15% (IQR -2.0% to 30%)<br>% of cases prevented in a year (by June 2023):<br>• Vaccination: 79% (IQR 64% to 88%)<br>• Behavioural adaptation: 25% (IQR 10% to 42%)<br>• Both: 84% (IQR 67% to 91%)<br>• Vaccination was necessary to end the outbreak within a year, and earlier vaccination would be more effective in reducing cases throughout the outbreak than delayed administration for more vaccines available and potentially higher coverage.	Vaccination + behaviour changes

Clay et al., 2025[38]	Stochastic SEPIRV dynamic network model	• The US, New York City (NYC).<br>N=167,000 GBMSM, stratified into 6 sexual activity groups, with 1% in the highest sexual activity group	May 14, 2022 to May 14, 2023 (calibration: May 14, 2022 to January 18, 2023)	• Transmission probability per exposure: 0.67<br>• Daily probability of one-time sexual contact among individuals in activity group 6: 0.37<br>• Per time-step probability of sexual contact with main partner (casual partner): 0.22 (0.14)	• Targeted PrEP vaccination (first-dose priority strategy [baseline], intermediate strategy [counterfactual], and second-dose priority strategy [counterfactual])<br>• Behavioural adaptation: time-varying reduction in daily one-time or casual sexual contact rate (maximum reduction: 49%)	% of cases prevented in a year compared to the counterfactual scenario with no vaccination (by May 14, 2023):<br>• First dose priority vaccination: 67% (IQR 47% to 78%)<br>• The first-dose priority strategy implemented in NYC would have prevented 0.61% (IQR -11% to 9.8%) and 17% (IQR 2.9% to 38%) more cases by one year compared to the intermediate strategy and the second-dose priority strategy, respectively.	Did not specify
Guzzetta et al., 2024[34]	Stochastic SEIRV individual-based network model	• Italy<br>N=5,300,000 (Italian MSM population and their household members, where the 1,670,000 sexually active MSM were divided into 5 risk groups, 2.5% HR and 20.5% moderate risk)	May 9, 2022, to February 28, 2023 (calibration: May 9, 2022, to September 5, 2022)	• Overall R_0: 1.97 (95% PI 1.50–2.51)<br>• R_0 specific to sexual contact network: 1.52 (95% PI 0.97–2.23)<br>• R_0 in clubs: 2.75 (95% PI 0.54–6.17)	• PrEP vaccination<br>• Reduction of transmission rate in the sexual network (61%) and in clubs (87%) since June 8, 2022<br>• Full isolation of the diagnosed cases and 20% of their sexual contacts traced	• In August, the reproduction number decreased to 0.87 (95% PI 0.74–0.97) (overall), 0.63 (95% PI 0.49–0.78) (within sexual contact network), and 0.20 (95% PI 0.00–0.87) (in clubs).<br>• The baseline scenario (vaccination + reduced transmissibility + 20% contact tracing) end up with half the cumulative infections by February 28, 2023 compared to the scenario without contact tracing.<br>• Increasing the coverage of contact tracing to 40% and 80% reduced an average of 42% and 62% of cumulative	Behaviour changes + contact tracing and isolation

						infections compared to baseline scenario, and increased the probability of stochastic extinction.	
Liang et al., 2024[31]	Microsimulation model (considering status of susceptible, asymptomatic, symptomatic, recovered, and vaccinated)	• The US, Los Angeles County<br>N=262,912 MSM in Los Angeles County	July 3, 2022 to April 9, 2023 (calibration: July 3, 2022 to September 18, 2022)	Initial force of infection (transmission rate) : 2.2	• Vaccination (PrEP + PEP)<br>• Reductions in sexual partnership: force of infection (transmission rate) decreased from 2.2 to 0.7 after August, 2022	• Compared to status quo, cumulative cases would increase by 28% if there were no vaccination campaign, while delaying the reduction in sexual partnerships by 3 weeks would lead to a 95% increase in cumulative cases.<br>• About 42 cases were averted for every 10000 vaccine doses.<br>Increase or decrease in cumulative cases under different scenarios compared to status quo:<br>(1) halving the vaccination rate: 8% increase in cases<br>(2) doubling the vaccination rate: 13% decrease in cases<br>(3) vaccination campaign began 2 weeks earlier: 11% decrease in cases<br>(4) vaccination campaign delayed for 2, 4, or 8 weeks: 10%, 13%, or 20% increase in cases<br>(5) vaccine targeting people with HIV (PWH): 7% decrease in cases<br>(6) vaccine targeting Black MSM, Hispanic	Vaccination + behaviour changes

						MSM, or White MSM: 3%, 13%, or 9% increase in cases<br>(7) 3-week delay in behavioural changes: 95% increase in cases<br>(8) 3-week earlier in behavioural changes: 60% decrease in cases<br>(9) half rebound in sexual partnerships in October, November, or December 2022: 14%, 4%, or 0.09% increase in cases<br>(10) full rebound in sexual partnerships in October, November, or December 2022: 453%, 108%, or 23% increase in cases	
Lin et al., 2024[27]	Deterministic compartmental SEIARV model	• The US<br>N=2,873,284<br>4% HR MSM	May 22, 2022 to December 22, 2022 (calibration: May 22, 2022 to December 22, 2022)	LR-R_0: 0.39<br>HR-R_0: 3.88	• PrEP vaccination<br>• 25% decreased in high-risk group sexual risk	% of cases prevented by December 22, 2022:<br>• Vaccination: 21.2% (10.2%–24.1%)<br>• High-risk group sexual risk reduction: 15.4% (14.3%–20.6%)<br>• Combination of both measures: 64.0% (43.8%–69.0%)<br>• Both vaccination campaign and reductions in sexual risk among MSM contributed to the control of mpox epidemic in the US, with strong synergistic effects observed.	Vaccination + behaviour changes

Milwid et al., 2023[35]	Stochastic SEIR compartmental model and SEIR agent-based model (ABM)	• Canada<br>1. Compartmental model (Canada national model and provincial model of Ontario, Quebec, and British Columbia):<br>N (national model) = Population in Canada (1.5% GBMSM, 50% of them in the effective population)<br><br>2. ABM:<br>N=10000 GBMSM aged 15 years and older, living in Montreal, Quebec	April 2022 to December 2023 (calibration: April 2022 to December 2022 for the compartmental model, and May 2022 to August 14, 2022 for ABM)	Compartmental model:<br>Initial transmission rate: 0.0988/day (Canada national model)<br>ABM:<br>Transmission probability per act: 0.6	Compartmental model:<br>• Vaccination<br>• Reduction of transmission rate to 0.0484 at July 27, 2022 (status quo scenario), or reduced transmission rate such that R_t≈1 (partial control scenario)<br>ABM:<br>• Vaccination	National level, compartmental model:<br>• In the status quo scenario: cases peaked in mid–July (30 cases/day) and approached zero between September and December 2022. However, lowering the vaccine effectiveness and the effective proportion of GBMSM (vaccinated proportion) showed minimal influence on the epidemic projections.<br>• In the uncontrolled outbreak scenario (no transmission rate reduction): cases peaked in late-August (4200 cases/day).<br>• In the partial control scenario: cases peaked with continued plateaued since July with 30 cases/day.<br>ABM:<br>• Widening vaccination to men with at least one annual sexual partner: increased infections by 47% (credible interval −27% to 182%) and delayed the epidemic peak.<br>• The epidemic trajectories remained largely unchanged when vaccine uptake was linearly reduced to 0% from August 2022.	Behaviour changes

						• Vaccination reduced the size and duration of the outbreak in the scenario without behaviour adaptations.	
Shamier et al., 2024[39]	Stochastic compartmental SEIR model	• The Netherlands<br>N=45,000-60,000 MSM	May 2022<br>to January 2023<br>(calibration: May 2022<br>to January 2023)<br>and<br>a future outbreak simulation of 250 days	Transmissibility (transmission coefficient): 0.5-1	In the current 2022-2023 outbreak:<br>• High risk group vaccination<br>• 50% reduction in sexual partners after May 23, 2022<br>• reduced time to-diagnosis and earlier isolation<br>Evaluation of future outbreak scenarios under combinations with or without:<br>A. Seroprevalence 35–55% (partially vaccinated in 2022 + prior infection-induced immunity)<br>B. 50% reduction in sexual partners<br>C. Reduced time to-diagnosis and earlier isolation	• Effect of vaccination: 7% cases prevented in the current 2022-2023 outbreak (1325 cumulative cases [IQR 1262–1419]) compared to no vaccination scenario (1427 cumulative cases [IQR 1321–1565])<br>Epidemic size of a future outbreak, and the percentage reduced compared with the 2022–2023 outbreak:<br>• Only immunity from prior infections or childhood smallpox vaccinations: 1,105 (IQR 1000–1,206), 16.6%<br>• A+B: 179 (IQR 108–265), 86.4%<br>• A: 344 (IQR 167–526), 74.0%<br>• A+C: 2 (IQR 0–6), no outbreak<br>• A+B+C: 2 (IQR 0–6), no outbreak<br>• A+B with demographic turnover rate 1-5%: mean outbreak size in 10 years range from 254 (turnover rate 1%) – 725 (turnover rate 5%)<br>• Seroprevalence for VACV-specific anti bodies: 45.4% and 47.1% among MSM in Amsterdam and Rotterdam in	Did not specify (but claimed that vaccination campaign offered negligible contributions)

						September 2022<br>• HR-vaccination has only marginal effects and should be independent of the containment of 2022-2023 outbreak in the Netherlands, but reduce the likelihood and size of future mpox outbreaks.	
Xiong et al., 2024[37]	Deterministic compartmental SEPIRV model	• The US, New York City<br>N=8,930,005 citizens in New York City<br>High-risk group: 127,030 gay and bisexual men, divided into 5 age groups	May 19, 2022 to November 3, 2022<br>(calibration: May 19, 2022 to November 3, 2022)	Mean R_e on June 28: about 1.3	• Targeted vaccination (first dose)<br>• Protection of previous smallpox vaccination (for 90% of population over 44 years, with a 92-year of protection half-life)<br>• Time-dependent contact rate in high-risk group	• Compared to the base scenario, the scenario in the absence of current mpox vaccination has seen the cumulative new mpox infection increase by 385%, and the epidemic duration increase by about 350%.<br>• Under the scenario with completion of vaccination (in accordance with the current age-based vaccine allocation in New York City) before the mpox outbreak, 94.2% of cases were prevented compared to baseline scenario. This result also indicated that New York City will not experience a mpox epidemic as severe as that of 2022 in the near future, even if festival-like events (that lead to increased sexual contact rates) were to take place.<br>• By sensitivity analysis on effective	Did not specify (but emphasised the importance of vaccination)

						reproduction number and the cumulative number of new cases in relation to vaccination rate: vaccine administration in 34-45 years high-risk group, and vaccination before June 23, 2022 (before the large-scale outbreak), was the most effective.	
Xiridou et al., 2024[33]	Deterministic compartmental SEIYHR model	• The Netherlands<br>N=250,000 MSM divided into 4 sexual activity groups.<br>17.7% fairly high, 0.7% very high risk.	April 27, 2022 to December 31, 2022<br>(calibration: April 2022 to<br>July 2022)	• Transmission probability per sexual contact: 0.48 (95% CrI 0.39–0.81) in the model with behavioural adaptations in July, and 0.48 (95% CrI 0.39–0.67) in the model with behavioural adaptations in June and July	• Vaccination rate (primary PEP) since June 8, 2022<br>• Behavioural adaptations in July (or in June and July)<br>A. Reduction of infectious period not refraining from sex to 2.6 days (to 5.6 days in June and 2.4 days in July)<br>B. 13-24% (16-18% (June), 13-22% (July)) reduction in the number of casual partners	• The model scenario without behaviour adaptations peaked around July 8, 2022, similar to the observed data, but exhibited a less steep decline trajectory.<br>• In the absence of behavioural adaptations (in July 2022), the mpox outbreak fade-out was delayed from November to December 2022.<br>• Introduction of unvaccinated, infectious high-risk MSM into the population would lead to a slight increase in mpox cases.	Infection induced immunity and behaviour changes
Xiu et al., 2025[36]	Deterministic compartmental SEIRVJOC model	• Canada (Montréal, Toronto, and Vancouver)<br>N=population of GBM in the above three cities (54000, 78000, 26100,	May to November, 2022<br>(calibration: May to October, 2022)	• β (risk of transmission per effective contact): 86%<br>• Mixing contact matrix	• Targeted vaccination (first dose)<br>• Reduction in the number of sexual partners: 20% and 33% among those having <=7 and >7 partners, respectively, based	Cumulative averted fraction of new infections:<br>• Montréal<br>• Vaccination: 21% (95% CrI 16%–33%)<br>Reduction in sexual partners: 15%	Vaccination + behaviour changes + contact tracing/isolation

		respectively), stratified into 5 age groups and 10 sexual activity groups, and HIV status.			on online questionnaires of 1445 GBM from the *Engage Cohort Study* (compartmental model-calibrated estimates [6% and 6%, respectively] were employed in the main model as the empirical survey-based behaviour change estimates did not fit observed data well)<br>• 7.0-7.9% contact tracing (77-86% of case reported * 20% of contacts of the reported cases being traced * 46% of contacts traced before becoming infectious)	(95% CrI 3%–34%)<br>• Contact tracing: 14% (95% CrI 12%–21%)<br>• All 3: 48% (95% CrI 35%–66%)<br>• Toronto<br>• Vaccination: 22% (95% CrI 16%–41%)<br>• Reduction in sexual partners: 11% (95% CrI 2%–27%)<br>• Contact tracing: 14% (95% CrI 12%–22%),<br>• All 3: 46% (95% CrI 34%–67%)<br>• Vancouver<br>• Vaccination: 39% (95% CrI 35%–48%)<br>• Reduction in sexual partners: 10% (95% CrI 2%–22%)<br>• Contact tracing: 14% (95% CrI 12%–16%)<br>• All 3: 58% (95% CrI 52%–69%)<br>• Standardising vaccination initiation date and first dose coverage across the three Canadian cities yielded identical estimates of cumulative averted fraction of new infections from vaccination (41%, 38%, and 39%, respectively).	

Zhang et al., 2024[30]	Deterministic compartmental SEEIIR model	• The UK<br>N=769,000 GBMSM<br>11.9% HR	April 17, 2022 To January 31, 2023 (calibration: April 17, 2022 to August 12, 2022)	Overall R_0: 1.51 (95% CrI 1.41–1.71)<br>LR: 0.67 (95% CrI 0.62–0.75)<br>HR: 1.94 (95% CrI 1.81–2.19)	• PrEP Vaccination<br>• 44.5% decrease in sexual contact rate (changing mid-point: June 6, 2022)<br>• 20% decrease in infectious period (changing mid-point: June 6, 2022)	% of Infections prevented by November 16, 2022:<br>• Vaccination: 9.8% (95% CrI 0.4–29.9)<br>• Decrease in sexual contact rate: 81.7% (95% CrI 49.3–96.8)<br>• Decrease in infectious period: 47.4% (95% CrI 8.9–85.4)<br>• Combination of all three measures: 98.1% (95% CrI 97.2–98.5)<br>• Vaccination could avert 2.5 – 4.5 times more infections (44.5% [95% CrI 24.9–77.5]) if initiated on the second week of the outbreak.	Behaviour changes + diagnosis and isolation
Zhang et al., 2025[32]	Two-layer network model (including homogeneous and heterogeneous) + time-varying homogeneous SEIVR model	• The US<br>N= $9*10^6$ (both MSM and non-MSM population in the US) assuming only a proportion of the general population was at risk of mpox, and the MSM accounted for 50% of the model population	May 2022 to September 2023 (calibration: May 2022 to July 2023)	R_0 in the first phase (May 28 to July 30, 2022): 3.1186	• Vaccination (two doses, proportionally administered to the MSM and non-MSM)<br>• Reduction in sexual transmission rate among MSM (β_s^{m1}) and non-MSM (β_s^{o1}), and non-sexual transmission rate among all population (β_n^1) over three phases: β_s^{m1} from 0.8047, to 0.1110, and to 0.3615. β_s^{o1}=β_n^1 from 0.3503, to 0.0550, and to 0.2524.	• The epidemic declined to lower risk after behavioural changes of the population, with the R_0 dropped from 3.1186 during the initial development phase (by July 30) to 0.4492 in the slow growth phase (August 6, 2022 to May 6, 2023) and rebound to 1.7119 after May 13, 2023.<br>• Vaccination campaign in the US only caused a non-significant decrease in R_e compared to the no vaccination scenario, but could be more effective in	Behaviour changes

						reducing peak size of the outbreak when targeting only the MSM population.	

All 13 studies listed in the table calibrated the mpox transmission models to their respective epidemic curves. N denotes the model population.

Abbreviations: ABM, agent-based model; GBMSM or GBM, gay, bisexual, and other men who have sex with men; MSM, men who have sex with men. R_0, basic reproduction number; R_e, effective reproduction number; R_t, time-dependent reproduction number; PrEP, pre-exposure prophylaxis; PEP, post-exposure prophylaxis; PHEIC, public health emergency of international concern; IQR, interquartile range; PI, prediction interval; CI, confidence interval; CrI, credible interval; LR, low-risk; HR, high-risk.

Supplemental Table H. Risk of bias **(**quality) assessment score of the included studies, by study type

Empirical modellings refer to studies that calibrated their mpox transmission models (with mitigation measures implemented in the real-world status quo scenario) to their respective epidemic curves. Theoretical simulation modellings refer to studies that did not calibrate their model to real-world epidemics or models that performed calibration but assessed the potential effects of public health interventions across exploratory scales. The descriptions of each assessment item (Q1–Q7) are provided in Supplemental Text C. Each item was equally weighted. “Yes” responses were counted as fulfilled items and “No” as unfulfilled items. Items marked as “NA” were considered not applicable and excluded from the denominator. The overall score represents the proportion of applicable items fulfilled, ranging from 0 to 1, with higher values indicating better quality.

Study	Q1	Q2	Q3	Q4	Q5	Q6	Q7	Proportion score	Year	Study type
Blumberg et al., 2013[15]	Yes	Yes	No	No	Yes	Yes	No	0.571	2013	R before 2022
Charniga et al., 2024[16]	Yes	No	Yes	No	Yes	Yes	Yes	0.714	2024	R before 2022
Fine et al., 1988[17]	Yes	Yes	Yes	Yes	Yes	No	No	0.714	1988	R before 2022
Grant et al., 2020[19]	No	No	No	NA	No	Yes	No	0.143	2020	R before 2022
Hilton et al., 2024[18]	Yes	Yes	Yes	No	Yes	Yes	No	0.714	2024	R before 2022
Kucharski et al., 2015[14]	Yes	Yes	Yes	No	Yes	No	NA	0.667	2015	R before 2022
Akhmetzhanov et al., 2024[83]	Yes	No	No	No	Yes	Yes	No	0.429	2024	R after 2022
Marwan Al-Raeei, 2022[82]	Yes	Yes	No	No	No	No	NA	0.333	2022	R after 2022
Marwan Al-Raeei, 2023[80]	Yes	Yes	No	No	No	No	NA	0.333	2023	R after 2022
Althobaity et al., 2025[73]	Yes	Yes	Yes	Yes	No	Yes	No	0.714	2025	R after 2022
Augsburger et al., 2022[84]	Yes	Yes	Yes	Yes	Yes	Yes	No	0.857	2022	R after 2022
Betti et al., 2023[77]	Yes	Yes	No	No	Yes	No	NA	0.5	2023	R after 2022
Borges et al., 2023[85]	Yes	Yes	Yes	Yes	Yes	Yes	No	0.857	2023	R after 2022
Bragazzi et al., 2023[45]	Yes	Yes	Yes	Yes	Yes	Yes	No	0.857	2023	R after 2022
Bragazzi et al., 2024[97]	Yes	Yes	Yes	No	Yes	Yes	No	0.714	2024	R after 2022
Brand et al., 2023[29]	Yes	Yes	Yes	Yes	Yes	Yes	Yes	1	2023	R after 2022
Branda et al., 2023[86]	Yes	Yes	Yes	Yes	Yes	Yes	Yes	1	2023	R after 2022
Cao et al., 2024[46]	Yes	Yes	No	No	Yes	Yes	No	0.571	2024	R after 2022
Cordeiro et al., 2024[49]	Yes	Yes	Yes	No	No	Yes	No	0.571	2024	R after 2022
Díaz-Brochero et al., 2024[98]	Yes	Yes	Yes	No	Yes	Yes	No	0.714	2024	R after 2022
Du et al., 2022[87]	Yes	Yes	No	Yes	Yes	No	NA	0.667	2022	R after 2022
Gao et al., 2023[81]	Yes	No	Yes	No	No	Yes	No	0.429	2023	R after 2022

Guo et al., 2023[76]	Yes	No	Yes	No	Yes	NA	No	0.5	2023	R after 2022
Gushchin et al., 2022[21]	NA	No	No	No	Yes	Yes	No	0.333	2022	R after 2022
Guzzetta et al., 2022[71]	Yes	Yes	Yes	Yes	Yes	Yes	Yes	1	2022	R after 2022
Guzzetta et al., 2024[34]	Yes	Yes	Yes	No	Yes	Yes	No	0.714	2024	R after 2022
Jia et al., 2024[61]	No	Yes	No	No	Yes	No	NA	0.333	2024	R after 2022
Kim et al., 2024[88]	Yes	Yes	Yes	Yes	Yes	Yes	Yes	1	2024	R after 2022
Kwok et al., 2022[89]	Yes	Yes	Yes	Yes	Yes	Yes	Yes	1	2022	R after 2022
Liao et al., 2023[90]	Yes	Yes	Yes	No	Yes	Yes	No	0.714	2023	R after 2022
Lin et al., 2024[27]	Yes	Yes	Yes	No	Yes	Yes	No	0.714	2024	R after 2022
Liu et al., 2023[78]	Yes	No	Yes	No	Yes	No	NA	0.5	2023	R after 2022
Marín-Sánchez et al., 2023[91]	Yes	No	Yes	Yes	Yes	Yes	No	0.714	2023	R after 2022
Marziano et al., 2024[22]	Yes	Yes	Yes	No	No	Yes	No	0.571	2024	R after 2022
Mbelambela et al., 2024[25]	Yes	Yes	Yes	Yes	No	Yes	No	0.714	2024	R after 2022
Miura et al., 2024[72]	Yes	Yes	No	No	Yes	Yes	Yes	0.714	2024	R after 2022
Musa et al., 2022[20]	Yes	Yes	Yes	No	Yes	No	No	0.571	2022	R after 2022
Musafir et al., 2024[55]	Yes	No	Yes	No	No	No	No	0.286	2024	R after 2022
Paredes et al., 2024[74]	Yes	No	Yes	No	Yes	Yes	No	0.571	2024	R after 2022
Quispe et al., 2023[92]	Yes	Yes	Yes	No	Yes	No	No	0.571	2023	R after 2022
Saldaña et al., 2023[93]	Yes	Yes	Yes	Yes	Yes	Yes	No	0.857	2023	R after 2022
Schrarstzhaupt et al., 2022[94]	Yes	Yes	Yes	Yes	Yes	Yes	Yes	1	2022	R after 2022
Sun et al., 2023[70]	Yes	No	Yes	No	Yes	No	NA	0.5	2023	R after 2022
Wei et al., 2022[79]	No	Yes	No	Yes	No	NA	NA	0.4	2022	R after 2022
Xiu et al., 2024[69]	Yes	Yes	Yes	Yes	Yes	Yes	No	0.857	2024	R after 2022
Yang et al., 2022[95]	Yes	Yes	Yes	Yes	Yes	No	NA	0.833	2022	R after 2022
Yang et al., 2023[58]	Yes	Yes	Yes	Yes	Yes	Yes	No	0.857	2023	R after 2022
Zhang et al., 2024[30]	Yes	Yes	Yes	Yes	Yes	Yes	No	0.857	2024	R after 2022
Zhang et al., 2024[99]	Yes	Yes	Yes	No	Yes	Yes	No	0.714	2024	R after 2022
Zhang et al., 2025[32]	Yes	Yes	Yes	No	Yes	Yes	No	0.714	2025	R after 2022
Zheng et al., 2023[75]	Yes	Yes	Yes	No	No	No	NA	0.5	2023	R after 2022
Zygmunt et al., 2024[96]	Yes	Yes	Yes	Yes	Yes	Yes	No	0.857	2024	R after 2022

Bragazzi et al., 2023[45]	Yes	Yes	Yes	Yes	Yes	Yes	No	0.857	2023	theoretical simulation modelling
Cao et al., 2024[46]	Yes	Yes	Yes	No	Yes	Yes	No	0.714	2024	theoretical simulation modelling
Chaturvedi et al., 2024[47]	Yes	Yes	Yes	No	Yes	Yes	No	0.714	2024	theoretical simulation modelling
Chitwood et al., 2023[48]	Yes	No	No	No	Yes	Yes	No	0.429	2023	theoretical simulation modelling
Cordeiro et al., 2024[49]	Yes	Yes	No	No	Yes	Yes	No	0.571	2024	theoretical simulation modelling
Haridas K Das, 2024[50]	Yes	Yes	Yes	No	Yes	Yes	No	0.714	2024	theoretical simulation modelling
Dimitrov et al., 2023[40]	Yes	Yes	Yes	No	Yes	Yes	No	0.714	2023	theoretical simulation modelling
Gan et al., 2024[42]	No	Yes	No	No	No	Yes	No	0.286	2024	theoretical simulation modelling
Huang et al., 2024[44]	Yes	Yes	Yes	No	Yes	Yes	No	0.714	2024	theoretical simulation modelling
Idisi et al., 2023[51]	Yes	Yes	No	No	No	Yes	No	0.429	2023	theoretical simulation modelling
Jia et al., 2024[61]	No	Yes	No	No	Yes	No	NA	0.333	2024	theoretical simulation modelling
Jin et al., 2025[65]	Yes	Yes	Yes	No	Yes	Yes	No	0.714	2025	theoretical simulation modelling
Khan et al., 2024[52]	Yes	Yes	No	No	Yes	Yes	No	0.571	2024	theoretical simulation modelling
Knight et al., 2022[41]	Yes	Yes	No	No	Yes	Yes	No	0.571	2022	theoretical simulation modelling
Ko et al., 2023[53]	Yes	No	No	No	No	Yes	No	0.286	2023	theoretical simulation modelling
Leandry et al., 2023[54]	No	Yes	No	No	Yes	No	No	0.286	2023	theoretical simulation modelling
Murayama et al., 2024[66]	Yes	No	Yes	No	Yes	No	No	0.429	2024	theoretical simulation modelling
Musafir et al., 2024[55]	Yes	No	Yes	No	No	No	NA	0.333	2024	theoretical simulation modelling
Omame et al., 2024[62]	No	Yes	Yes	No	Yes	Yes	No	0.571	2024	theoretical simulation modelling
Omame et al., 2025[63]	Yes	Yes	Yes	No	No	Yes	No	0.571	2025	theoretical simulation modelling
Savinkina et al., 2023[43]	Yes	Yes	Yes	No	Yes	Yes	No	0.714	2023	theoretical simulation modelling
Savinkina et al., 2024[64]	Yes	Yes	No	No	Yes	Yes	No	0.571	2024	theoretical simulation modelling
Spicknall et al., 2022[57]	Yes	Yes	No	No	Yes	Yes	No	0.571	2022	theoretical simulation modelling
Van Dijck et al., 2023[56]	No	Yes	Yes	No	Yes	Yes	No	0.571	2023	theoretical simulation modelling
Yang et al., 2023[58]	No	Yes	Yes	No	Yes	Yes	No	0.571	2023	theoretical simulation modelling
Yuan et al., 2022[59]	Yes	Yes	No	No	Yes	No	No	0.429	2022	theoretical simulation modelling
Yuan et al., 2023[60]	Yes	Yes	No	No	Yes	No	NA	0.5	2023	theoretical simulation modelling
Brand et al., 2023[29]	Yes	Yes	Yes	No	Yes	Yes	Yes	0.857	2023	empirical modelling
Clay et al., 2024[28]	Yes	No	Yes	Yes	Yes	Yes	No	0.714	2024	empirical modelling
Clay et al., 2025[38]	Yes	Yes	No	No	No	Yes	No	0.429	2025	empirical modelling

Guzzetta et al., 2024[34]	Yes	Yes	Yes	No	Yes	Yes	No	0.714	2024	empirical modelling
Liang et al., 2024[31]	Yes	Yes	Yes	No	Yes	Yes	No	0.714	2024	empirical modelling
Lin et al., 2024[27]	Yes	Yes	Yes	Yes	Yes	Yes	No	0.857	2024	empirical modelling
Milwid et al., 2023[35]	No	Yes	Yes	No	Yes	No	No	0.429	2023	empirical modelling
Shamier et al., 2024[39]	Yes	Yes	No	No	Yes	Yes	No	0.571	2024	empirical modelling
Xiong et al., 2024[37]	Yes	Yes	Yes	Yes	Yes	No	No	0.714	2024	empirical modelling
Xiridou et al., 2023[33]	No	Yes	Yes	Yes	Yes	No	No	0.571	2023	empirical modelling
Xiu et al., 2025[36]	Yes	Yes	Yes	Yes	Yes	Yes	No	0.857	2025	empirical modelling
Zhang et al., 2024[30]	Yes	Yes	Yes	Yes	Yes	Yes	No	0.857	2024	empirical modelling
Zhang et al., 2025[32]	Yes	Yes	No	No	No	Yes	No	0.429	2025	empirical modelling

Supplemental Table I. Certainty of evidence for effectiveness of vaccination campaigns without or with behaviour change

Summary of findings:

Mpox Vaccination Campaigns without or with Behaviour Change Versus the Counterfactual without Intervention Measures

Patient or population: Population at risk of mpox
Setting:
Intervention: Vaccination campaigns without or with behaviour change
Comparison: Counterfactual scenario without intervention measures

Outcomes	№ of model population (studies)	Certainty of the evidence (GRADE)	Relative effect (95% CI)	Comments
Numbers of mpox cases (Vaccination campaigns alone)	4320196 (7 modelling studies)	⨁⨁⨁◯ Moderate	**Prevention percentage*** **0.24** (0.15 to 0.33)	Although dynamic model-based, all estimates were derived from explicitly pre-specified counterfactuals and models calibrated to real-world data, reducing bias concerns. The high I^2 of the pooled estimate was largely explained by meta-regression with key covariates ([1] first-dose vaccine coverage, and [2] time delay (in weeks) between first reported case and vaccination rollout, R^2=91.8%), so inconsistency was not considered serious. Vaccination campaigns administered to the at-risk population reduced mpox cases compared to the counterfactual scenario without measures.
Numbers of mpox cases (Vaccination campaigns with behaviour change)	3837784 (4 modelling studies)	⨁⨁⨁⨁ High	**Prevention percentage** **0.55** (0.37 to 0.73)	Although dynamic model-based, all estimates were derived from explicitly pre-specified counterfactuals and models calibrated to real-world data, reducing bias concerns. The high I^2 of the pooled estimate was almost entirely explained by meta-regression with key covariates ([1] extent of behaviour adaptation, [2] first-dose vaccine coverage, and [3] time delay (in weeks) between first reported case and vaccination rollout, R^2=99.8%), so inconsistency was not considered serious. Vaccination campaigns administered to the at-risk population with behaviour change toward safer practice largely reduced mpox cases compared to the counterfactual scenario without measures.

* Percentage of cases prevented compared to counterfactual (prevention percentage) is presented in decimal form (e.g., 0.24 = 24%).

CI: confidence interval

GRADE Working Group grades of evidence
High certainty: we are very confident that the true effect lies close to that of the estimate of the effect.
Moderate certainty: we are moderately confident in the effect estimate: the true effect is likely to be close to the estimate of the effect, but there is a possibility that it is substantially different.
Low certainty: our confidence in the effect estimate is limited: the true effect may be substantially different from the estimate of the effect.
Very low certainty: we have very little confidence in the effect estimate: the true effect is likely to be substantially different from the estimate of effect.

References

1. DC Health. Health Notice for District of Columbia Health Care Providers Mpox Virus Infection in the United States and Other Non-endemic Countries – 2022 (UPDATED) Potential Risk for New Mpox Cases, 22 June, 2023. Available from: https://dchealth.dc.gov/sites/default/files/dc/sites/doh/publication/attachments/Health%20Notice_Mpox_2023.6.22_Approved.pdf [Accessed 3 Mar 2025].
2. County of Los Angeles public health. Mpox Dashboard, 27 February, 2025. Available: https://app.powerbigov.us/view?r=eyJrIjoiNGI5MmEyMmQtYzVjMS00OTgwLWFhMDgtM2YyMTkxMjJlNmFhIiwidCI6IjA3NTk3MjQ4LWVhMzgtNDUxYi04YWJlLWE2MzhlZGRiYWM4MSJ9 [Accessed 3 Mar 2025].
3. Dutch National Institute for Public Health and the Environment (RIVM). Mpox vaccinations in 2022, 16 February, 2023. Available: https://www.rivm.nl/en/news/mpox-vaccinations-in-2022 [Accessed 3 Mar 2025].
4. New York City Department of Health and Mental Hygiene. NYC mpox Vaccination Data, 02 February, 2023. Available: https://github.com/nychealth/mpv-vaccine-data [Accessed 6 Jul 2025].
5. UK Health Security Agency. Mpox (monkeypox) vaccination recommendations, 26 August, 2022. Available from: https://www.gov.uk/government/publications/monkeypox-vaccination [Accessed 27 Jul 2025].
6. Pischel L, Martini BA, Yu N, et al. Vaccine effectiveness of 3rd generation mpox vaccines against mpox and disease severity: A systematic review and meta-analysis. *Vaccine* 2024;42:126053.
7. DC Health. DC Health Confirms First Orthopoxvirus and Potential Monkey Pox Case in the District, 5 June, 2022. Available: https://dchealth.dc.gov/release/dc-health-confirms-first-orthopoxvirus-and-potential-monkey-pox-case-district [Accessed 20 Mar 2025].
8. World Health Orginization. Monkeypox - United Kingdom of Great Britain and Northern Ireland, 18 May, 2022. Available: https://www.who.int/emergencies/disease-outbreak-news/item/2022-DON383 [Accessed 3 Mar 2025].
9. Dutch National Institute for Public Health and the Environment (RIVM). First patient with monkeypox in the Netherlands, 20 May, 2022. Available: https://www.rivm.nl/en/news/first-patient-with-monkeypox-in-netherlands [Accessed 3 Mar 2025].
10. Dutch National Institute for Public Health and the Environment (RIVM). Number of monkeypox infections continues to rise, 25 July, 2022. Available: https://www.rivm.nl/en/news/number-of-monkeypox-infections-continues-to-rise [Accessed 3 Mar 2025].
11. Kyaw NTT, Kipperman N, Alroy KA, et al. Notes from the Field: Clinical and Epidemiologic Characteristics of Mpox Cases from the Initial Phase of the Outbreak - New York City, May 19-July 15, 2022. *MMWR Morb Mortal Wkly Rep* 2022;71:1631-33.
12. County of Los Angeles public health. Los Angeles County Confirms First Presumptive Case of Monkeypox, 02 June, 2022. Available: http://publichealth.lacounty.gov/phcommon/public/media/mediapubdetail.cfm?unit=media&ou=ph&prog=media&cur=cur&prid=3884&row=25&start=1 [Accessed 20 May 2025].
13. Cuomo-Dannenburg G, McCain K, McCabe R, et al. Marburg virus disease outbreaks, mathematical models, and disease parameters: a systematic review. *Lancet Infect Dis* 2024;24:e307-e17.
14. Kucharski AJ, Edmunds WJ. Characterizing the transmission potential of zoonotic infections from minor outbreaks. *PLoS Comput Biol* 2015;11:e1004154.
15. Blumberg S, Lloyd-Smith JO. Inference of R(0) and transmission heterogeneity from the size distribution of stuttering chains. *PLoS Comput Biol* 2013;9:e1002993.
16. Charniga K, McCollum AM, Hughes CM, et al. Updating Reproduction Number Estimates for Mpox in the Democratic Republic of Congo Using Surveillance Data. *Am J Trop Med Hyg* 2024;110:561-68.
17. Fine PE, Jezek Z, Grab B, et al. The transmission potential of monkeypox virus in human populations. *Int J Epidemiol* 1988;17:643-50.
18. Hilton J, Hall I. A beta-Poisson model for infectious disease transmission. *PLoS Comput Biol* 2024;20:e1011856.
19. Grant R, Nguyen LL, Breban R. Modelling human-to-human transmission of monkeypox. *Bull World Health Organ* 2020;98:638-40.
20. Musa SS, Abdullahi ZU, Zhao S, et al. Transmission Dynamics of Monkeypox Virus in Nigeria during the Current COVID-19 Pandemic and Estimation of Effective Reproduction Number. *Vaccines (Basel)* 2022;10.
21. Gushchin VA, Ogarkova DA, Dolzhikova IV, et al. Estimation of anti-orthopoxvirus immunity in Moscow residents and potential risks of spreading Monkeypox virus. *Front Immunol* 2022;13:1023164.
22. Marziano V, Guzzetta G, Longini I, et al. Epidemiologic Quantities for Monkeypox Virus Clade I from Historical Data with Implications for Current Outbreaks, Democratic Republic of the Congo. *Emerg Infect Dis* 2024;30:2042-46.

23. World Health Orginization. Risk evaluation of clade Ib monkeypox virus: Review of evidence as of 10 December 2024, 24 January, 2025. Available: https://www.who.int/publications/m/item/risk-evaluation-of-clade-1b-monkeypox-virus-review-of-evidence-as-of-10-december-2024 [Accessed 27 Jan 2025].
24. World Health Orginization. Multi-country outbreak of mpox, External situation report#30, 25 November, 2023. Available: https://www.who.int/publications/m/item/multi-country-outbreak-of-mpox--external-situation-report-30---25-november-2023 [Accessed 27 Jan 2025].
25. Mbelambela EPS, Wandja AJP, Villanueva AF, et al. Clinical characteristics of suspected cases of human mpox (monkeypox) in Katako-Kombe, Democratic Republic of the Congo 2023: challenges and key responses. *Eur J Clin Microbiol Infect Dis* 2025;44:609-17.
26. World Health Orginization. Mpox - Democratic Republic of the Congo, 14 June, 2024. Available: https://www.who.int/emergencies/disease-outbreak-news/item/2024-DON522 [Accessed 27 Jan 2025].
27. Lin YC, Wen TH, Shih WL, et al. Impact of vaccination and high-risk group awareness on the mpox epidemic in the United States, 2022-2023: a modelling study. *EClinicalMedicine* 2024;68:102407.
28. Clay PA, Asher JM, Carnes N, et al. Modelling the impact of vaccination and sexual behaviour adaptations on mpox cases in the USA during the 2022 outbreak. *Sex Transm Infect* 2024;100:70-76.
29. Brand SPC, Cavallaro M, Cumming F, et al. The role of vaccination and public awareness in forecasts of Mpox incidence in the United Kingdom. *Nat Commun* 2023;14:4100.
30. Zhang XS, Mandal S, Mohammed H, et al. Transmission dynamics and effect of control measures on the 2022 outbreak of mpox among gay, bisexual, and other men who have sex with men in England: a mathematical modelling study. *Lancet Infect Dis* 2024;24:65-74.
31. Liang C, Suen SC, Hong C, et al. A Microsimulation Model of Mpox in Los Angeles County: Implications for Future Disease Prevention and Control Strategies among Men Who Have Sex with Men. *Open Forum Infect Dis* 2024;11:S137-s45.
32. Zhang W, Zhang J, Liu QH, et al. Behavior changes influence mpox transmission in the United States, 2022-2023: Insights from homogeneous and heterogeneous models. *PNAS Nexus* 2025;4:pgaf025.
33. Xiridou M, Miura F, Adam P, et al. The Fading of the Mpox Outbreak Among Men Who Have Sex With Men: A Mathematical Modelling Study. *J Infect Dis* 2024;230:e121-e30.
34. Guzzetta G, Marziano V, Mammone A, et al. The decline of the 2022 Italian mpox epidemic: Role of behavior changes and control strategies. *Nat Commun* 2024;15:2283.
35. Milwid RM, Li M, Fazil A, et al. Exploring the dynamics of the 2022 mpox outbreak in Canada. *J Med Virol* 2023;95:e29256.
36. Xiu F, Doyle CM, Anato JLF, et al. Impact of interventions on mpox transmission during the 2022 outbreak in Canada: a mathematical modeling study of three different cities. *Int J Infect Dis* 2025;153:107792.
37. Xiong Z, Xue L, Li X, et al. Assessing vaccine strategies for mpox outbreak in New York City using an age-structure model. *BMC Infect Dis* 2024;24:1078.
38. Clay PA, Pollock ED, Saldarriaga EM, et al. Modeling the Impact of Vaccine Dose Prioritization Strategies During the 2022 Mpox Outbreak. *Am J Epidemiol* 2025.
39. Shamier MC, Zaeck LM, Götz HM, et al. Scenarios of future mpox outbreaks among men who have sex with men: a modelling study based on cross-sectional seroprevalence data from the Netherlands, 2022. *Euro Surveill* 2024;29.
40. Dimitrov D, Adamson B, Matrajt L. Evaluation of mpox vaccine dose-sparing strategies. *PNAS Nexus* 2023;2:pgad095.
41. Knight J, Tan DHS, Mishra S. Maximizing the impact of limited vaccine supply under different early epidemic conditions: a 2-city modelling analysis of monkeypox virus transmission among men who have sex with men. *Cmaj* 2022;194:E1560-e67.
42. Gan G, Janhavi A, Tong G, et al. The need for pre-emptive control strategies for mpox in Asia and Oceania. *Infect Dis Model* 2024;9:214-23.
43. Savinkina A, Chitwood M, Kwon J, et al. Planning for Mpox on a College Campus: A Model-Based Decision-Support Tool. *Ann Intern Med* 2023;176:340-47.
44. Huang Q, Sun Y, Jia M, et al. An effectiveness study of vaccination and quarantine combination strategies for containing mpox transmission on simulated college campuses. *Infect Dis Model* 2024;9:805-15.
45. Bragazzi NL, Han Q, Iyaniwura SA, et al. Adaptive changes in sexual behavior in the high-risk population in response to human monkeypox transmission in Canada can help control the outbreak: Insights from a two-group, two-route epidemic model. *J Med Virol* 2023;95:e28575.
46. Cao Y, Fang W, Chen Y, et al. Simulating the impact of optimized prevention and control measures on the transmission of monkeypox in the United States: A model-based study. *J Med Virol* 2024;96:e29419.
47. Chaturvedi M, Rodiah I, Kretzschmar M, et al. Estimating the relative importance of epidemiological and behavioural parameters for epidemic mpox transmission: a modelling study. *BMC Med* 2024;22:297.
48. Chitwood MH, Kwon J, Savinkina A, et al. Estimated Testing, Tracing, and Vaccination Targets for Containment of the US Mpox Outbreak. *JAMA Netw Open* 2023;6:e2250984.

49. Cordeiro R, Caetano CP, Sobral D, et al. Viral genetics and transmission dynamics in the second wave of mpox outbreak in Portugal and forecasting public health scenarios. *Emerg Microbes Infect* 2024;13:2412635.
50. Das HK. Exploring the dynamics of monkeypox transmission with data-driven methods and a deterministic model. *Front Epidemiol* 2024;4:1334964.
51. Idisi OI, Yusuf TT, Adeniyi E, et al. A new compartmentalized epidemic model to analytically study the impact of awareness on the control and mitigation of the monkeypox disease. *Healthcare Analytics* 2023;4:100267.
52. Khan MA, DarAssi MH, Ahmad I, et al. The transmission dynamics of an infectious disease model in fractional derivative with vaccination under real data. *Comput Biol Med* 2024;181:109069.
53. Ko Y, Mendoza VM, Mendoza R, et al. Estimation of monkeypox spread in a nonendemic country considering contact tracing and self-reporting: A stochastic modeling study. *J Med Virol* 2023;95:e28232.
54. Leandry L, Mureithi EW. An investigation on the Monkeypox virus dynamics in human and rodent populations for a deterministic mathematical model. *Informatics in Medicine Unlocked* 2023;41:101325.
55. Musafir RR, Suryanto A, Darti I, et al. Stability analysis of a fractional-order monkeypox epidemic model with quarantine and hospitalization. *Journal of Biosafety and Biosecurity* 2024;6:34-50.
56. Van Dijck C, Hens N, Kenyon C, et al. The Roles of Unrecognized Mpox Cases, Contact Isolation and Vaccination in Determining Epidemic Size in Belgium: A Modeling Study. *Clin Infect Dis* 2023;76:e1421-e23.
57. Spicknall IH, Pollock ED, Clay PA, et al. Modeling the Impact of Sexual Networks in the Transmission of Monkeypox virus Among Gay, Bisexual, and Other Men Who Have Sex with Men - United States, 2022. *MMWR Morb Mortal Wkly Rep* 2022;71:1131-35.
58. Yang S, Guo X, Zhao Z, et al. Possibility of mpox viral transmission and control from high-risk to the general population: a modeling study. *BMC Infect Dis* 2023;23:119.
59. Yuan P, Tan Y, Yang L, et al. Modeling vaccination and control strategies for outbreaks of monkeypox at gatherings. *Front Public Health* 2022;10:1026489.
60. Yuan P, Tan Y, Yang L, et al. Assessing transmission risks and control strategy for monkeypox as an emerging zoonosis in a metropolitan area. *J Med Virol* 2023;95:e28137.
61. Jia Q, Xue L, Sui R, et al. Modelling the impact of human behavior using a two-layer Watts-Strogatz network for transmission and control of Mpox. *BMC Infect Dis* 2024;24:351.
62. Omame A, Han Q, Iyaniwura SA, et al. Understanding the impact of HIV on mpox transmission in the MSM population: A mathematical modeling study. *Infect Dis Model* 2024;9:1117-37.
63. Omame A, Iyaniwura SA, Han Q, et al. Dynamics of Mpox in an HIV endemic community: A mathematical modelling approach. *Math Biosci Eng* 2025;22:225-59.
64. Savinkina A, Kindrachuk J, Bogoch, II, et al. Modelling vaccination approaches for mpox containment and mitigation in the Democratic Republic of the Congo. *Lancet Glob Health* 2024;12:e1936-e44.
65. Jin S, Gan G, Endo A, et al. Modelling the potential spread of Clade Ib MPXV in Asian cities. *BMJ Public Health* 2025;3:e002285.
66. Murayama H, Pearson CAB, Abbott S, et al. Accumulation of Immunity in Heavy-Tailed Sexual Contact Networks Shapes Mpox Outbreak Sizes. *J Infect Dis* 2024;229:59-63.
67. Desingu PA, Rubeni TP, Nagarajan K, et al. Molecular evolution of 2022 multi-country outbreak-causing monkeypox virus Clade IIb. *iScience* 2024;27:108601.
68. Isidro J, Borges V, Pinto M, et al. Phylogenomic characterization and signs of microevolution in the 2022 multi-country outbreak of monkeypox virus. *Nat Med* 2022;28:1569-72.
69. Xiu F, Flores Anato JL, Cox J, et al. Characteristics of the Sexual Networks of Men Who Have Sex With Men in Montréal, Toronto, and Vancouver: Insights from Canada's 2022 Mpox Outbreak. *J Infect Dis* 2024;229:S293-s304.
70. Sun YQ, Chen JJ, Liu MC, et al. Mapping global zoonotic niche and interregional transmission risk of monkeypox: a retrospective observational study. *Global Health* 2023;19:58.
71. Guzzetta G, Mammone A, Ferraro F, et al. Early Estimates of Monkeypox Incubation Period, Generation Time, and Reproduction Number, Italy, May-June 2022. *Emerg Infect Dis* 2022;28:2078-81.
72. Miura F, Backer JA, van Rijckevorsel G, et al. Time Scales of Human Mpox Transmission in The Netherlands. *J Infect Dis* 2024;229:800-04.
73. Althobaity YM, Tildesley MJ. Exploring the epidemiological characteristics of Mpox in the Arab Gulf countries. *Sci Rep* 2025;15:15748.
74. Paredes MI, Ahmed N, Figgins M, et al. Underdetected dispersal and extensive local transmission drove the 2022 mpox epidemic. *Cell* 2024;187:1374-86.e13.
75. Zheng Q, Bao C, Memish ZA, et al. Probability of Mpox importation during the FIFA World Cup 2022 in Qatar. *J Infect Public Health* 2023;16:264-65.
76. Guo Z, Zhao S, Sun S, et al. Estimation of the serial interval of monkeypox during the early outbreak in

2022. *J Med Virol* 2023;95:e28248.
77. Betti MI, Farrell L, Heffernan J. A pair formation model with recovery: Application to mpox. *Epidemics* 2023;44:100693.
78. Liu T, Yang S, Luo B, et al. Anticipating the transmissibility of the 2022 mpox outbreak. *J Med Virol* 2023;95:e28683.
79. Wei F, Peng Z, Jin Z, et al. Study and prediction of the 2022 global monkeypox epidemic. *J Biosaf Biosecur* 2022;4:158-62.
80. Al-Raeei M. The study of human monkeypox disease in 2022 using the epidemic models: herd immunity and the basic reproduction number case. *Ann Med Surg (Lond)* 2023;85:316-21.
81. Gao J, Zhou C, Liang H, et al. Monkeypox outbreaks in the context of the COVID-19 pandemic: Network and clustering analyses of global risks and modified SEIR prediction of epidemic trends. *Front Public Health* 2023;11:1052946.
82. Al-Raeei M. The basic reproductive ratio of the 2022 outbreak of the monkey pox virus disease for the United Kingdom, Canada, Brazil, the United Arab Emirates, and Nigeria. *Beni Suef Univ J Basic Appl Sci* 2022;11:135.
83. Akhmetzhanov AR, Wu PH. Transmission potential of mpox in mainland China, June-July 2023: estimating reproduction number during the initial phase of the epidemic. *PeerJ* 2024;12:e16908.
84. Augsburger IB, Galanthay GK, Tarosky JH, et al. Voluntary vaccination may not stop monkeypox outbreak: A game-theoretic model. *PLoS Negl Trop Dis* 2022;16:e0010970.
85. Borges V, Duque MP, Martins JV, et al. Viral genetic clustering and transmission dynamics of the 2022 mpox outbreak in Portugal. *Nat Med* 2023;29:2509-17.
86. Branda F, Pierini M, Mazzoli S. Monkeypox: Early estimation of basic reproduction number R(0) in Europe. *J Med Virol* 2023;95:e28270.
87. Du Z, Shao Z, Bai Y, et al. Reproduction number of monkeypox in the early stage of the 2022 multi-country outbreak. *J Travel Med* 2022;29.
88. Kim M, Shim E. Assessing the transmission potential of mpox in East Asia during 2022-2023: A focus on Taiwan, China, Japan, and South Korea. *Int J Infect Dis* 2024;138:110-12.
89. Kwok KO, Wei WI, Tang A, et al. Estimation of local transmissibility in the early phase of monkeypox epidemic in 2022. *Clin Microbiol Infect* 2022;28:1653.e1-53.e3.
90. Liao LC, Hsu CY, Chen HH, et al. Estimating the Global Spread of Epidemic Human Monkeypox with Bayesian Directed Acyclic Graphic Model. *Vaccines (Basel)* 2023;11.
91. Marín-Sánchez O, Pesantes-Grados P, Pérez-Timaná L, et al. Comparative Epidemiological Assessment of Monkeypox Infections on a Global and Continental Scale Using Logistic and Gompertz Mathematical Models. *Vaccines (Basel)* 2023;11.
92. Quispe AM, Castagnetto JM. Monkeypox in Latin America and the Caribbean: assessment of the first 100 days of the 2022 outbreak. *Pathog Glob Health* 2023;117:717-26.
93. Saldaña F, Daza-Torres ML, Aguiar M. Data-driven estimation of the instantaneous reproduction number and growth rates for the 2022 monkeypox outbreak in Europe. *PLoS One* 2023;18:e0290387.
94. Schrarstzhaupt IN, Fontes-Dutra M, Diaz-Quijano FA. Early estimates of the incidence trend and the reproductive number of the monkeypox epidemic in Brazil. *Travel Med Infect Dis* 2022;50:102484.
95. Yang S, Guo X, Zhao Z, et al. Global differences in natural transmission rates of Monkeypox virus. *J Infect* 2022;85:702-69.
96. Zygmunt A, Saunders A, Navarro C, et al. Mpox outbreak control indicators used in Ontario, Canada: May 21-December 10, 2022. *J Med Virol* 2023;95:e29251.
97. Bragazzi NL, Iyaniwura SA, Han Q, et al. Quantifying the basic reproduction number and underestimated fraction of Mpox cases worldwide at the onset of the outbreak. *J R Soc Interface* 2024;21:20230637.
98. Díaz-Brochero C, Cucunubá ZM. Epidemiological findings, estimates of the instantaneous reproduction number, and control strategies of the first Mpox outbreak in Latin America. *Travel Med Infect Dis* 2024;59:102701.
99. Zhang Y, Zhou X. Supercritical and homogenous transmission of monkeypox in the capital of China. *J Med Virol* 2024;96:e29442.
